# Monitoring of adult emergence in the pine processionary moth between 1970 and 1984 in Mont Ventoux, France

**DOI:** 10.3897/BDJ.9.e61086

**Published:** 2021-02-17

**Authors:** Jean-Claude Martin, Jean-Pierre Rossi, Maurane Buradino, Carole Kerdelhué

**Affiliations:** 1 INRAE, UEFM, Avignon, France INRAE, UEFM Avignon France; 2 INRAE, CBGP, Montpellier, France INRAE, CBGP Montpellier France

## Abstract

**Background:**

The current climate change has marked impacts on the phenology of species, i.e. the timing of the various stages of their life cycle. Yet, to fully understand how phenological patterns can be modified according to changes in temperature regimes, it is of prime importance to rely on high quality historical data. Here, we propose a very valuable dataset including individual monitoring from pupation to adult emergence of 46 479 individuals of pine processionary moth (*Thaumetopoea pityocampa*) surveyed between 1970 and 1984 in southern France along an altitudinal gradient. As optional prolonged diapause occurs in this species, i.e. some individuals experience one or more years of diapause before emerging, the caterpillars sampled in any given year were monitored during up to 5 years. The goal was to give precise information about phenology in this species to further analyse its temporal patterns of variation.

**New information:**

This dataset is unique by its richness and the type of data it contains. Phenology in the pine processionary moth is often monitored by the use of pheromone traps in the field, which does not provide all the necessary information, because it is then not possible to trace back the exact origin of the moth trapped, nor to characterise other steps of the life cycle. Moreover, as it corresponds to historical data dating back to the 70s and the 80s, the dataset provides a historical baseline of trends in the pre-warming period.

## Introduction

Widely-distributed species encounter different ecological pressures throughout their range and their phenology can be locally tuned by adaptation to optimise resource use and climatic conditions faced by each life stage (Abarca and Lill 2019). In the recent years, climate change has strongly affected the phenology of many organisms, but the direction and strength of these responses proved to vary among species (Maurer et al. 2018). To analyse how species react to temperature changes, it is necessary to rely on time series that allow us to finely explore the relationship between yearly variations and the timing of major life cycle steps.

The pine processionary moth (PPM), *Thaumetopoea pityocampa* (Lepidoptera: Notodontidae), is a univoltine species that reproduces in summer, immediately after adult emergence. Its larvae hatch and develop throughout autumn and winter in typical white tents built in conifer branches. At the end of larval development, they leave their host tree in head-to-tail procession in search of a suitable underground pupation site, where they stay until adult emergence the following summer. A proportion of the individuals can enter a so-called prolonged pupal diapause (semi-voltine cycle). In such a case, the pupa does not emerge as an adult the following summer, but remains in diapause for one or more complete year(s) and emerges one or several years after the other individuals (Battisti et al. 2015). The timing of adult emergence and sexual reproduction is dependent on local environmental conditions. It tends to occur earlier in northern sites and at high altitude, while it occurs later in southern sites at low altitude (Huchon and Démolin 1970). On the contrary, little is known about the drivers of yearly variation linked to temperatures in this species.

We here present an unprecedented dataset corresponding to a 15-year field monitoring experiment, documenting both individual dates of procession and dates of emergence in six sites located along an altitudinal gradient in southern France. We sampled 46 479 caterpillars when they were leaving their host tree at the end of larval development. Each individual was placed in laboratory conditions at room temperature until moth emergence. The dataset does not provide information about the sex of the monitored individuals nor about mortality factors. This individual-based dataset brings original information and allows analyses that cannot be conducted with classical pheromone trapping data. Yet, note that the protocol used forced larvae to pupate in laboratory conditions and that the emergence data we provide cannot directly be compared to data from pheromone traps in the field.

## Sampling methods

### Sampling description

**Field sampling**

At each study site, 10 Austrian pine trees (*Pinus nigra*) were selected and fitted with a wire and a net strapped on the trunk down to the ground to trap the caterpillars leaving the tree in search of a pupation site and to prevent them from burying themselves. When PPM density was very low, nests were sampled on other trees in the vicinity and grafted to the selected pines. All the sites were visited every day from 1 January to 31 May each year. The caterpillars trapped at the base of each tree were thus sampled and counted every day. The larvae were then separated, placed individually in a glass tube and identified with a unique code. Sampling date and location were recorded for each individual. Caterpillars sampled from the same tree at the same date were identified as belonging to the same batch; note that they might originate from different nests of the same tree and thus belong to several families.

**Monitoring of adult emergence**

The sampled larvae were brought back every day to Malaucène (altitude 340 m a.s.l.) and left in a non-heated laboratory under natural photoperiods at room temperature (not recorded). As larvae do not feed at this stage, no pine needles were provided. A month after pupation, cocoons were taken from the tubes and were put into plastic boxes filled with untreated sawdust; individuals from the same batch were grouped in the same box, with 100 individuals maximum per box. The boxes were checked daily from 1 June to 30 September, to record emergences. In case all individuals did not emerge, the box was monitored again the following year to allow recording of the date of emergence of the diapausing pupae, until the fifth year.

## Geographic coverage

### Description

**Figure 1.**
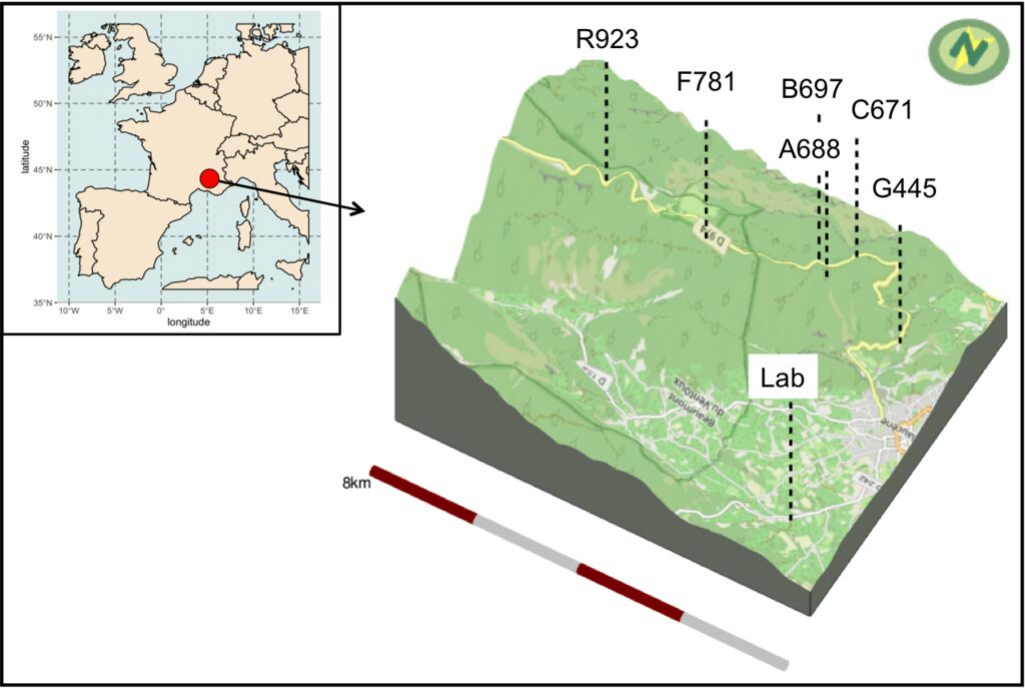
3D view of the six sites along the altitudinal gradient and location of the laboratory where the pupae were monitored until emergence. The map in the top left corner shows the location of the study site in France.

The experimental design took place along an altitudinal gradient in Mont Ventoux, France. The sampling sites are detailed in Table 1. The laboratory, where the larvae were allowed to pupate and were monitored daily, was situated in Malaucène (longitude 5.140, latitude 44.192 and altitude 340 m a.s.l.). The sites and the laboratory can be seen in Fig. 1.

## Taxonomic coverage

### Description

The study concerns the pine processionary moth *Thaumetopoea pityocampa* (Denis & Schiffermüller) (Lepidoptera: Notodontidae) sampled from the black pine *Pinus nigra* (Arnold).

## Temporal coverage

### Notes

The study took place each year between 1970 and 1984. Caterpillars were sampled in the field every day between 1 January and 31 May and adult emergence was monitored between 1 June and 30 September.

## Usage licence

### Usage licence

Creative Commons Public Domain Waiver (CC-Zero)

## Data resources

### Data package title

Identification, date of sampling and date of adult emergence in the six study sites

### Resource link

https://data.inrae.fr/dataset.xhtml?persistentId=doi: 10.15454/FGRKAY

### Number of data sets

6

### Data set 1.

#### Data set name

Emergence data for site G445 (file name: SiteG_EmergenceData.csv)

#### Data format

csv

#### Number of columns

7

#### 

**Data set 1.** 

Column label	Column description
site	Site code, refers to Table 1
year of sampling	Year when the caterpillars were sampled while in procession. Format yy
batch	Unique batch identifier (1 batch = one group of caterpillars sampled at the same time from the same tree). The unique code is constructed as site-year-number
date of sampling	Day when the caterpillars from the batch identified in the previous column were sampled and brought back to the lab. Format yyyy-mm-dd
year of emergence	Year when adults emerged in the lab. Format yyyy
date of emergence	Day of the control of emergence (everyday between 1 June and 30 September each year after sampling). Format yyyy-mm-dd
number of individuals emerged	Number of adults emerged at the corresponding date

### Data set 2.

#### Data set name

Emergence data for site C671 (file name: SiteC_EmergenceData.csv)

#### Data format

csv

#### Number of columns

7

#### 

**Data set 2.** 

Column label	Column description
site	Site code, refers to Table 1
year of sampling	Year when the caterpillars were sampled while in procession. Format yy
batch	Unique batch identifier (1 batch = one group of caterpillars sampled at the same time from the same tree). The unique code is constructed as site-year-number
date of sampling	Day when the caterpillars from the batch identified in the previous column were sampled and brought back to the lab. Format yyyy-mm-dd
year of emergence	Year when adults emerged in the lab. Format yyyy
date of emergence	Day of the control of emergence (everyday between 1 June and 30 September each year after sampling). Format yyyy-mm-dd
number of individuals emerged	Number of adults emerged at the corresponding date

### Data set 3.

#### Data set name

Emergence data for site A688 (file name: SiteA_EmergenceData.csv)

#### Data format

csv

#### Number of columns

7

#### 

**Data set 3.** 

Column label	Column description
site	Site code, refers to Table 1
year of sampling	Year when the caterpillars were sampled while in procession. Format yy
batch	Unique batch identifier (1 batch = one group of caterpillars sampled at the same time from the same tree). The unique code is constructed as site-year-number
date of sampling	Day when the caterpillars from the batch identified in the previous column were sampled and brought back to the lab. Format yyyy-mm-dd
year of emergence	Year when adults emerged in the lab. Format yyyy
date of emergence	Day of the control of emergence (everyday between 1 June and 30 September each year after sampling). Format yyyy-mm-dd
number of individuals emerged	Number of adults emerged at the corresponding date

### Data set 4.

#### Data set name

Emergence data for site B697 (file name: SiteB_EmergenceData.csv)

#### Data format

csv

#### Number of columns

7

#### 

**Data set 4.** 

Column label	Column description
site	Site code, refers to Table 1
year of sampling	Year when the caterpillars were sampled while in procession. Format yy
batch	Unique batch identifier (1 batch = one group of caterpillars sampled at the same time from the same tree). The unique code is constructed as site-year-number
date of sampling	Day when the caterpillars from the batch identified in the previous column were sampled and brought back to the lab. Format yyyy-mm-dd
year of emergence	Year when adults emerged in the lab. Format yyyy
date of emergence	Day of the control of emergence (everyday between 1 June and 30 September each year after sampling). Format yyyy-mm-dd
number of individuals emerged	Number of adults emerged at the corresponding date

### Data set 5.

#### Data set name

Emergence data for site F781 (file name: SiteF_EmergenceData.csv)

#### Data format

csv

#### Number of columns

7

#### 

**Data set 5.** 

Column label	Column description
site	Site code, refers to Table 1
year of sampling	Year when the caterpillars were sampled while in procession. Format yy
batch	Unique batch identifier (1 batch = one group of caterpillars sampled at the same time from the same tree). The unique code is constructed as site-year-number
date of sampling	Day when the caterpillars from the batch identified in the previous column were sampled and brought back to the lab. Format yyyy-mm-dd
year of emergence	Year when adults emerged in the lab. Format yyyy
date of emergence	Day of the control of emergence (everyday between 1 June and 30 September each year after sampling). Format yyyy-mm-dd
number of individuals emerged	Number of adults emerged at the corresponding date

### Data set 6.

#### Data set name

Emergence data for site R923 (file name: SiteR_EmergenceData.csv)

#### Data format

csv

#### Number of columns

7

#### 

**Data set 6.** 

Column label	Column description
site	Site code, refers to Table 1
year of sampling	Year when the caterpillars were sampled while in procession. Format yy
batch	Unique batch identifier (1 batch = one group of caterpillars sampled at the same time from the same tree). The unique code is constructed as site-year-number
date of sampling	Day when the caterpillars from the batch identified in the previous column were sampled and brought back to the lab. Format yyyy-mm-dd
year of emergence	Year when adults emerged in the lab. Format yyyy
date of emergence	Day of the control of emergence (everyday between 1 June and 30 September each year after sampling). Format yyyy-mm-dd
number of individuals emerged	Number of adults emerged at the corresponding date

## Additional information

We provide graphics showing the dynamics of adult emergence for each site and year, showing both individuals emerging without prolonged diapause (univoltine cycle) and those emerging after one or more years of optional prolonged diapause (semi-voltine cycle) (Suppl. materials 1, 2, 3, 4, 5, 6).

We also provide a table and a graph documenting for each site and year the numbers and percentage of individuals directly emerging and individuals experiencing one year or more of prolonged pupal diapause (Suppl. materials 7, 8).

## Supplementary Material

34A27C2C-C3CD-55CF-B93B-4724C2D0E388 10.3897/BDJ.9.e61086.suppl1 Supplementary material 1 Emergence curves for each cohort sampled in site G445 Data type Curves of daily adult emergence Brief description For each cohort (= individuals sampled the same year in the same site), the graph shows the dynamics of adult emergence. Dates of emergence are expressed as Julian days (1 January = 1 and 31 December = 365 or 366), so long-diapausing individuals are shown on the same graph in a different colour. File: oo_503427.pdf https://binary.pensoft.net/file/503427 Martin J.-C., Rossi J.-P., Buradino M. & Kerdelhué C.

B03C1073-3203-5221-8CD3-303DF636A38B 10.3897/BDJ.9.e61086.suppl2 Supplementary material 2 Emergence curves for each cohort sampled in site C671 Data type Curves of daily adult emergence Brief description For each cohort (= individuals sampled the same year in the same site), the graph shows the dynamics of adult emergence. Dates of emergence are expressed as Julian days (1 January = 1 and 31 December = 365 or 366), so long-diapausing individuals are shown on the same graph in a different colour. File: oo_503429.pdf https://binary.pensoft.net/file/503429 Martin J.-C., Rossi J.-P., Buradino M. & Kerdelhué C.

685AFB48-7B01-58B4-A3DB-695B1E58A373 10.3897/BDJ.9.e61086.suppl3 Supplementary material 3 Emergence curves for each cohort sampled in site A688 Data type Curves of daily adult emergence Brief description For each cohort (= individuals sampled the same year in the same site), the graph shows the dynamics of adult emergence. Dates of emergence are expressed as Julian days (1 January = 1 and 31 December = 365 or 366), so long-diapausing individuals are shown on the same graph in a different colour. File: oo_503430.pdf https://binary.pensoft.net/file/503430 Martin J.-C., Rossi J.-P., Buradino M. & Kerdelhué C.Martin J.-C., Rossi J.-P., Buradino M. & Kerdelhué C.

78EFA46A-1BF1-50A8-8F06-CDDAF68FBC81 10.3897/BDJ.9.e61086.suppl4 Supplementary material 4 Emergence curves for each cohort sampled in site B697 Data type Curves of daily adult emergence Brief description For each cohort (= individuals sampled the same year in the same site), the graph shows the dynamics of adult emergence. Dates of emergence are expressed as Julian days (1 January = 1 and 31 December = 365 or 366), so long-diapausing individuals are shown on the same graph in a different colour. File: oo_503431.pdf https://binary.pensoft.net/file/503431 Martin J.-C., Rossi J.-P., Buradino M. & Kerdelhué C.

1E27DC13-CE4F-57EC-999F-6EA7129B03D7 10.3897/BDJ.9.e61086.suppl5 Supplementary material 5 Emergence curves for each cohort sampled in site F781 Data type Curves of daily adult emergence Brief description For each cohort (= individuals sampled the same year in the same site), the graph shows the dynamics of adult emergence. Dates of emergence are expressed as Julian days (1 January = 1 and 31 December = 365 or 366), so long-diapausing individuals are shown on the same graph in a different colour. File: oo_503432.pdf https://binary.pensoft.net/file/503432 Martin J.-C., Rossi J.-P., Buradino M. & Kerdelhué C.

54950688-E7E1-5458-8FB0-1158C5DB32AE 10.3897/BDJ.9.e61086.suppl6 Supplementary material 6 Emergence curves for each cohort sampled in site R923 Data type Curves of daily adult emergence Brief description For each cohort (= individuals sampled the same year in the same site), the graph shows the dynamics of adult emergence. Dates of emergence are expressed as Julian days (1 January = 1 and 31 December = 365 or 366), so long-diapausing individuals are shown on the same graph in a different colour. File: oo_503433.pdf https://binary.pensoft.net/file/503433 Martin J.-C., Rossi J.-P., Buradino M. & Kerdelhué C.

1EFAC19B-8419-5684-B351-160458C084BD 10.3897/BDJ.9.e61086.suppl7 Supplementary material 7 Table showing the percentage of direct emergence and the percentage of individuals emerging after one or more years of prolonged diapause for each site and cohort Data type Table (csv) Brief description For each site (G445, C671, A688, B697, F781, R923) and each sampling year, the Table shows how many individuals emerged directly (i.e. the same year) or after 1, 2, 3 or 4 years of prolonged diapause. The last column gives the corresponding percentages. File: oo_502745.csv https://binary.pensoft.net/file/502745 Martin J.-C., Rossi J.-P., Buradino M. & Kerdelhué C.

B801E826-E832-5E77-BC55-A10E82D1F701 10.3897/BDJ.9.e61086.suppl8 Supplementary material 8 Curves showing the percentage of individuals having experienced a prolonged diapause for each site and year Data type Graph File: oo_503435.png https://binary.pensoft.net/file/503435 Martin J.-C., Rossi J.-P., Buradino M. & Kerdelhué C.

## Figures and Tables

**Table 1.** Name, geographic coordinates and altitude of the study sites.

Site name	Code	Country	State	Locality	Latitude (dd)	Longitude (dd)	Altitude
Portail St Jean	G445	France	Vaucluse	Malaucène	44.164	5.141	445 m
2ème Plateforme	C671	France	Vaucluse	Malaucène	44.159	5.153	671 m
Bramefam	B697	France	Vaucluse	Malaucène	44.162	5.158	697 m
le Camp	A688	France	Vaucluse	Malaucène	44.164	5.155	688 m
Fribouquet	F781	France	Vaucluse	Beaumont-du-Ventoux	44.168	5.177	781 m
Les Ramayettes	R923	France	Vaucluse	Beaumont-du-Ventoux	44.169	5.196	923 m
