# Supplementary material for: Monitoring of adult emergence in the pine processionary moth between 1970 and 1984 in Mont Ventoux, France
Source: Biodivers Data J. 2021 Feb 17;9:e61086. doi: 10.3897/BDJ.9.e61086 (PMC7904747; doi:10.3897/BDJ.9.e61086)
Supplement: Supplementary material 1 — Emergence curves for each cohort sampled in site G445 [file bdj-09-e61086-s001.pdf]

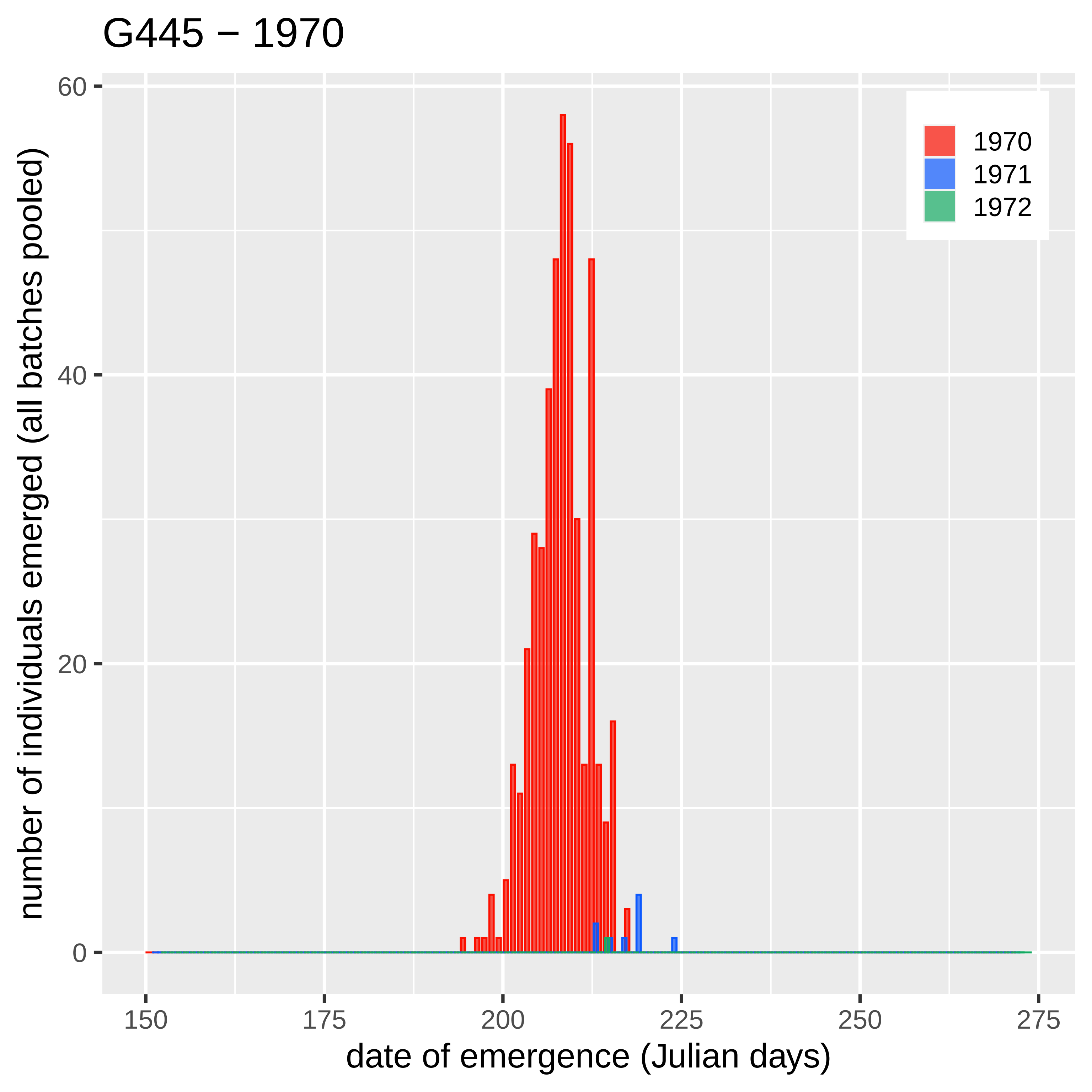

G445 − 1970
number of individuals emerged (all batches pooled)
0
20
40
60
150
175
200
225
250
275
date of emergence (Julian days)
1970
1971
1972

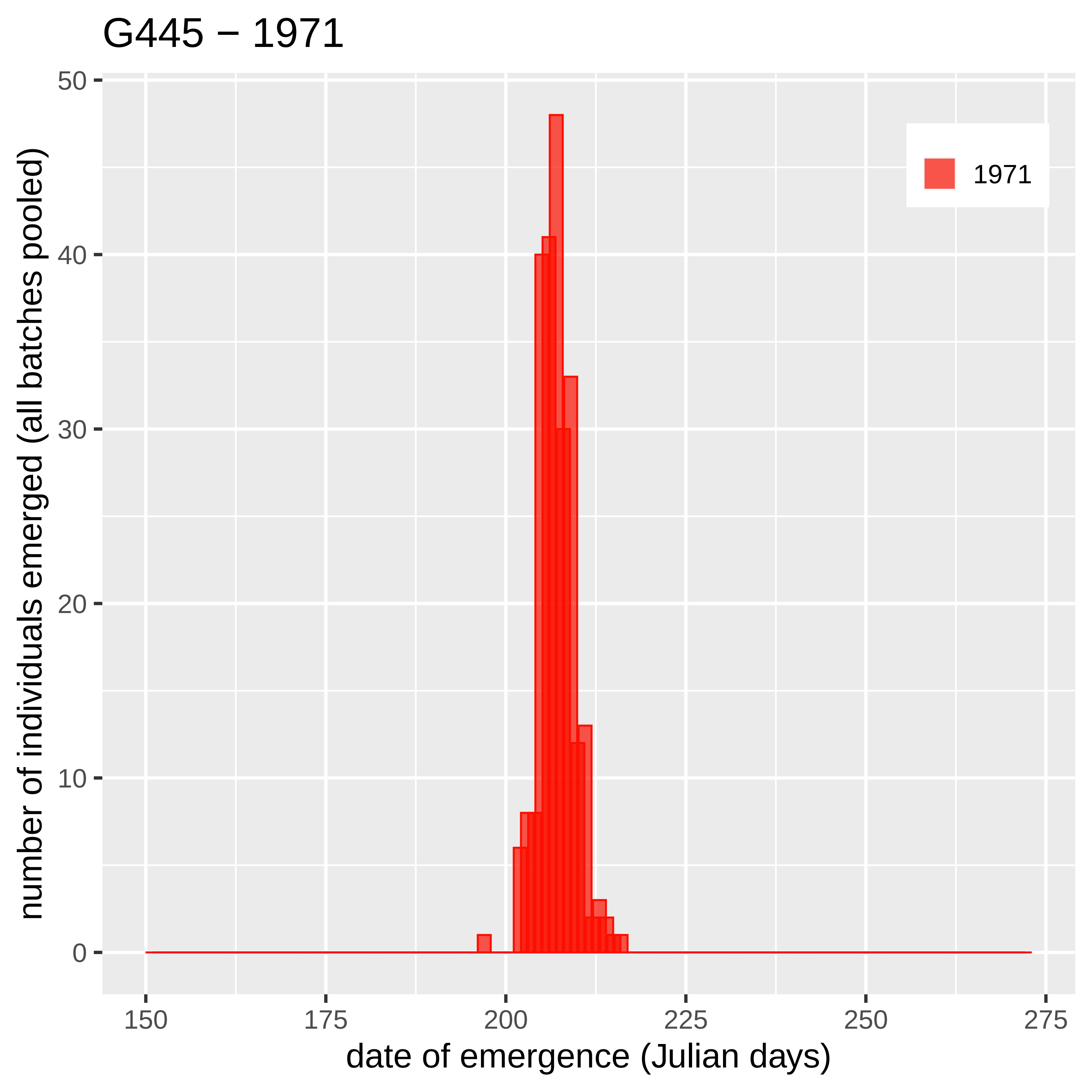

G445 − 1971
1971
number of individuals emerged (all batches pooled)
0
10
20
30
40
50
150
175
200
225
250
275
date of emergence (Julian days)

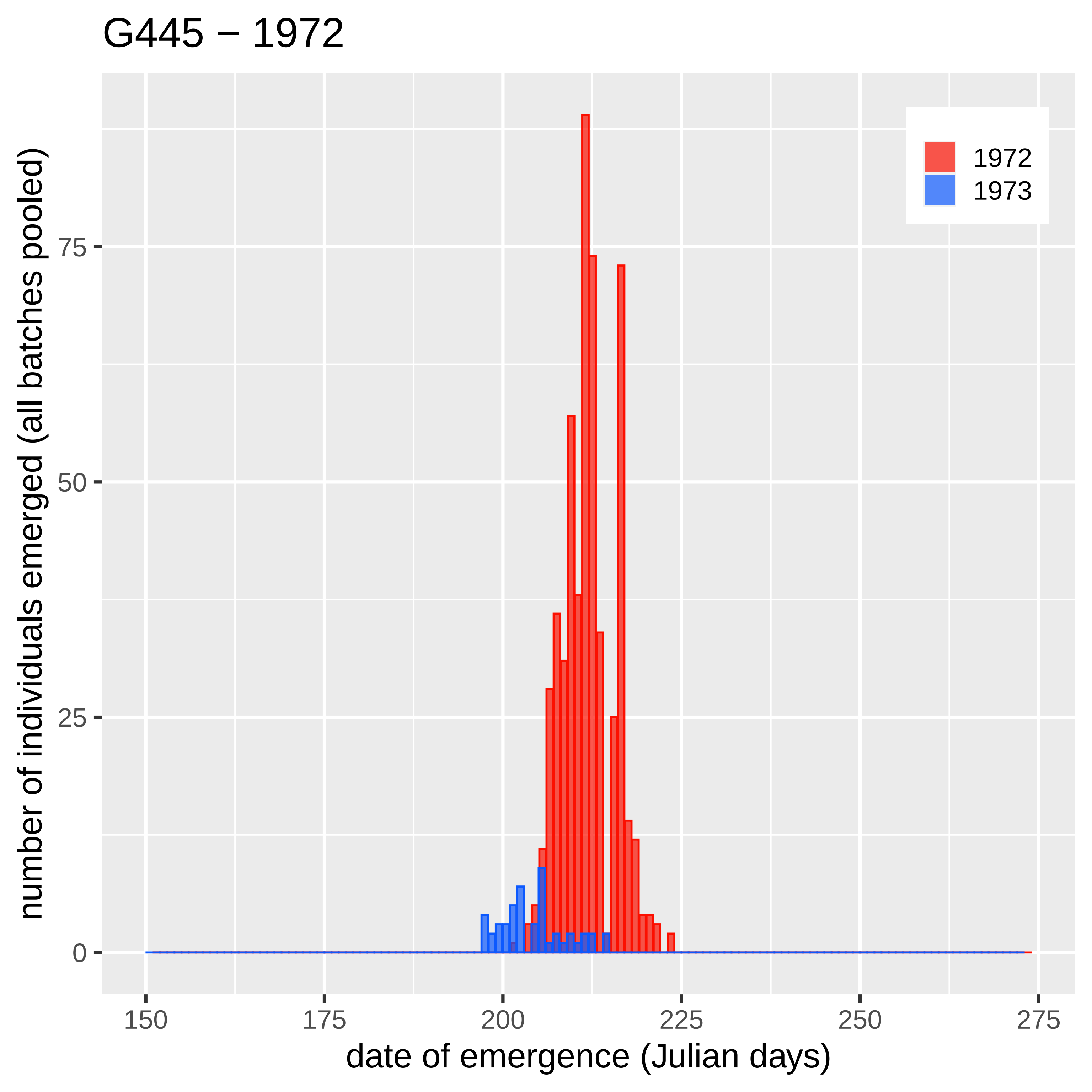
G445 − 1972
number of individuals emerged (all batches pooled)
date of emergence (Julian days)
1972
1973
0
25
50
75
150
175
200
225
250
275

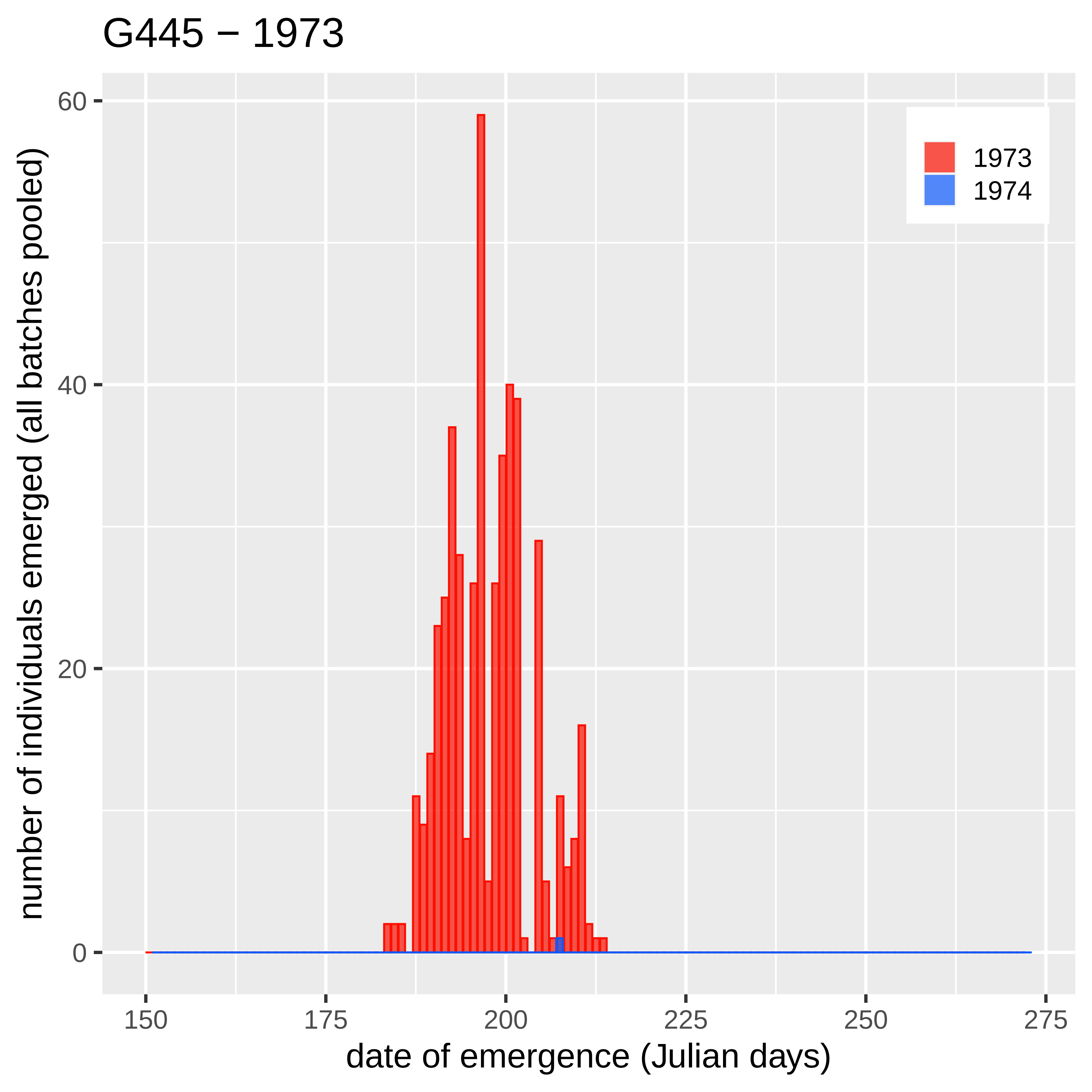
G445 − 1973
1973
1974
number of individuals emerged (all batches pooled)
0
20
40
60
150
175
200
225
250
275
date of emergence (Julian days)

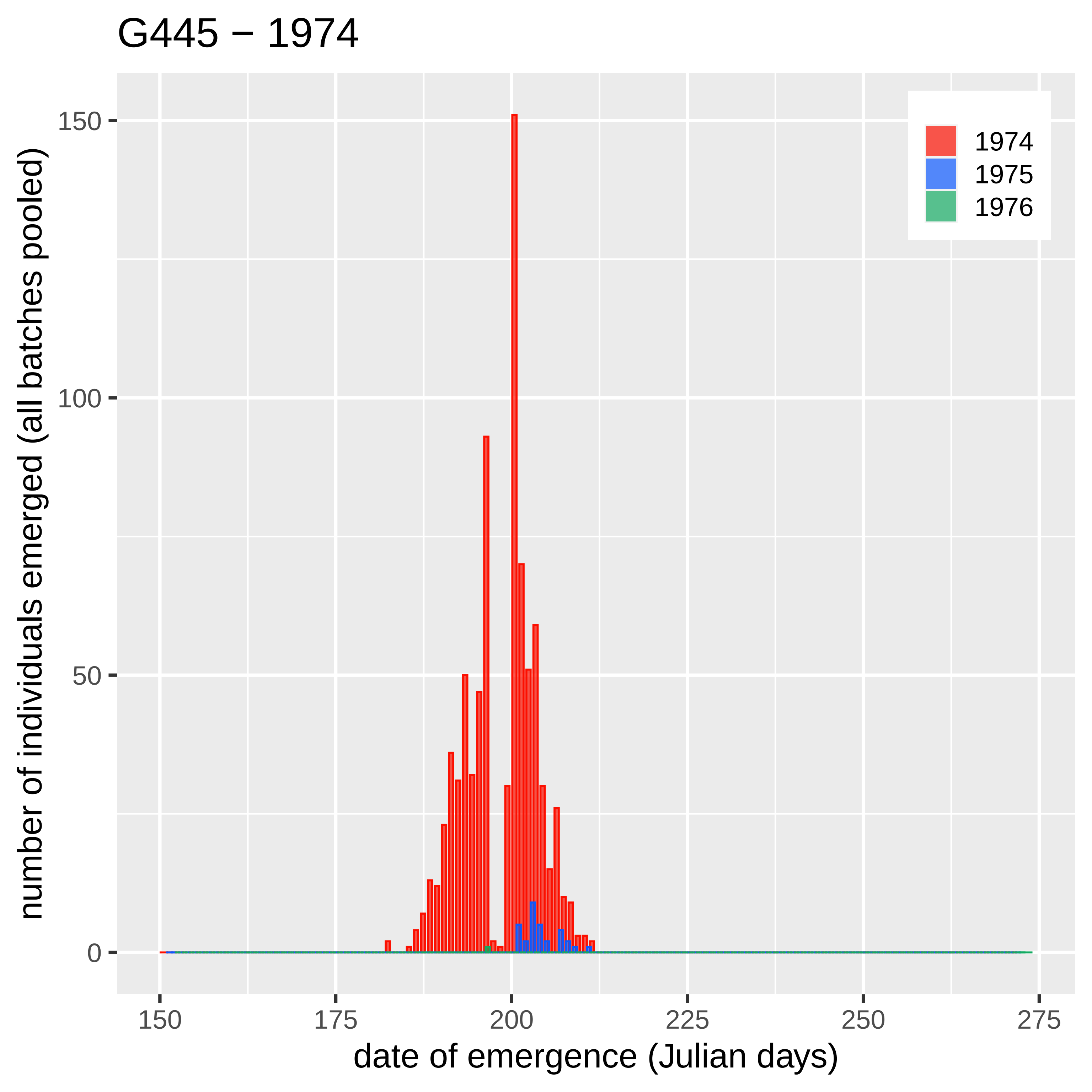

G445 − 1974
number of individuals emerged (all batches pooled)
150
100
50
0
150
175
200
225
250
275
date of emergence (Julian days)
1974
1975
1976

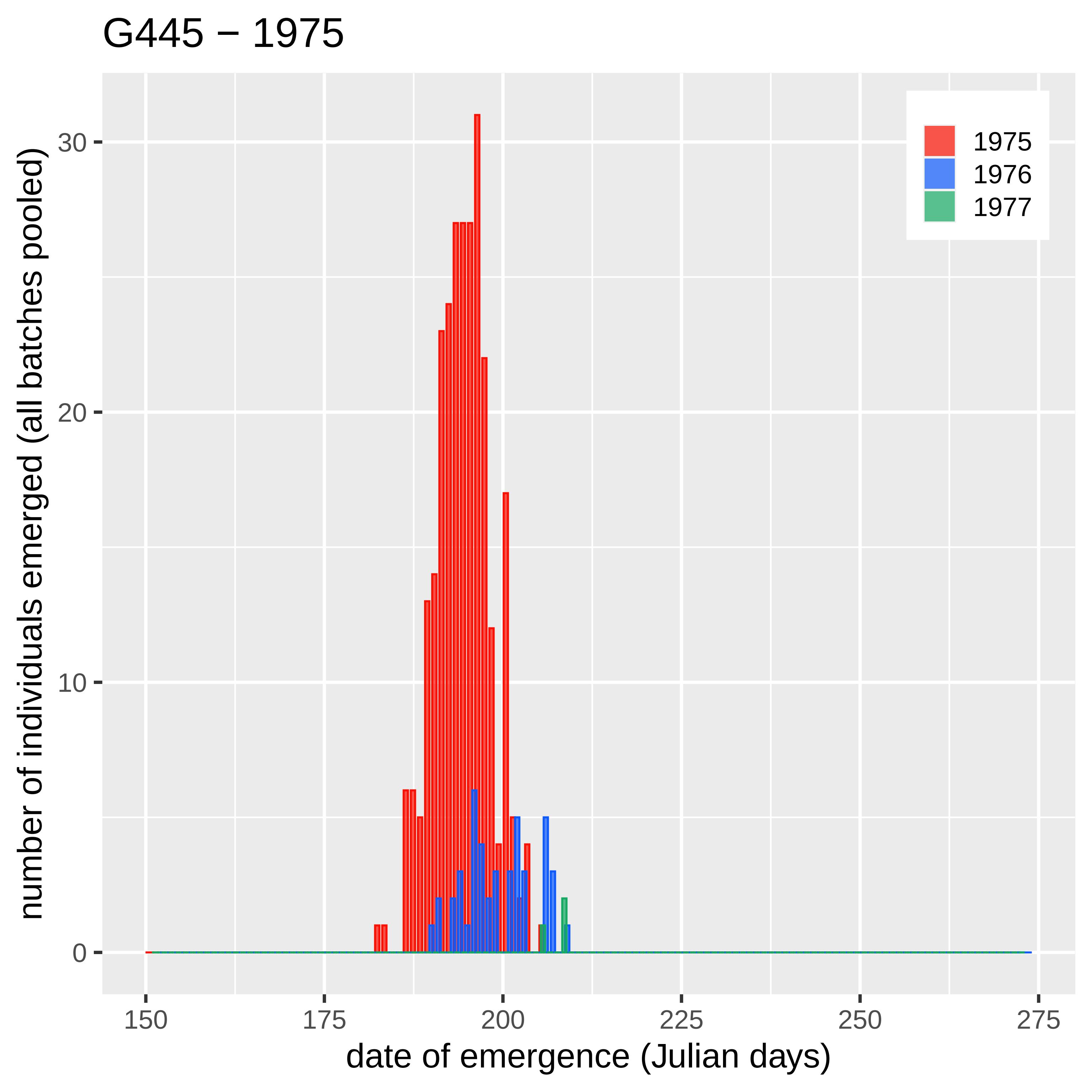

G445 − 1975
number of individuals emerged (all batches pooled)
0
10
20
30
150
175
200
225
250
275
date of emergence (Julian days)
1975
1976
1977

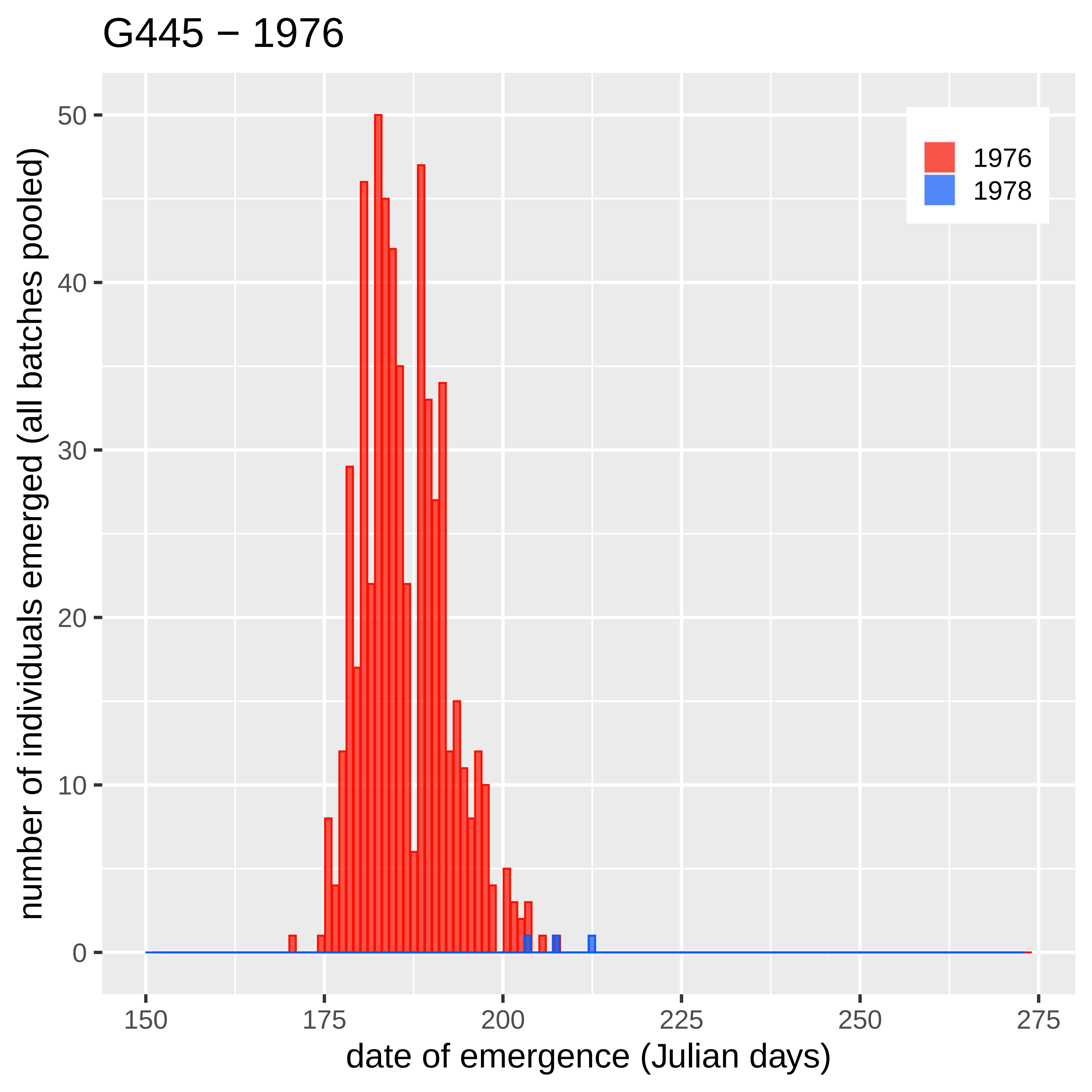
G445 − 1976
number of individuals emerged (all batches pooled)
date of emergence (Julian days)
0
10
20
30
40
50
150
175
200
225
250
275
1976
1978

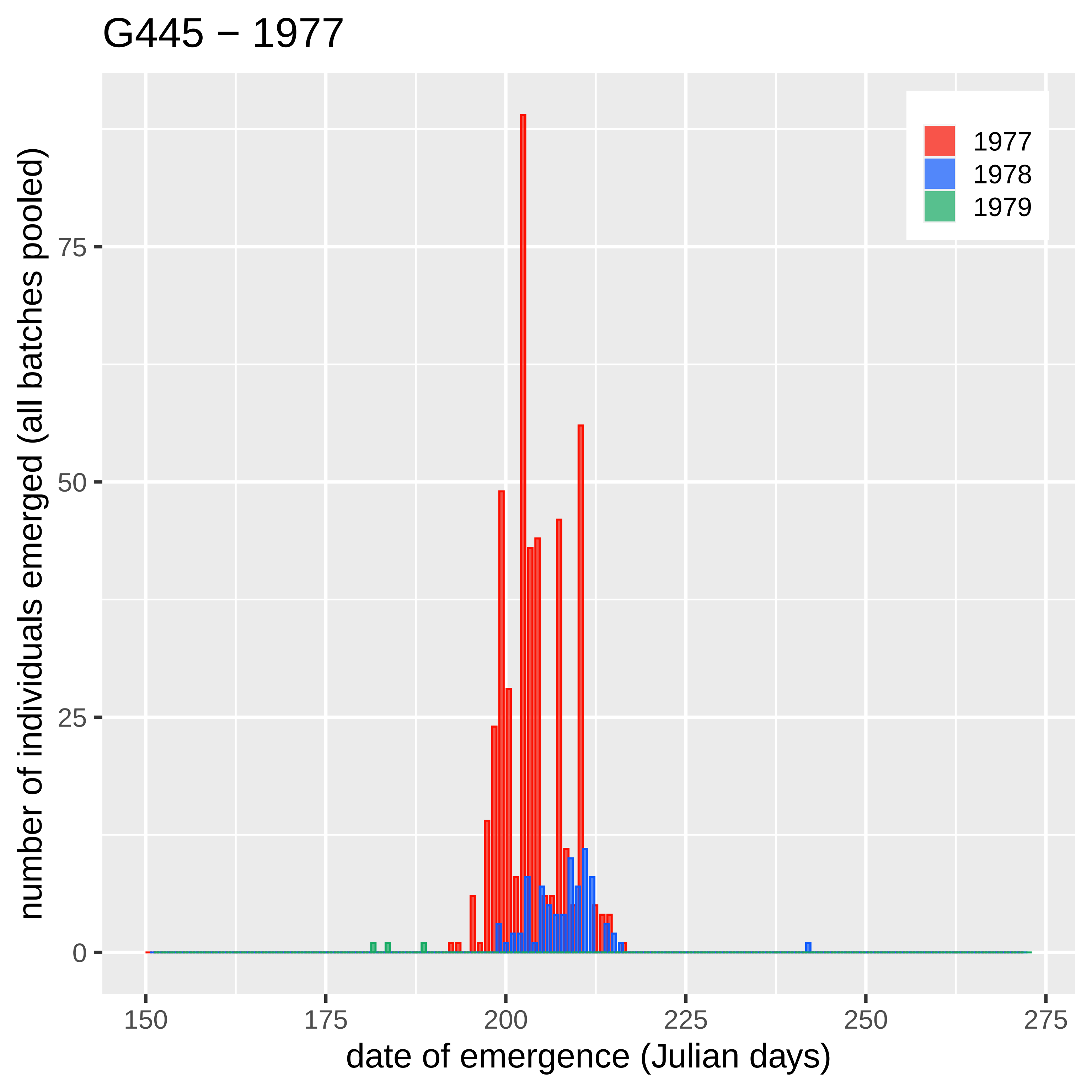

G445 − 1977
1977
1978
1979
number of individuals emerged (all batches pooled)
0
25
50
75
150
175
200
225
250
275
date of emergence (Julian days)

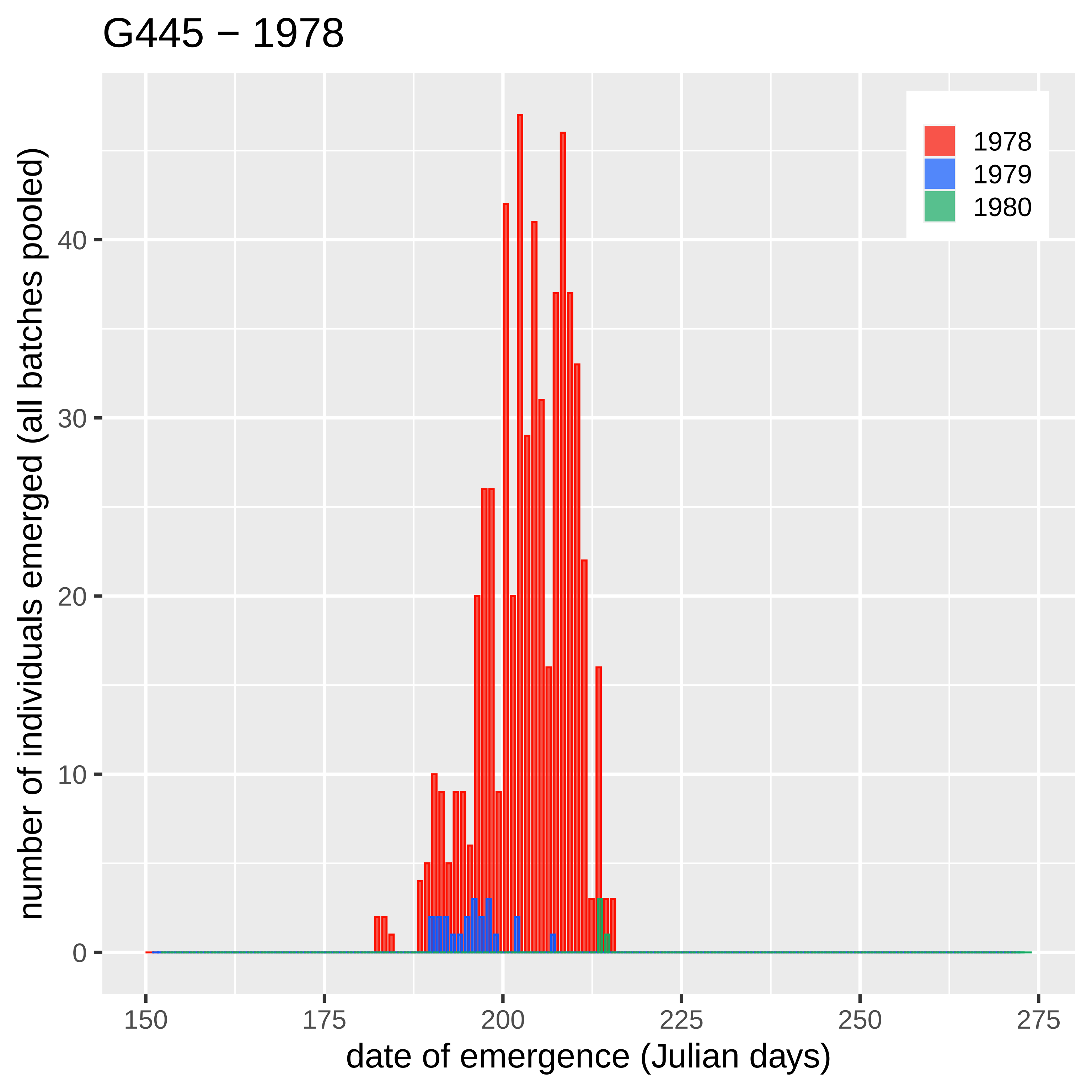

G445 − 1978
1978
1979
1980
number of individuals emerged (all batches pooled)
0
10
20
30
40
150
175
200
225
250
275
date of emergence (Julian days)

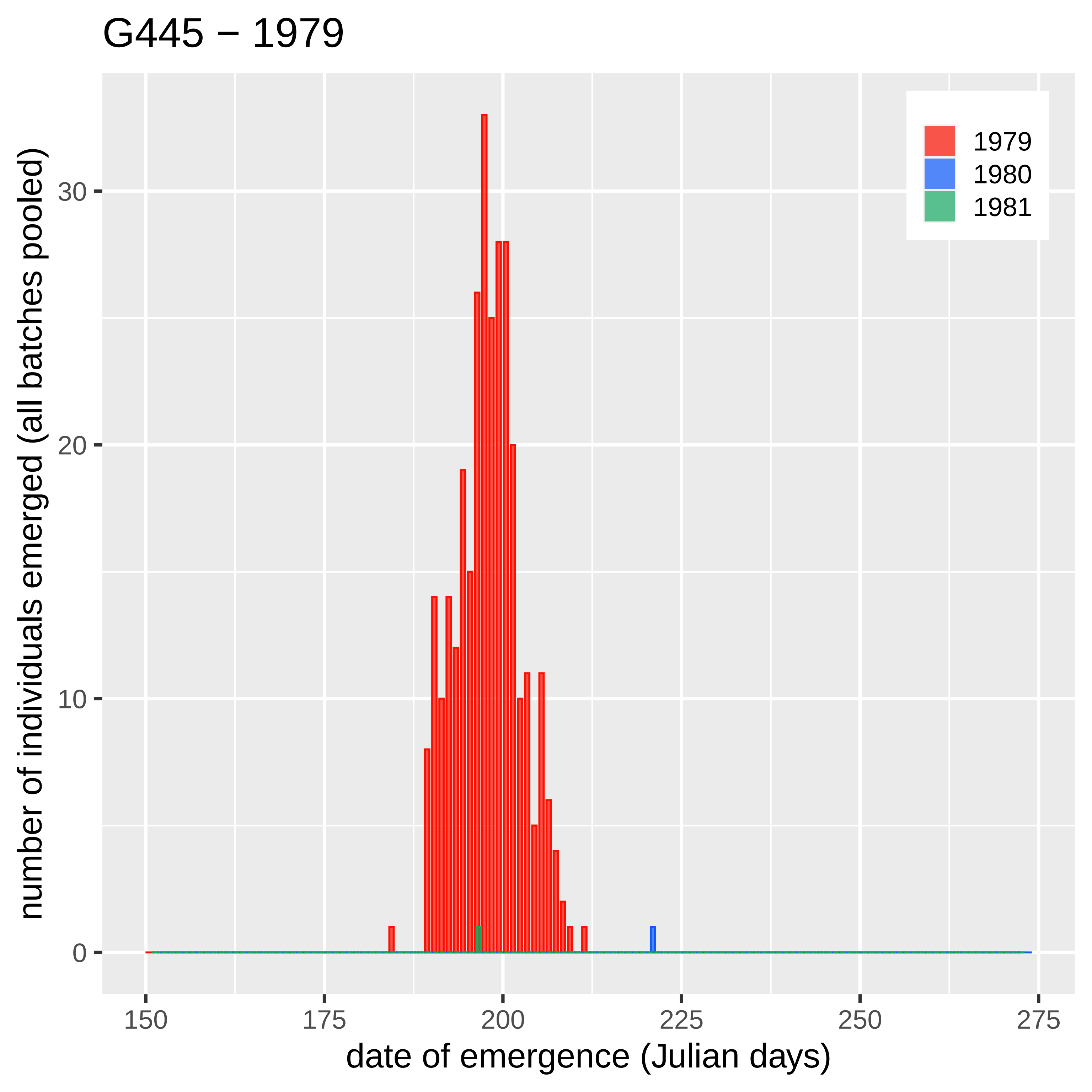
G445 − 1979
1979
1980
1981
0
10
20
30
150
175
200
225
250
275
date of emergence (Julian days)
number of individuals emerged (all batches pooled)

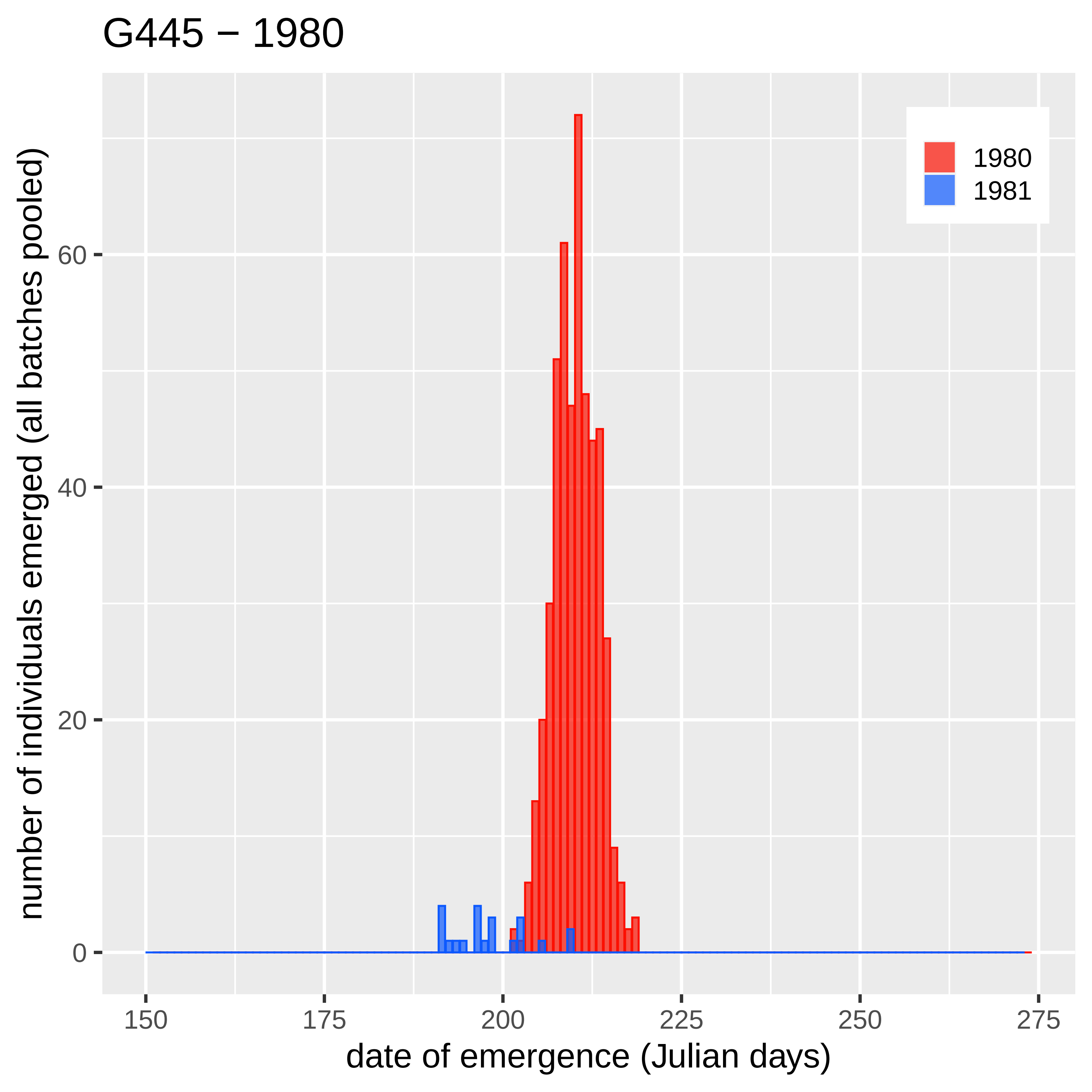
G445 − 1980
1980
1981
number of individuals emerged (all batches pooled)
0
20
40
60
150
175
200
225
250
275
date of emergence (Julian days)

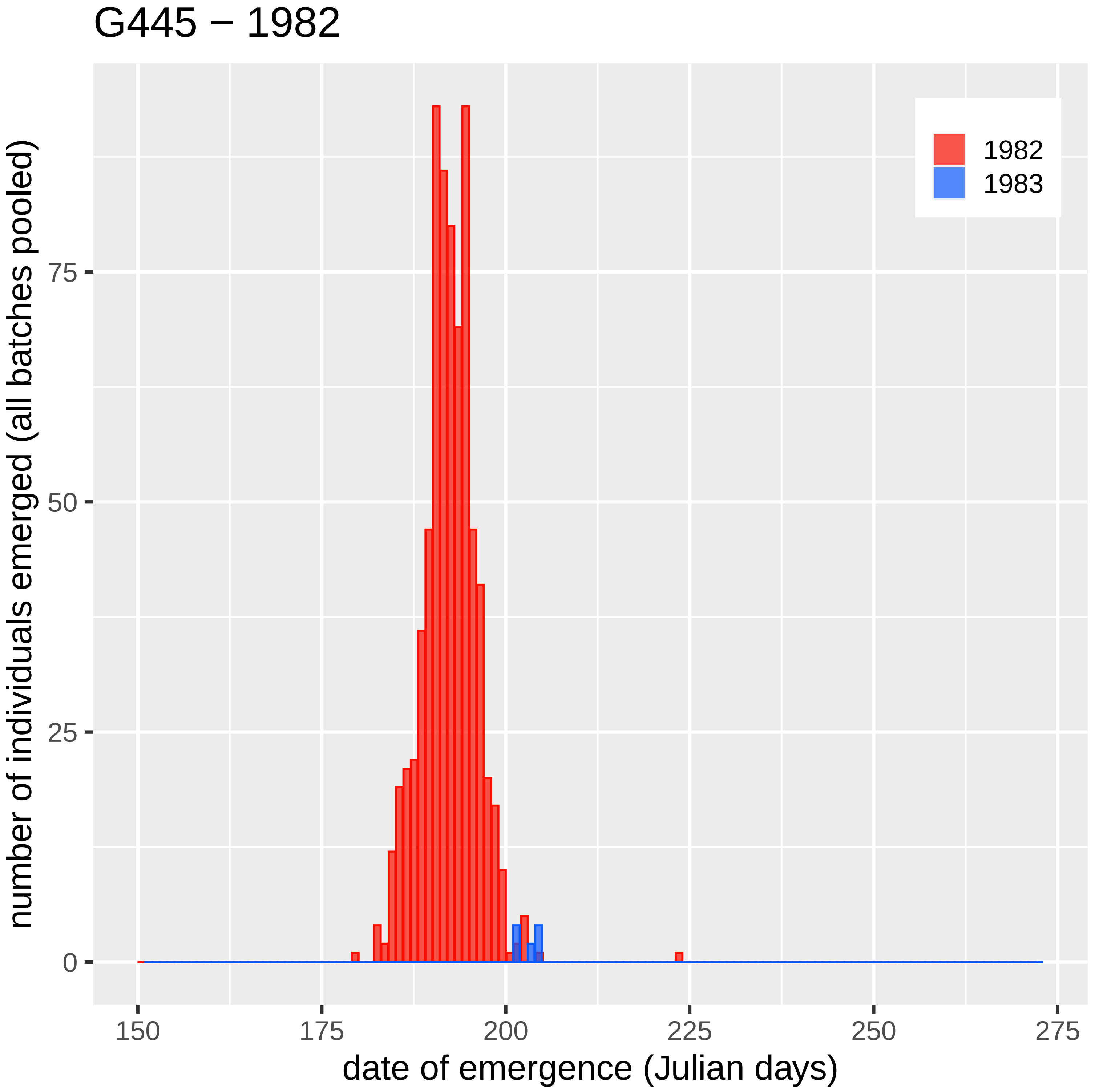
G445 − 1982
1982
1983
0
25
50
75
150
175
200
225
250
275
number of individuals emerged (all batches pooled)
date of emergence (Julian days)

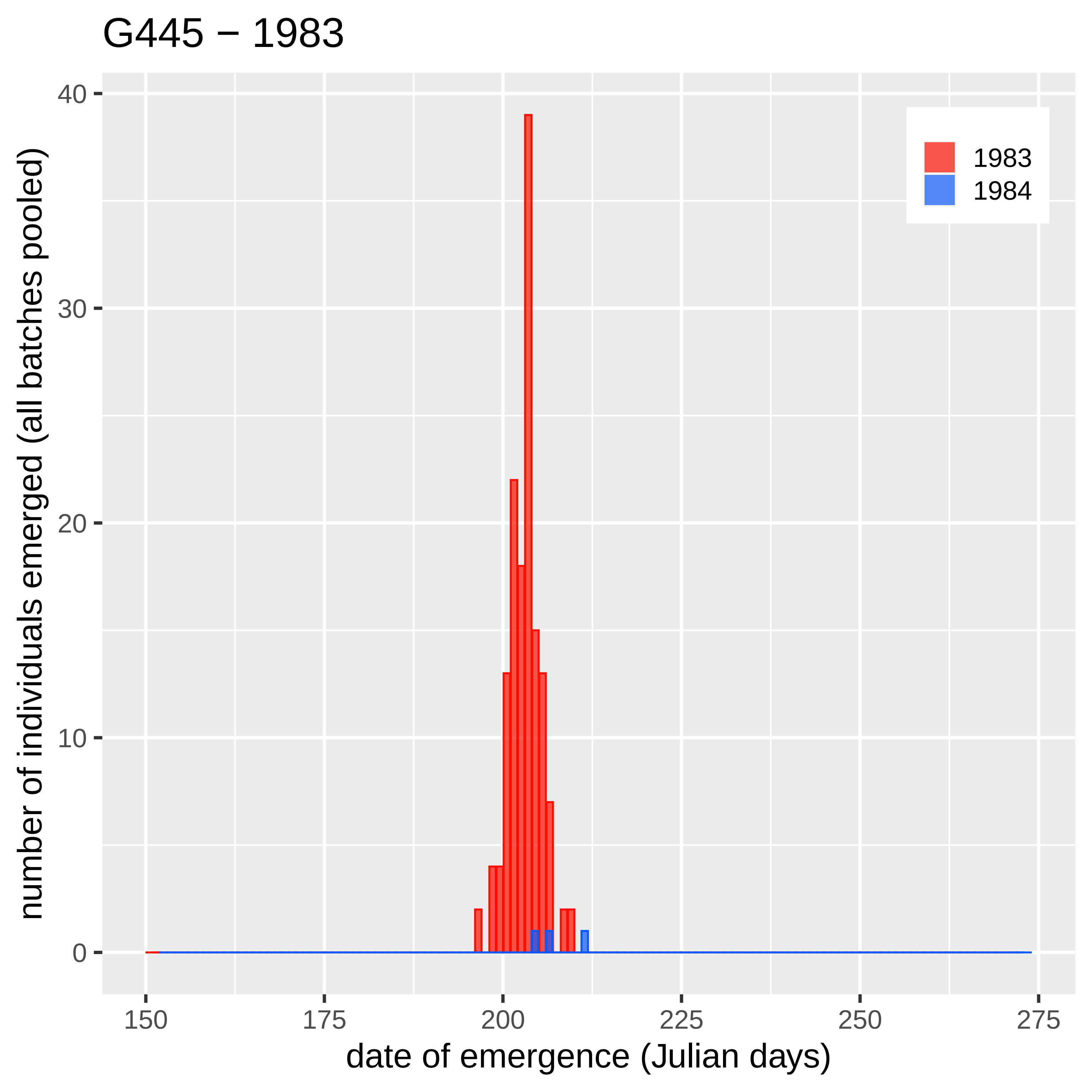
G445 − 1983
number of individuals emerged (all batches pooled)
0
10
20
30
40
150
175
200
225
250
275
date of emergence (Julian days)
1983
1984

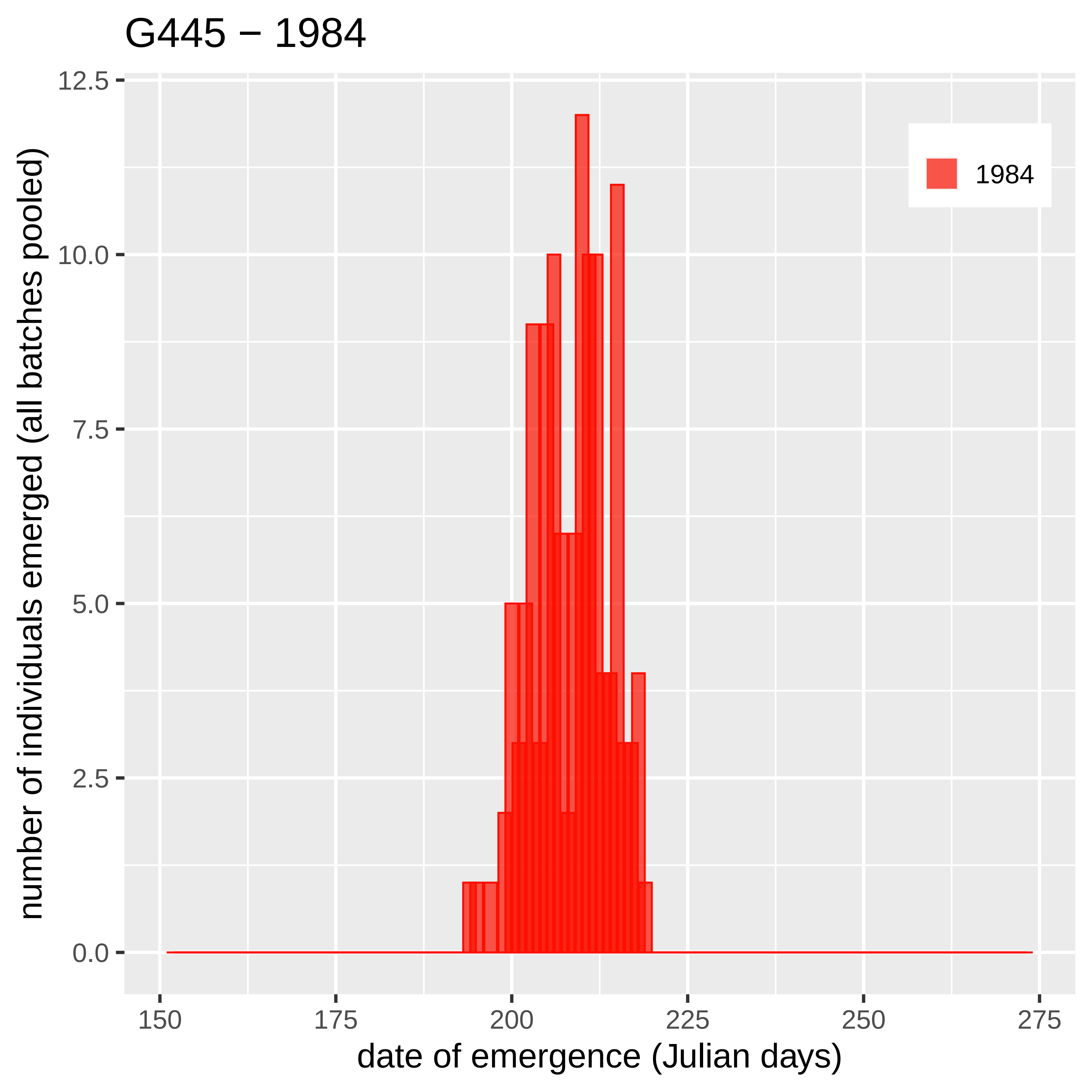

G445 − 1984
12.5
10.0
7.5
5.0
2.5
0.0
150
175
200
225
250
275
date of emergence (Julian days)
number of individuals emerged (all batches pooled)
1984
