# Supplementary figures and images for: Monitoring of adult emergence in the pine processionary moth between 1970 and 1984 in Mont Ventoux, France
Source: Biodivers Data J. 2021 Feb 17;9:e61086. doi: 10.3897/BDJ.9.e61086 (PMC7904747; doi:10.3897/BDJ.9.e61086)

# C671 – 1970

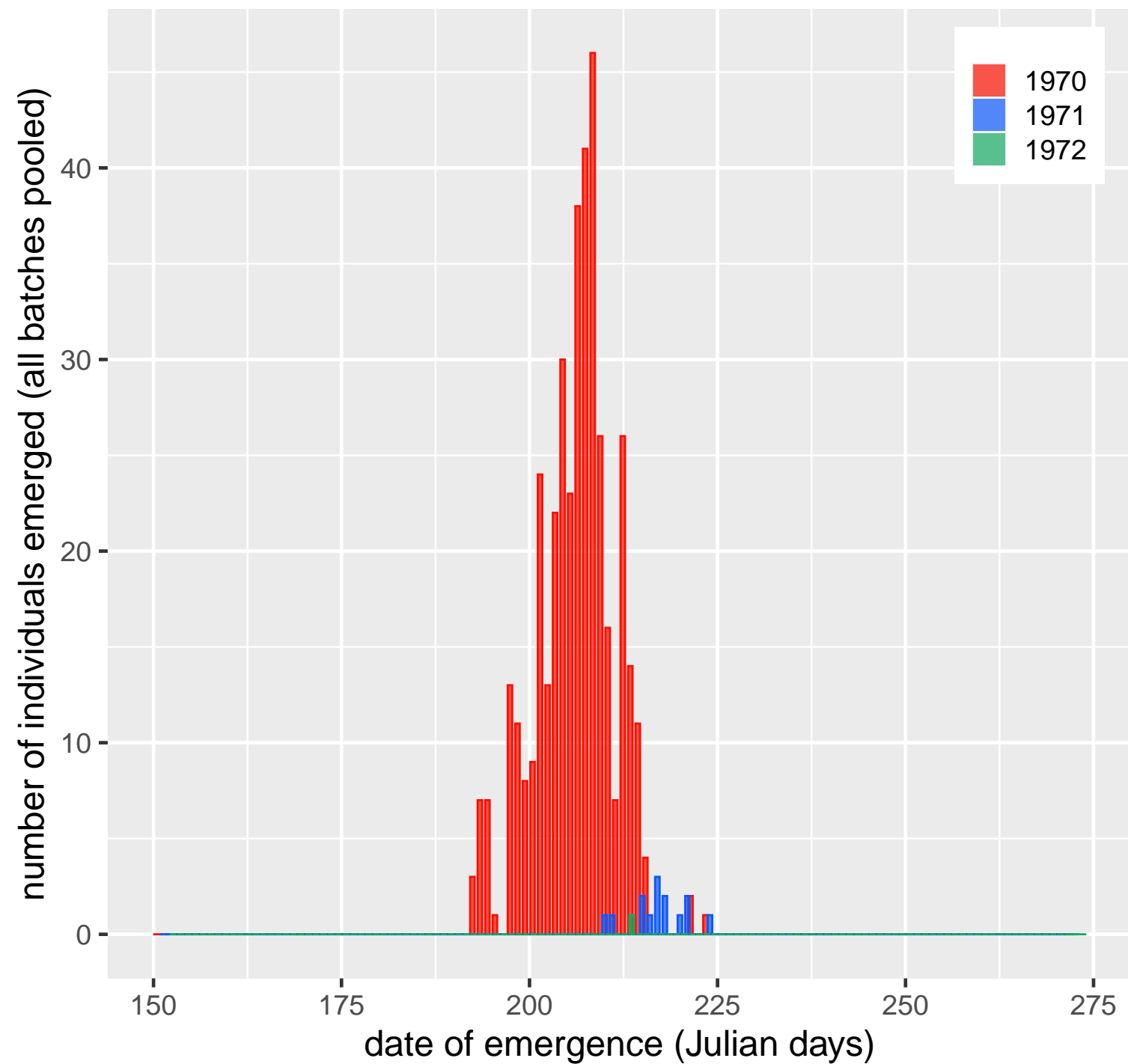

# C671 – 1971

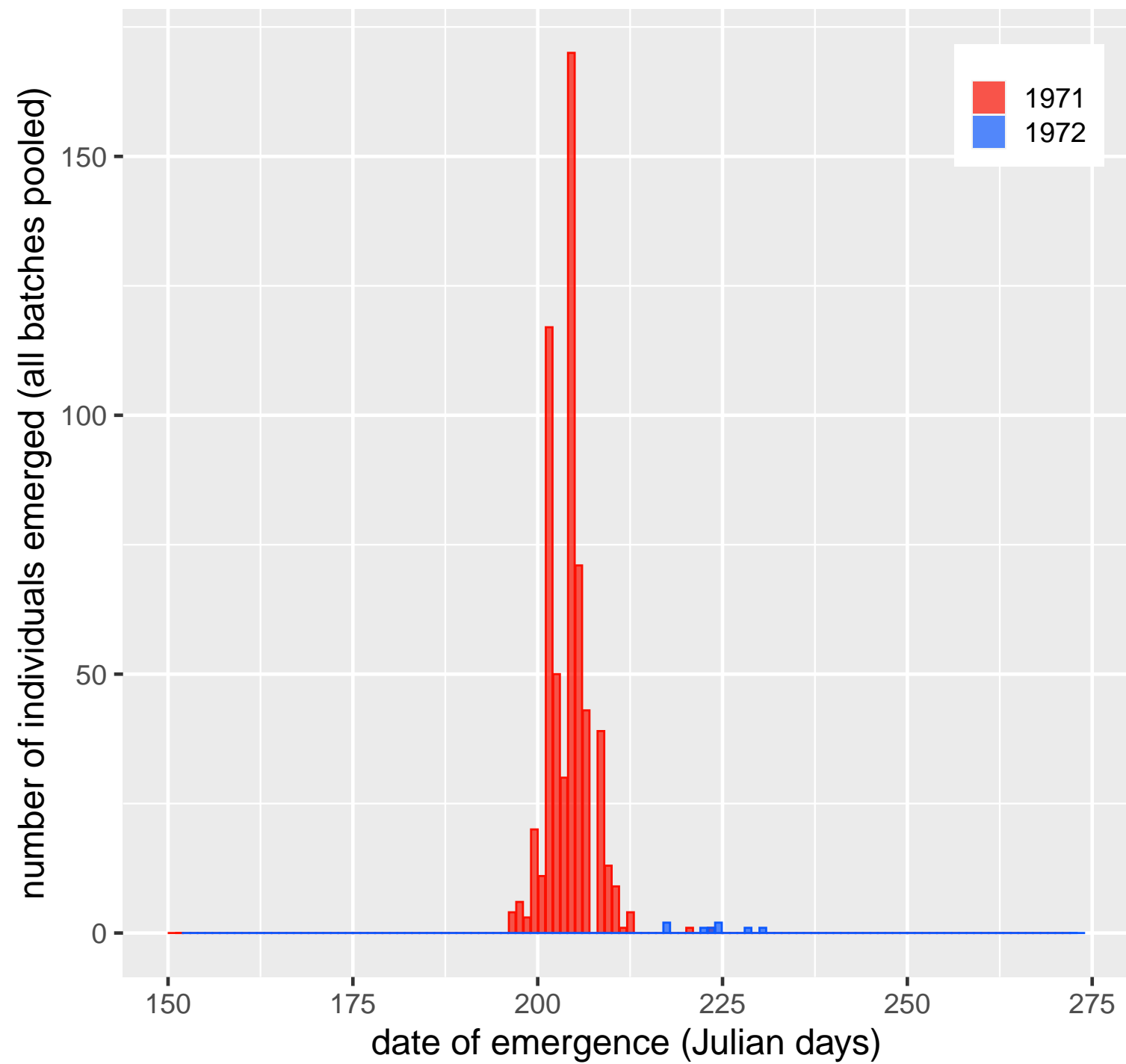

# C671 – 1972

number of individuals emerged (all batches pooled)

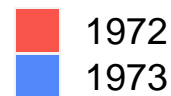

75  
50  
25  
0

150

175

200

225

250

275

date of emergence (Julian days)

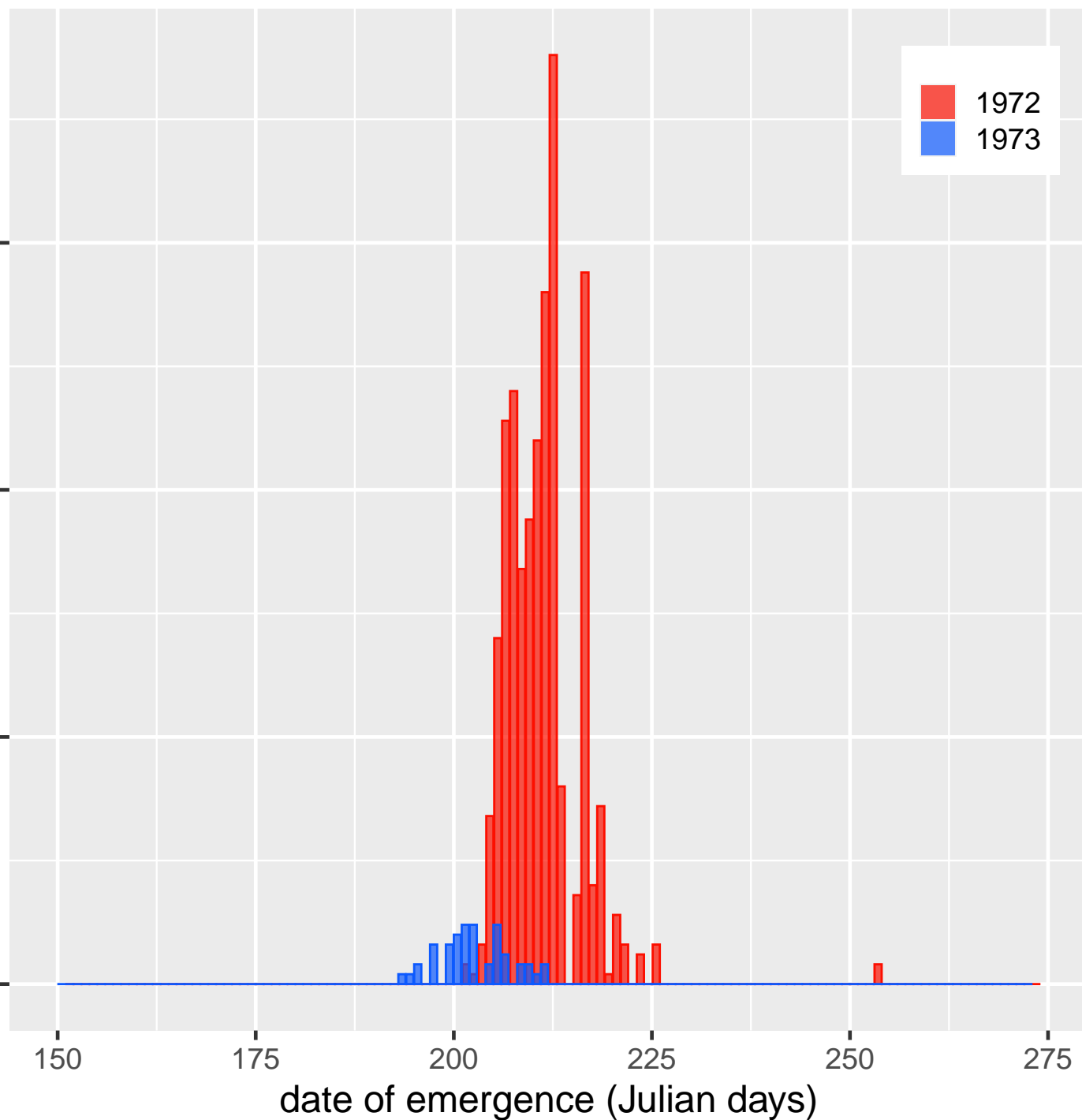

# C671 – 1974

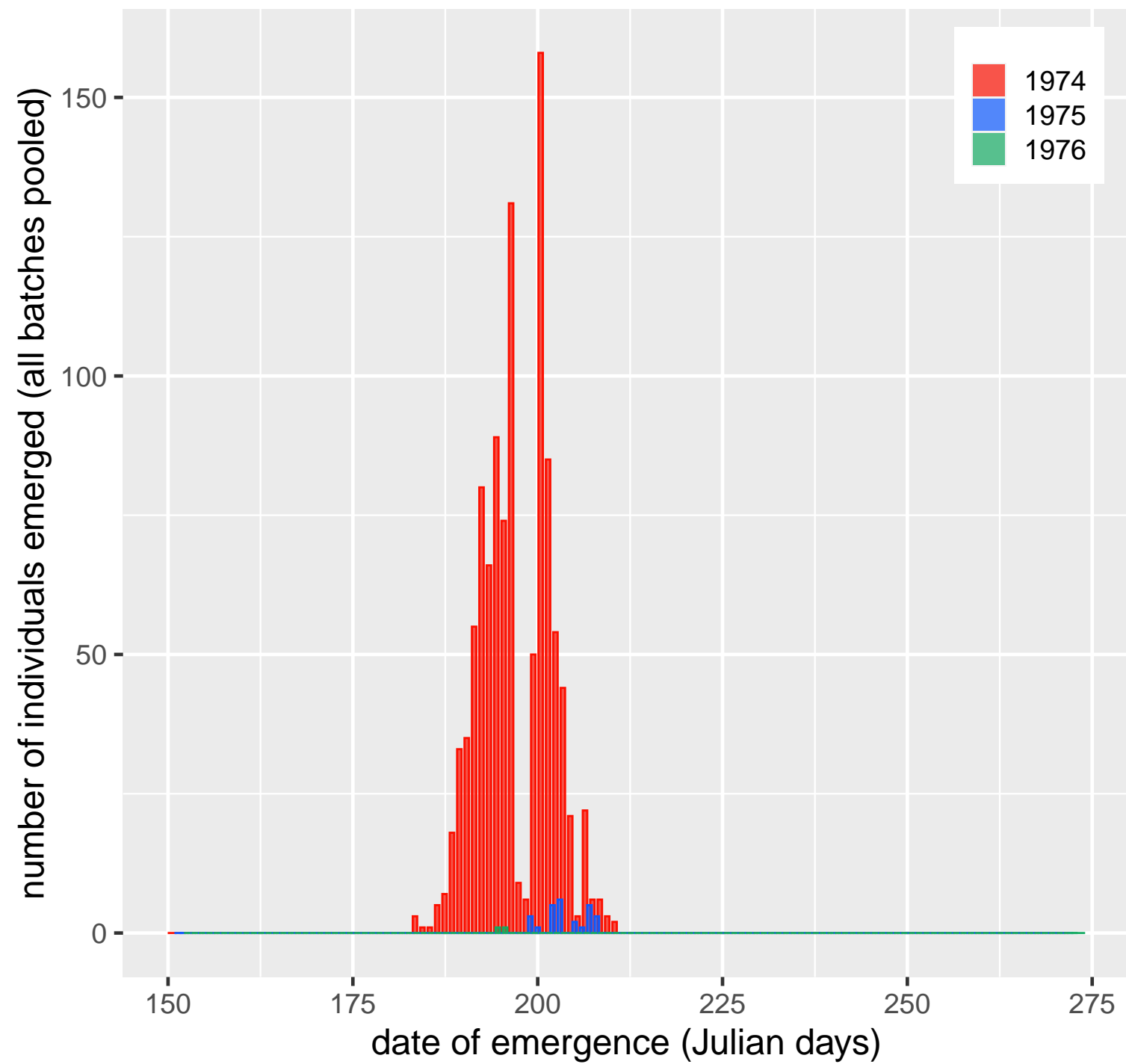

# C671 – 1975

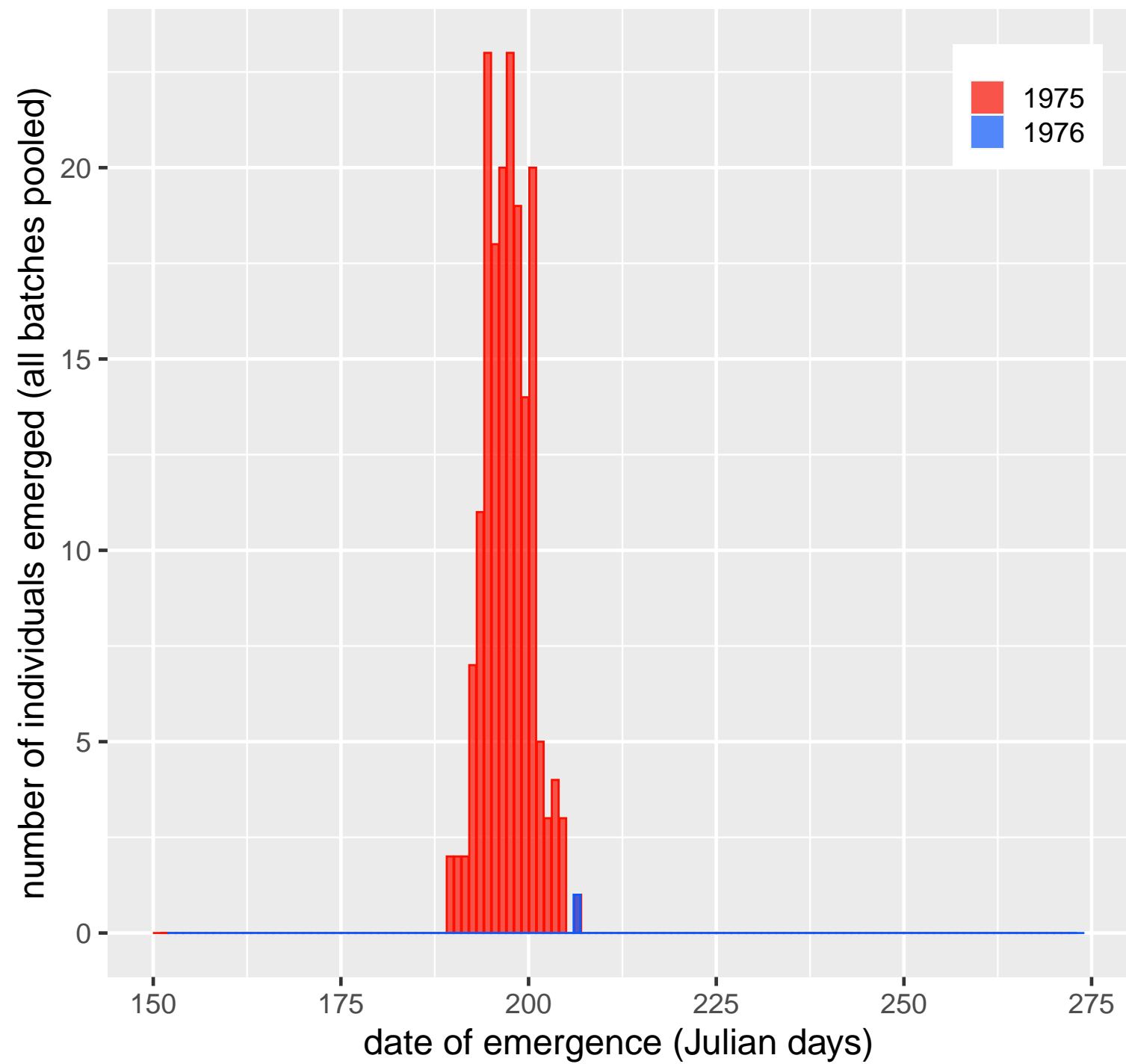

# C671 – 1976

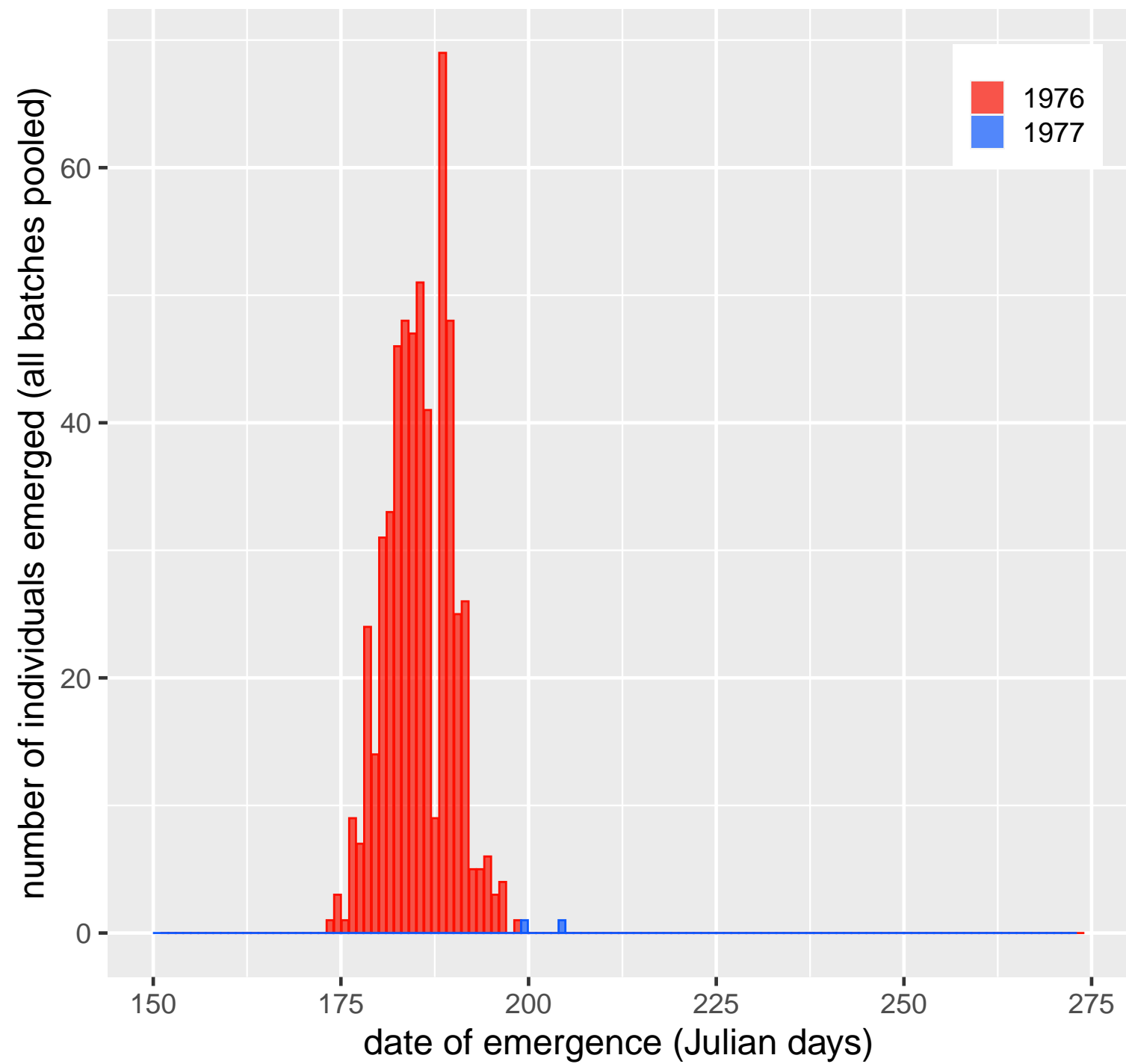

# C671 – 1977

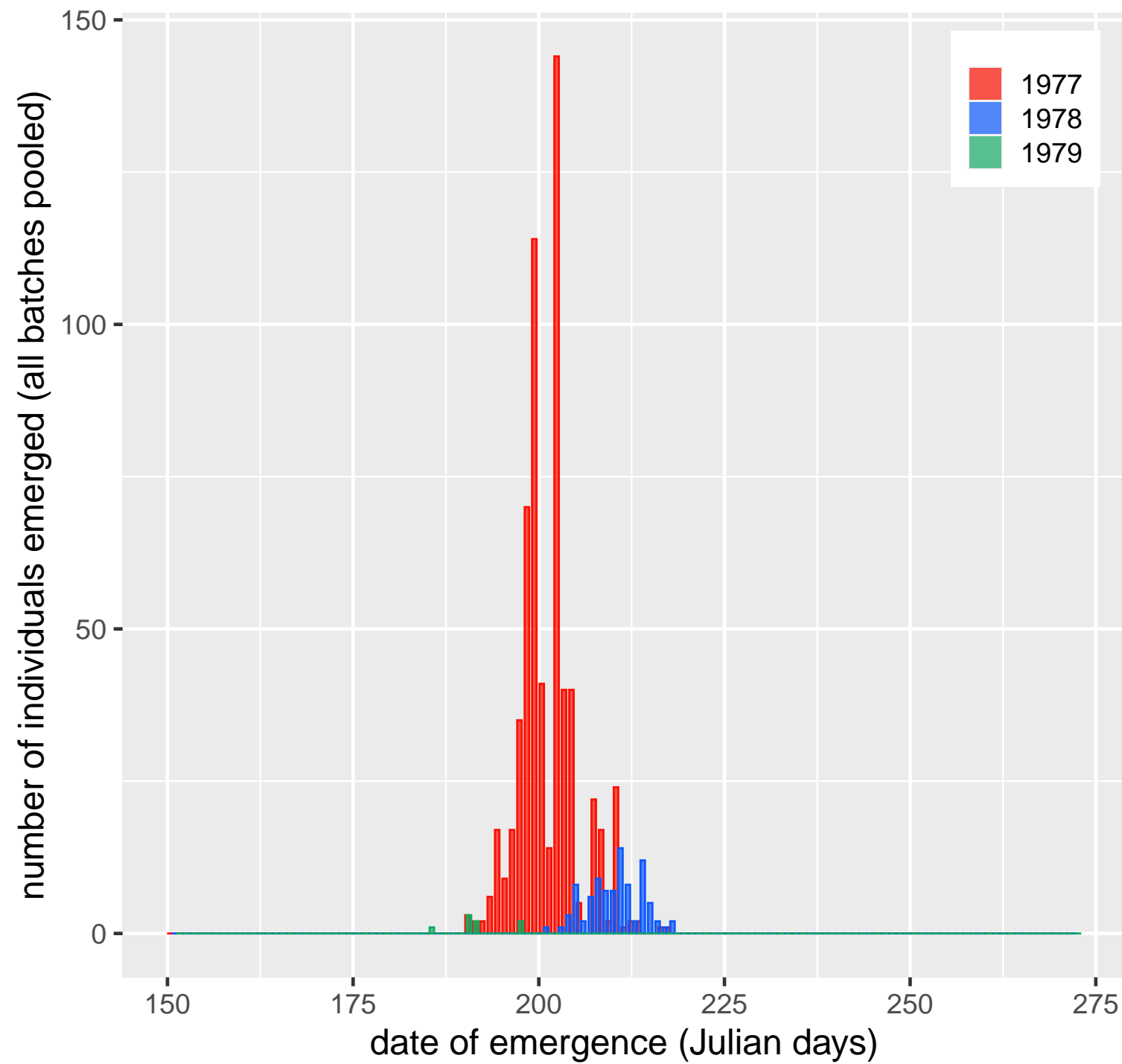

# C671 – 1978

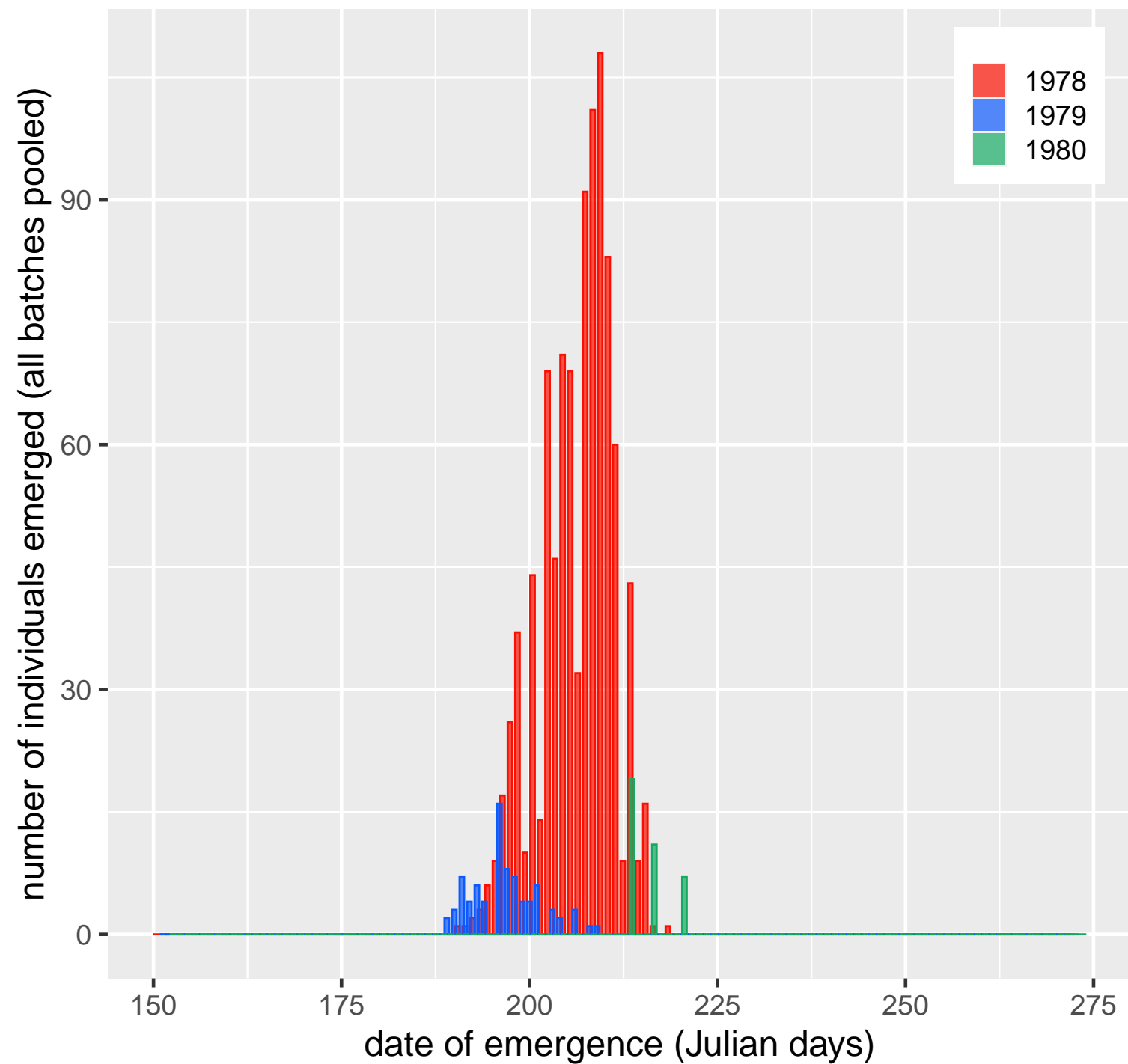

# C671 – 1980

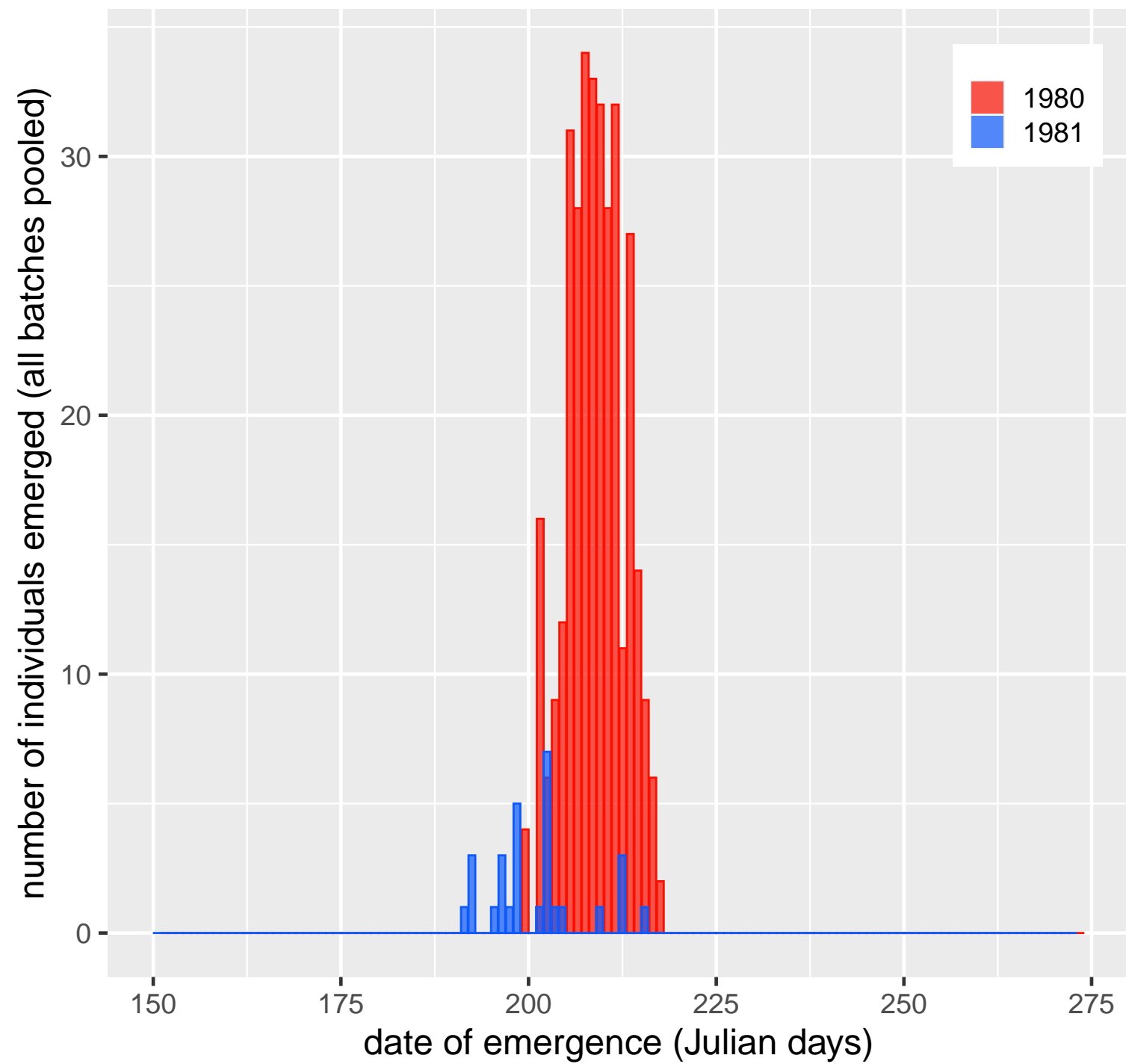

# C671 – 1981

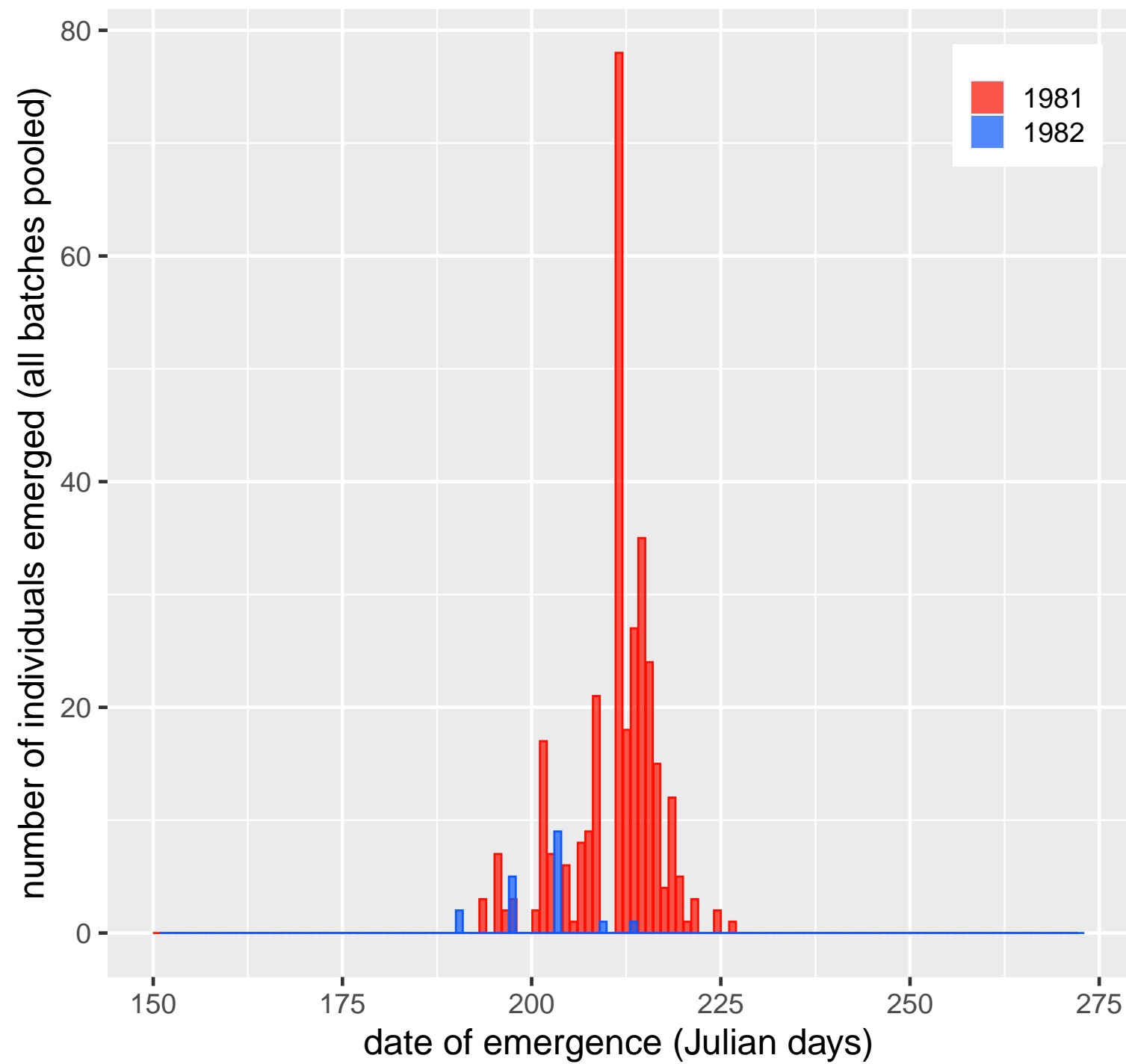

# C671 – 1982

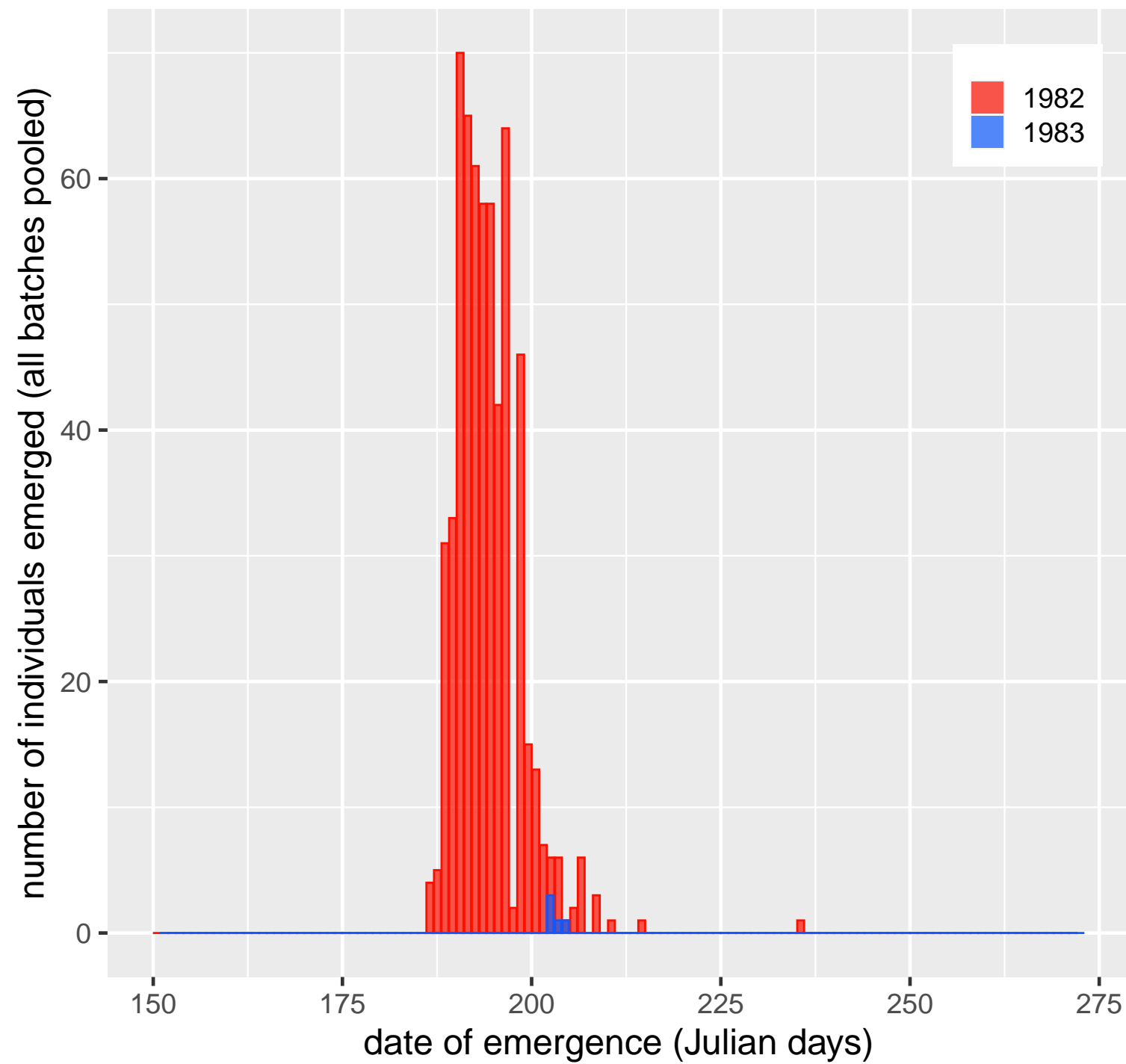

# C671 – 1983

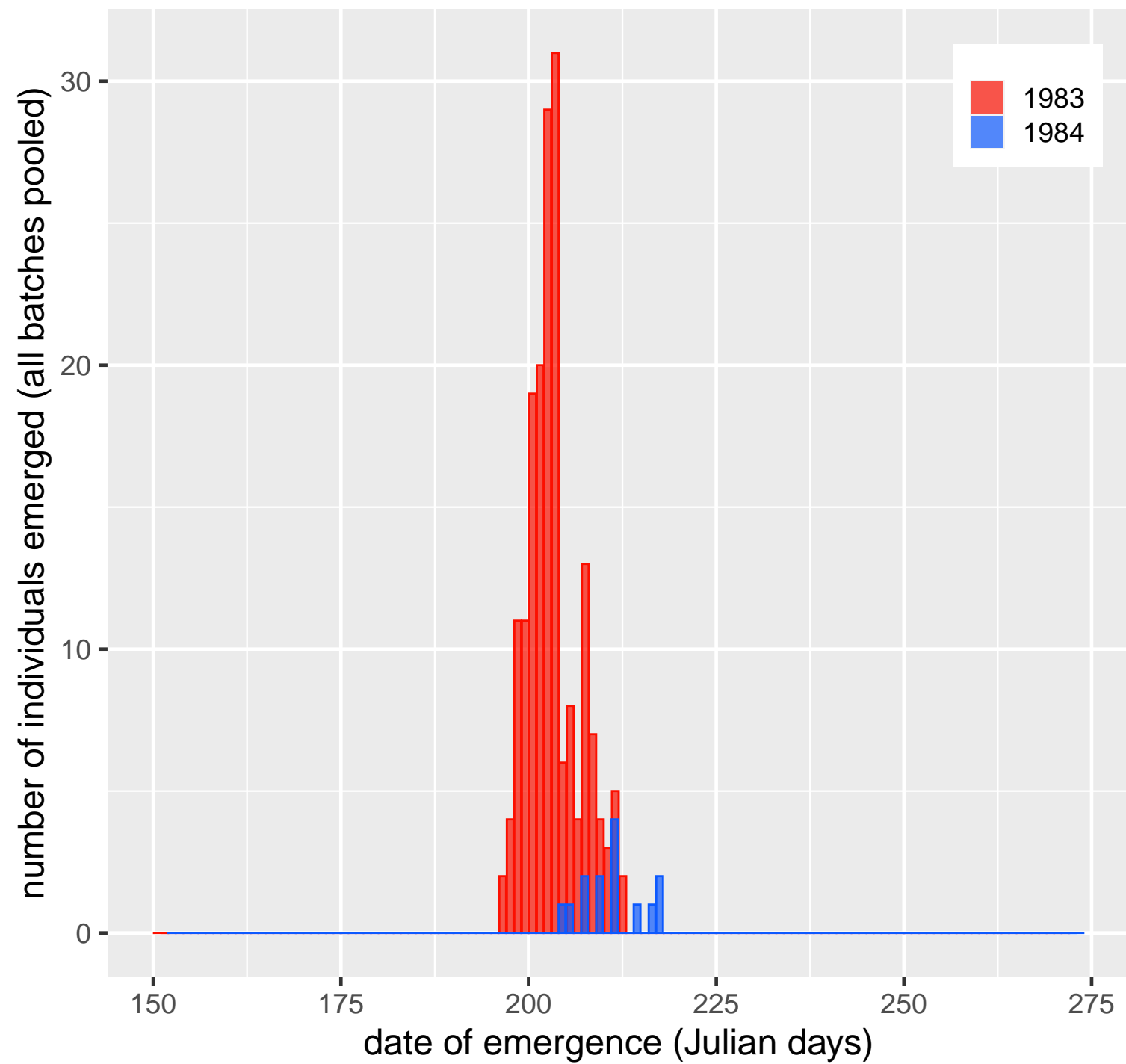

Supplement: Supplementary material 2 — Emergence curves for each cohort sampled in site C671 [file bdj-09-e61086-s002.pdf]

# A688 – 1971

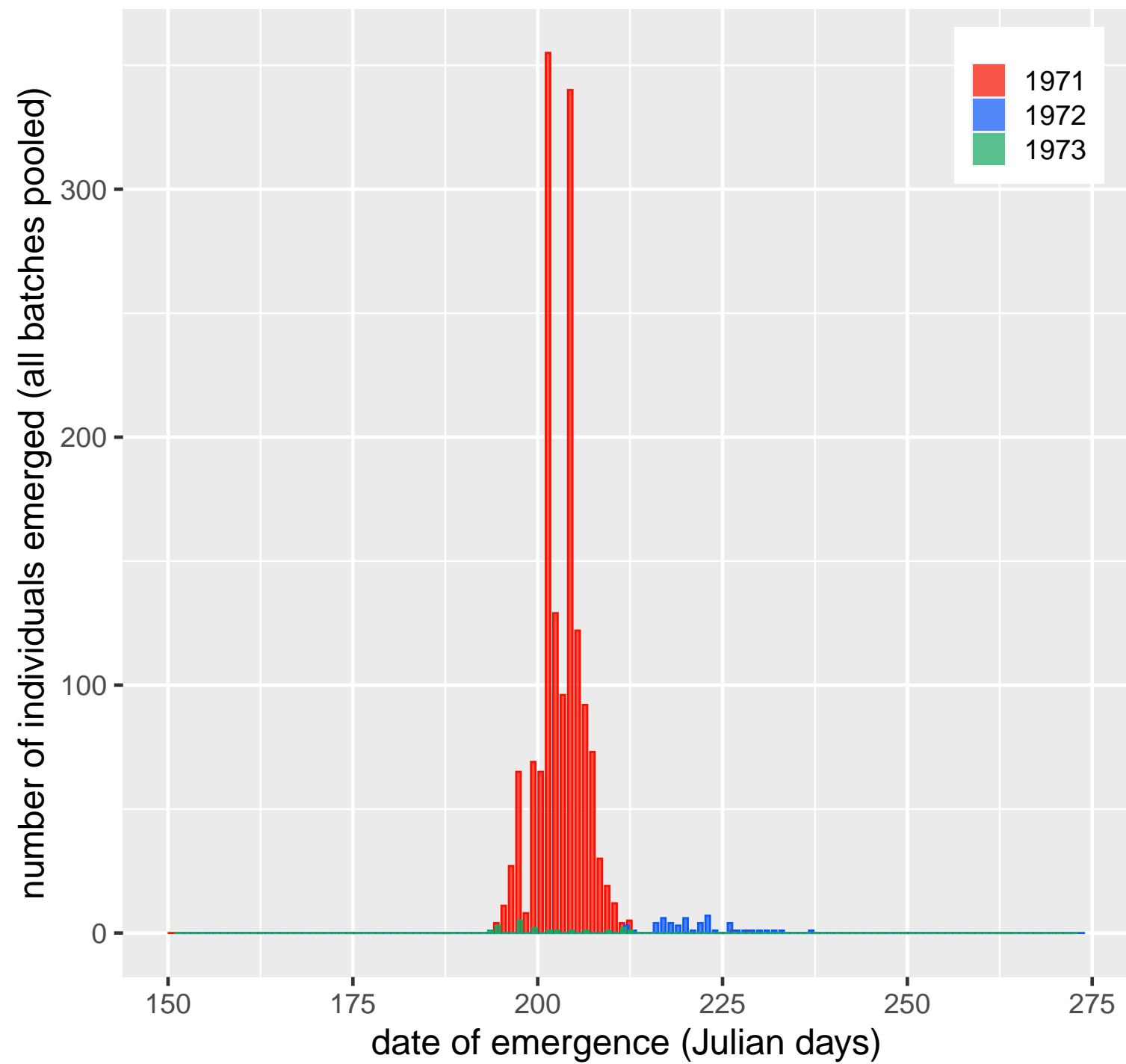

# A688 – 1972

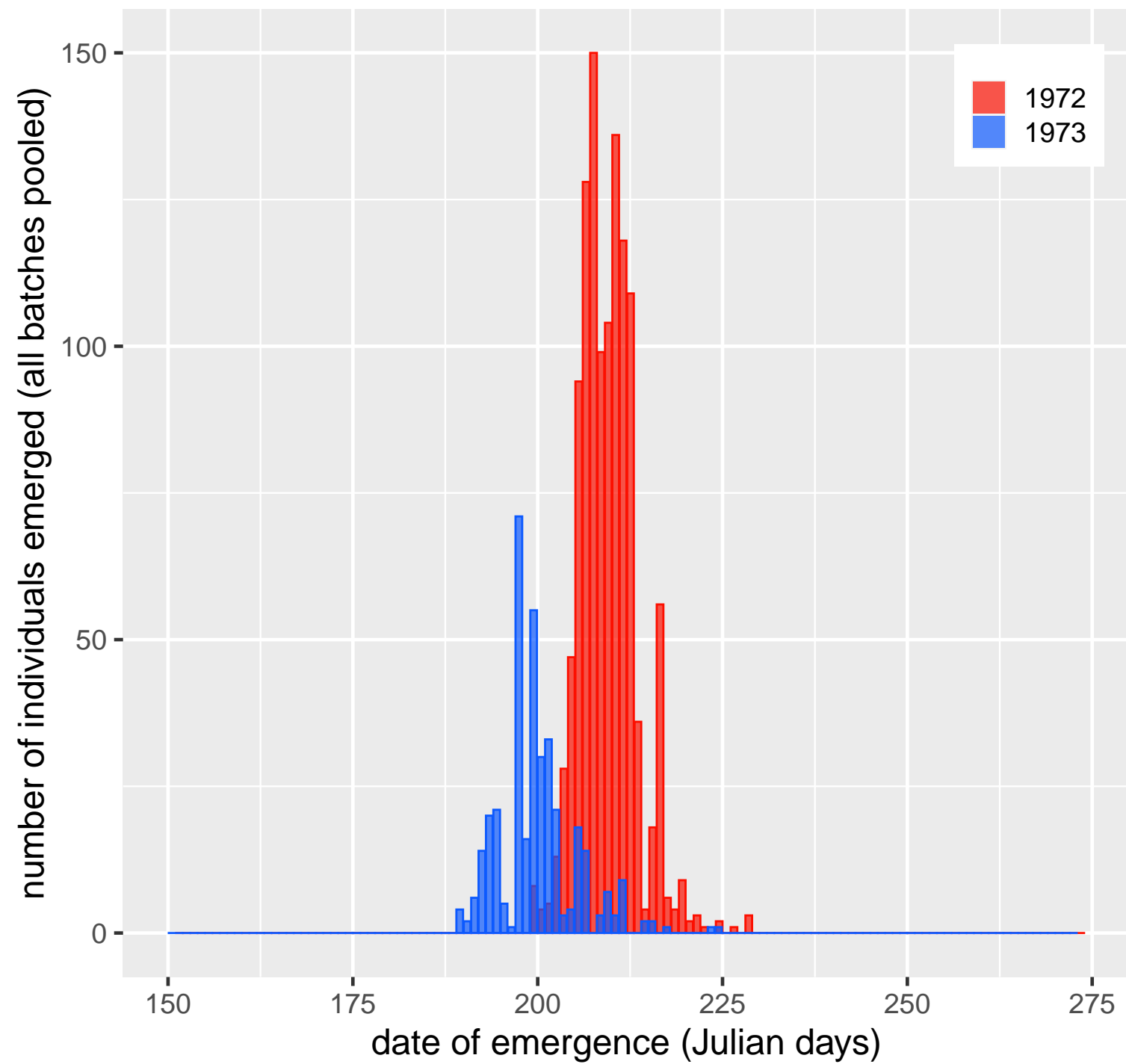

# A688 – 1973

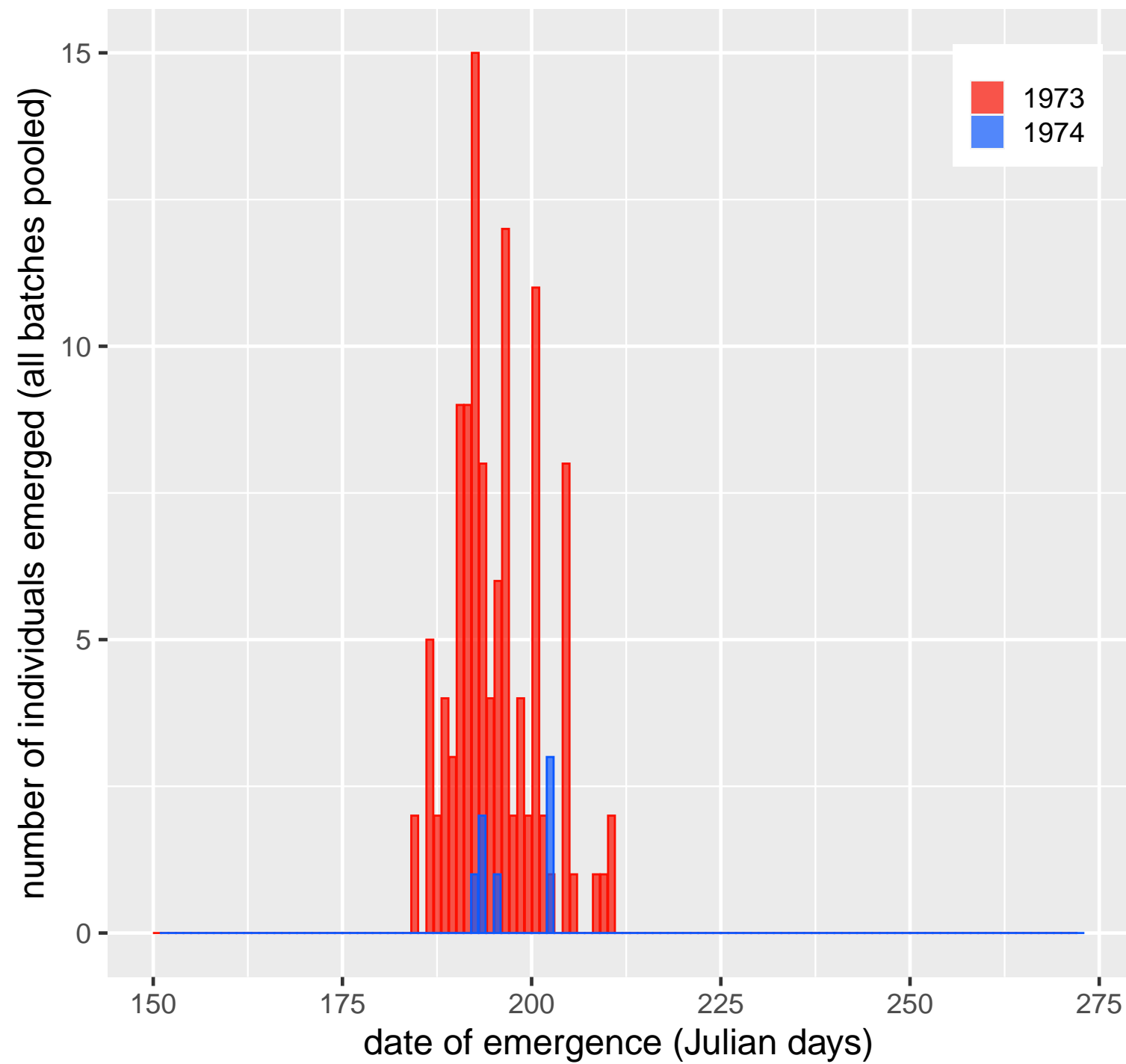

# A688 – 1974

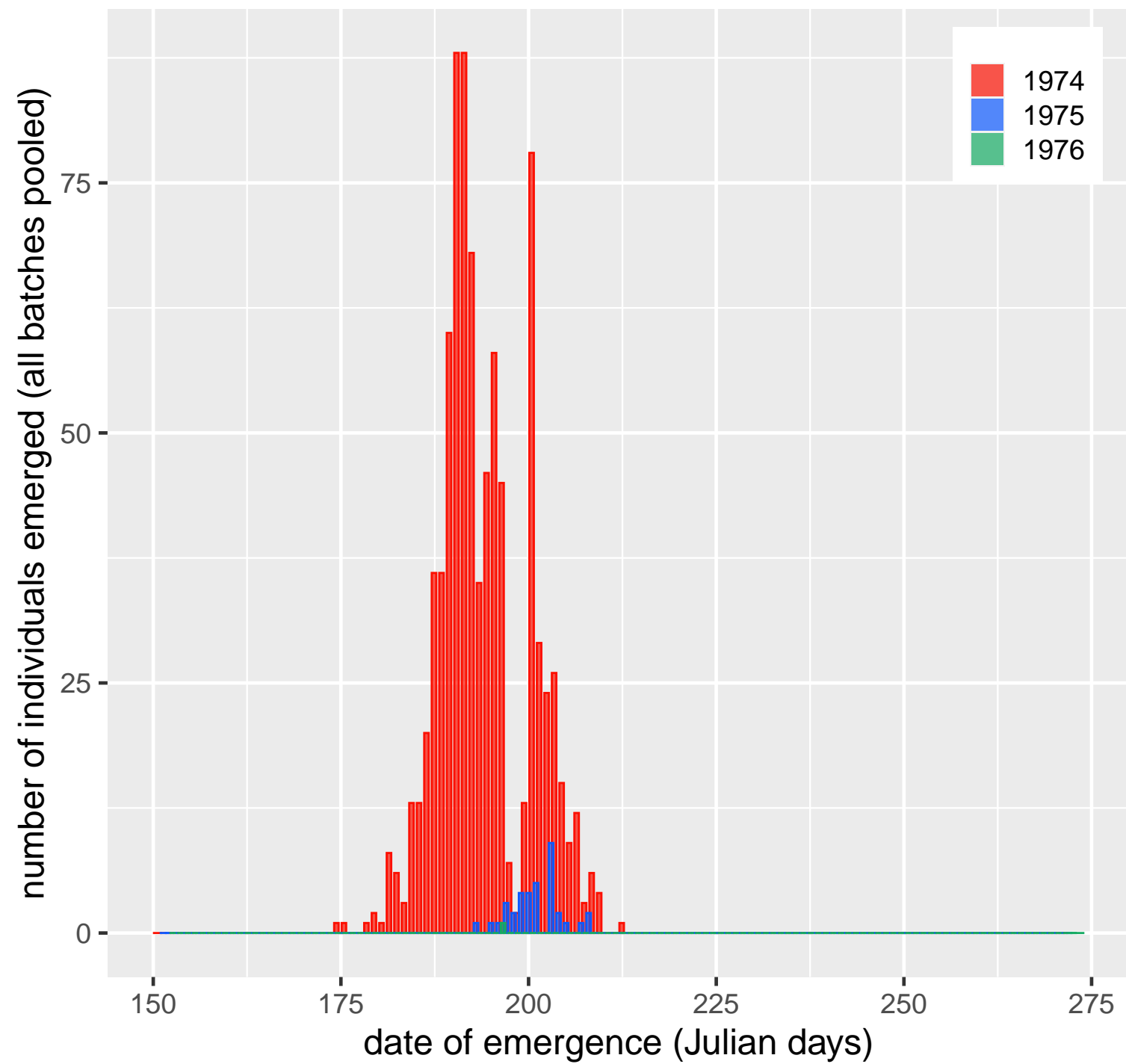

# A688 – 1975

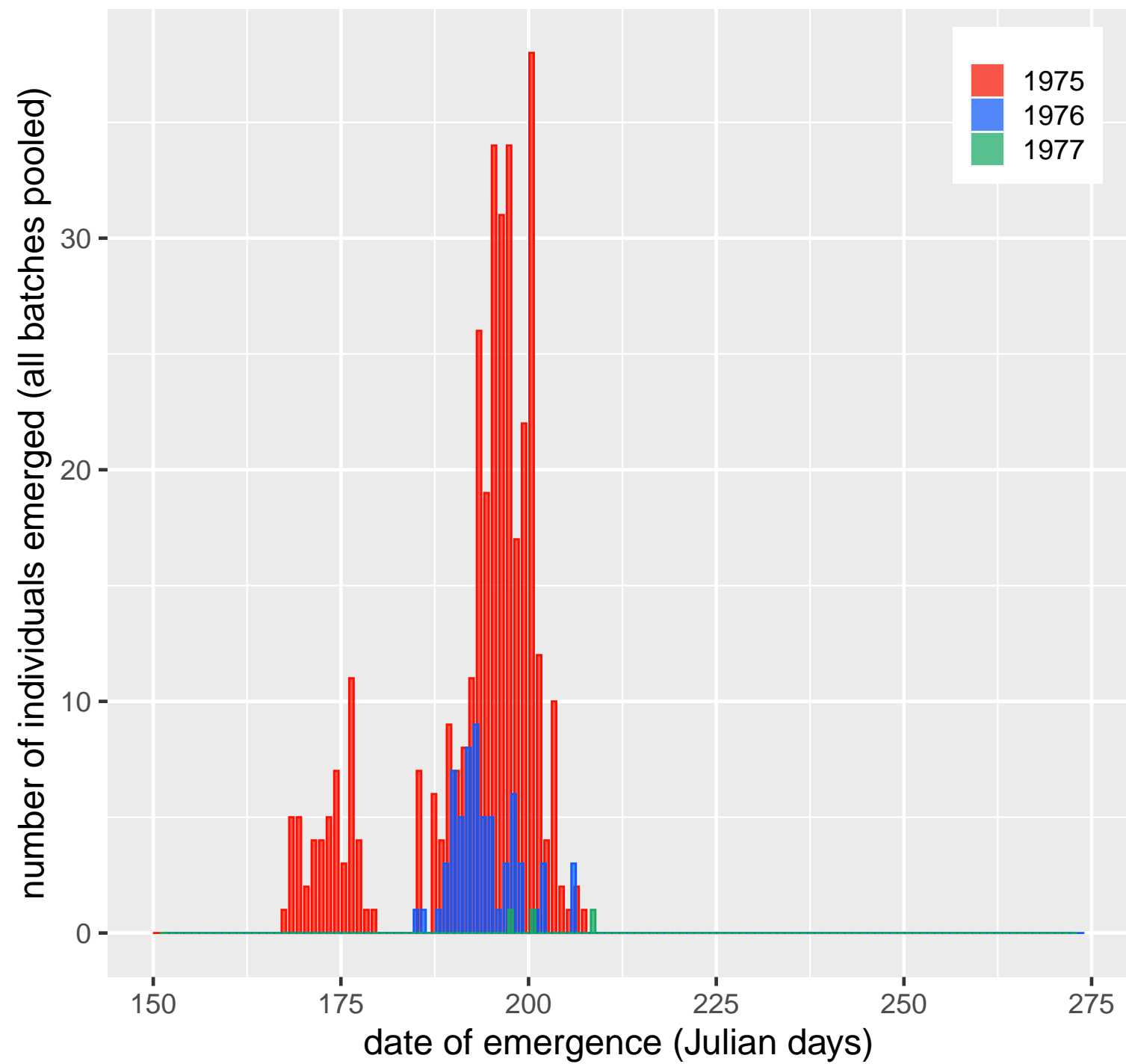



# A688 – 1978

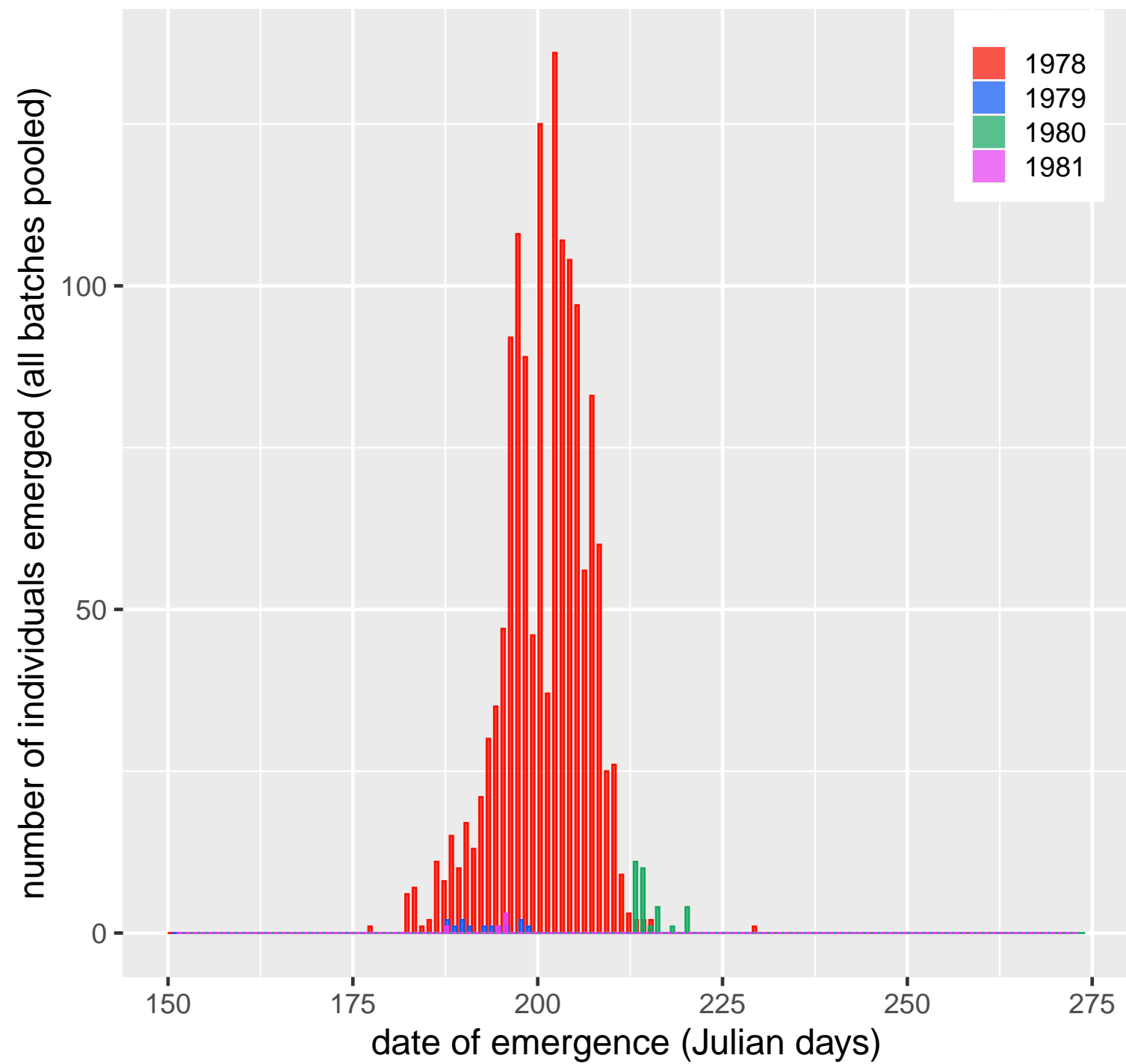

# A688 – 1979

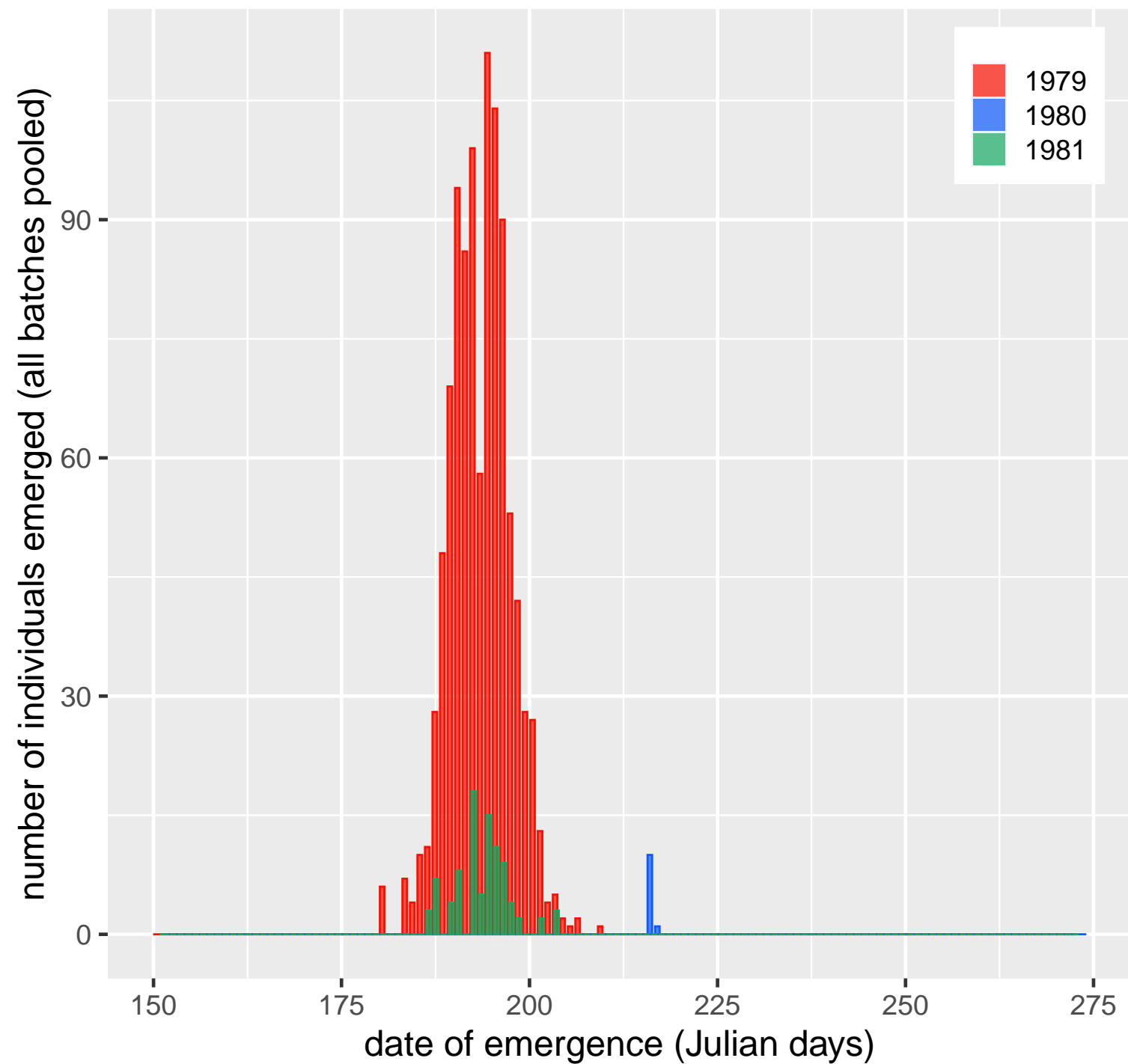

# A688 – 1980

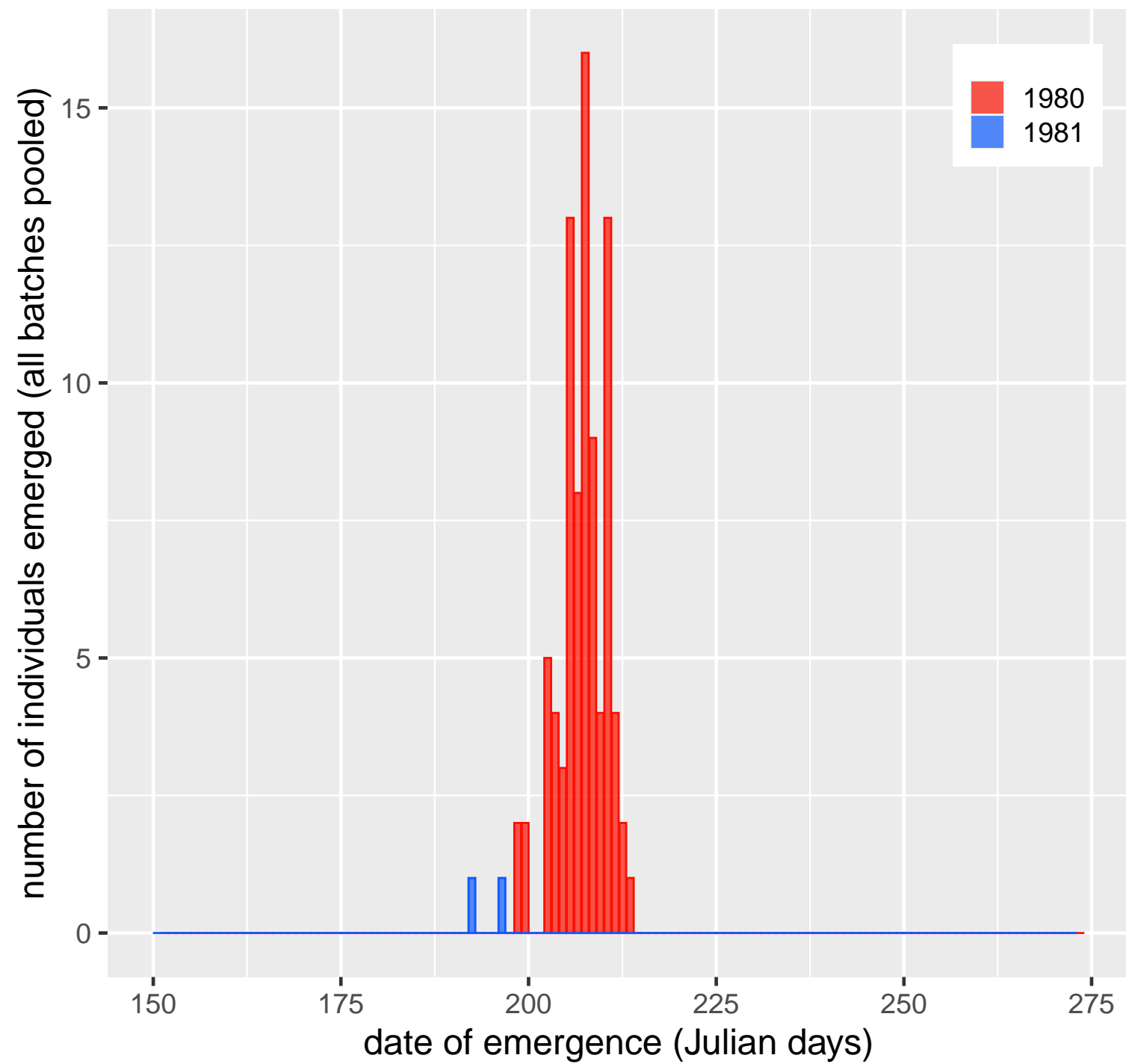

# A688 – 1982

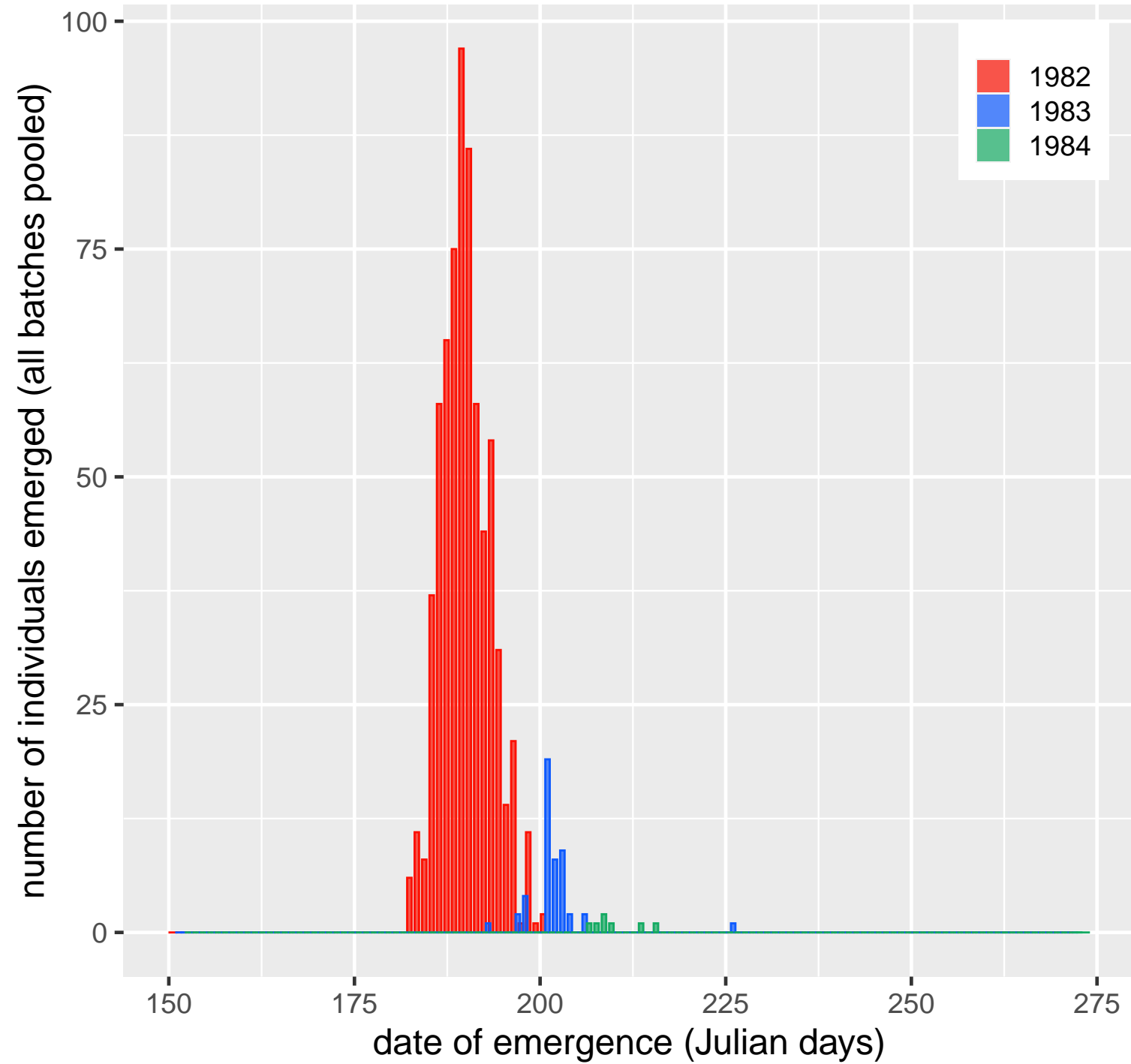

# A688 – 1983

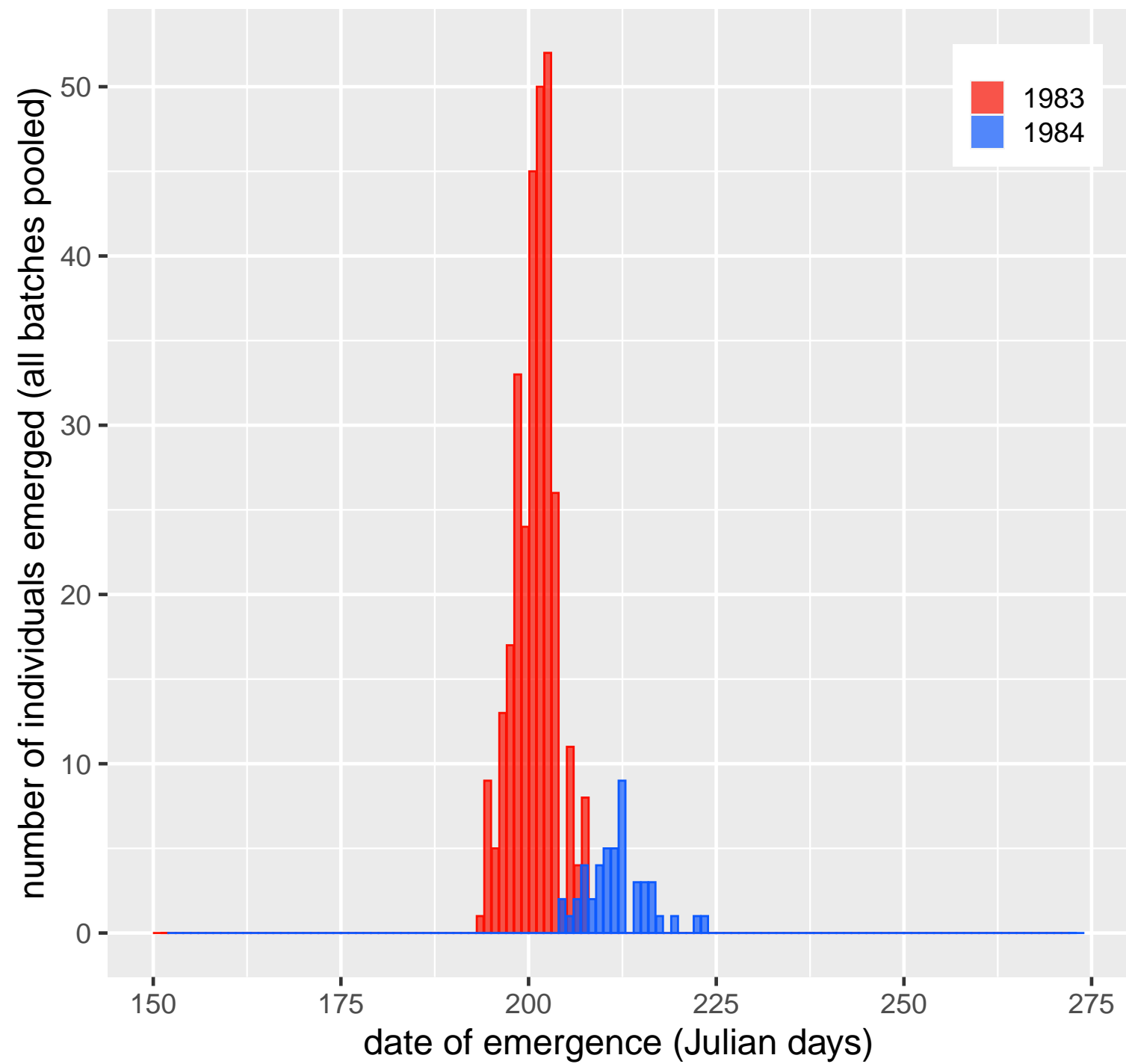

# A688 – 1984

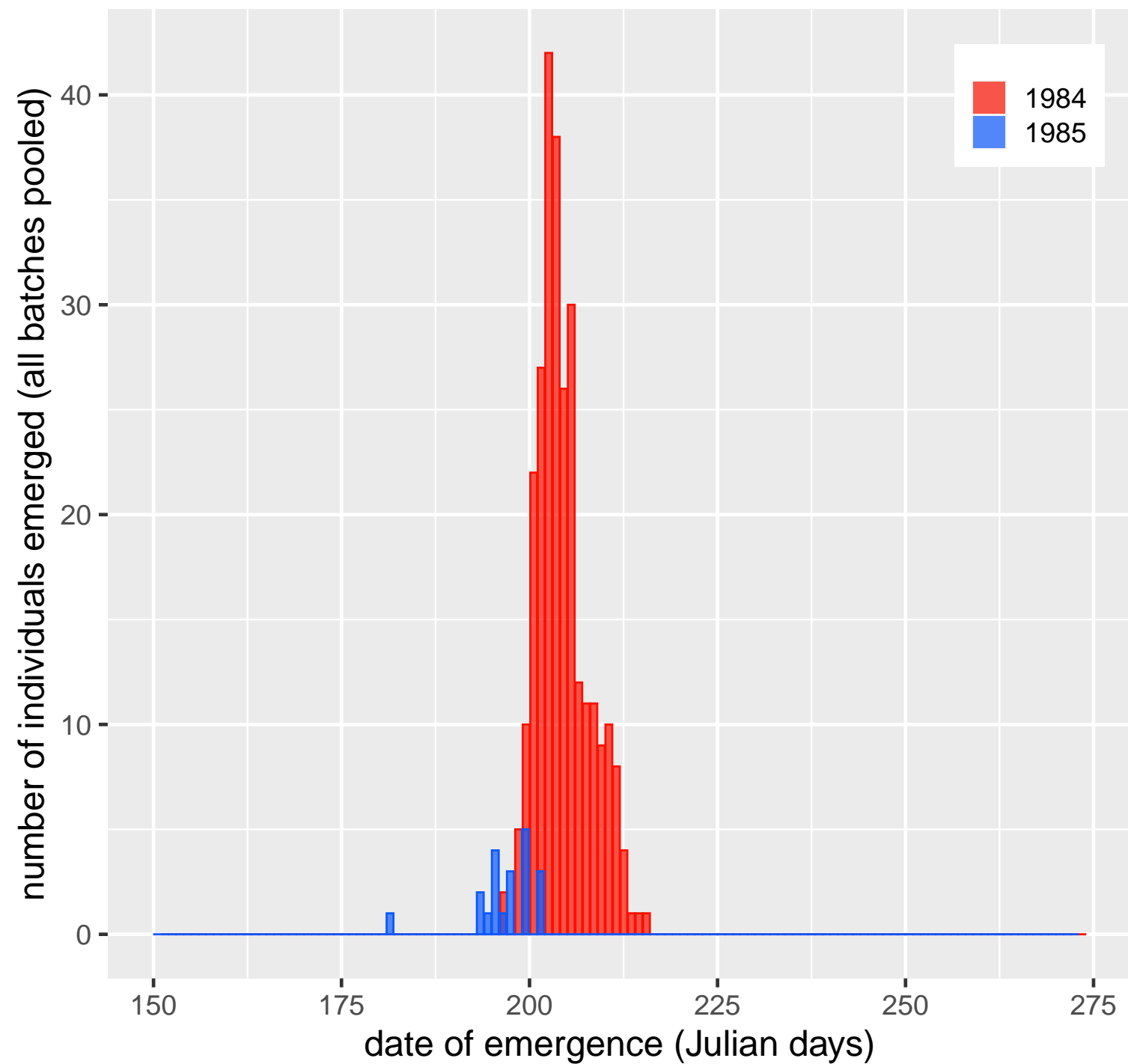

Supplement: Supplementary material 3 — Emergence curves for each cohort sampled in site A688 [file bdj-09-e61086-s003.pdf]

# B697 – 1971

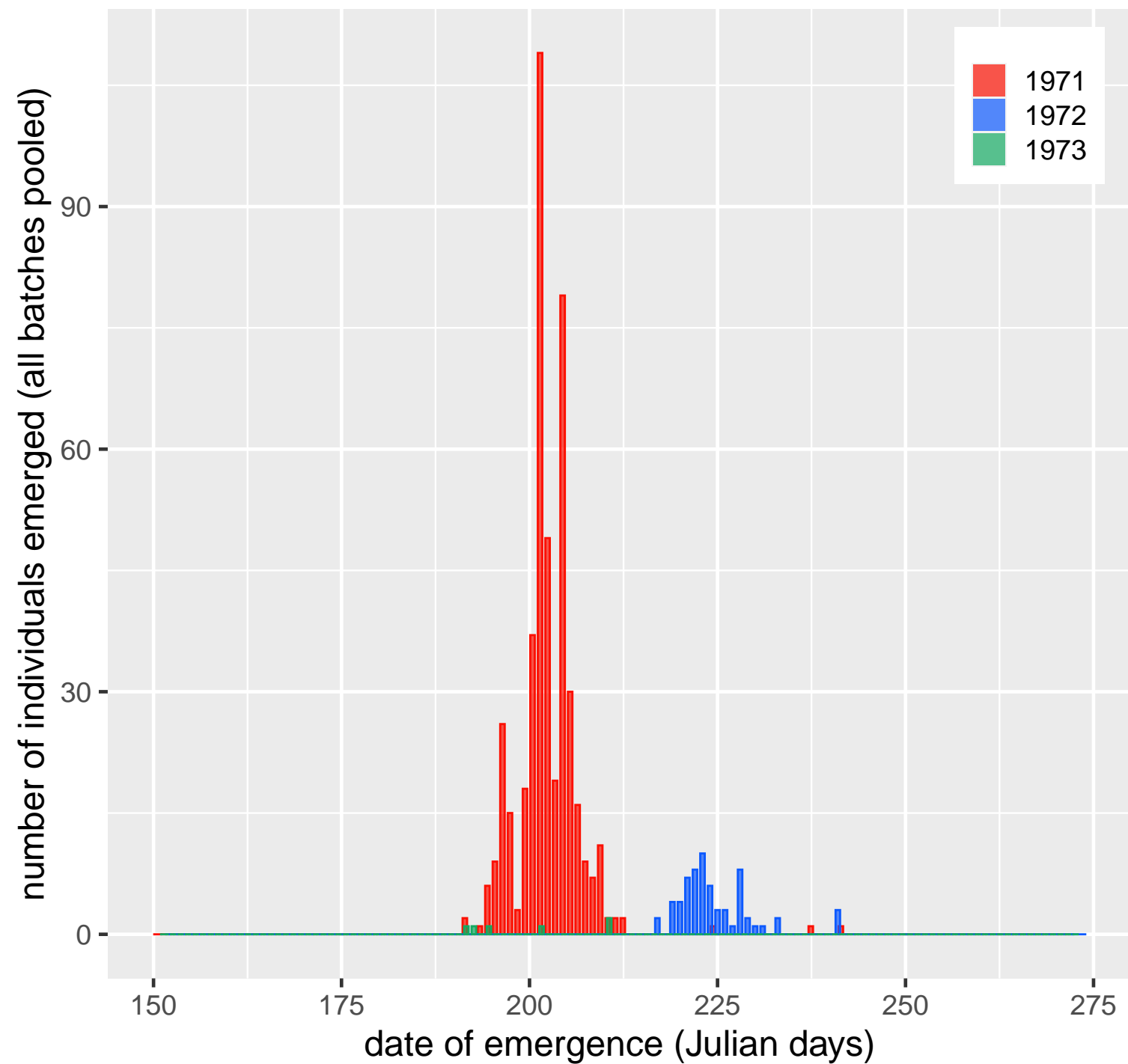



# B697 – 1973

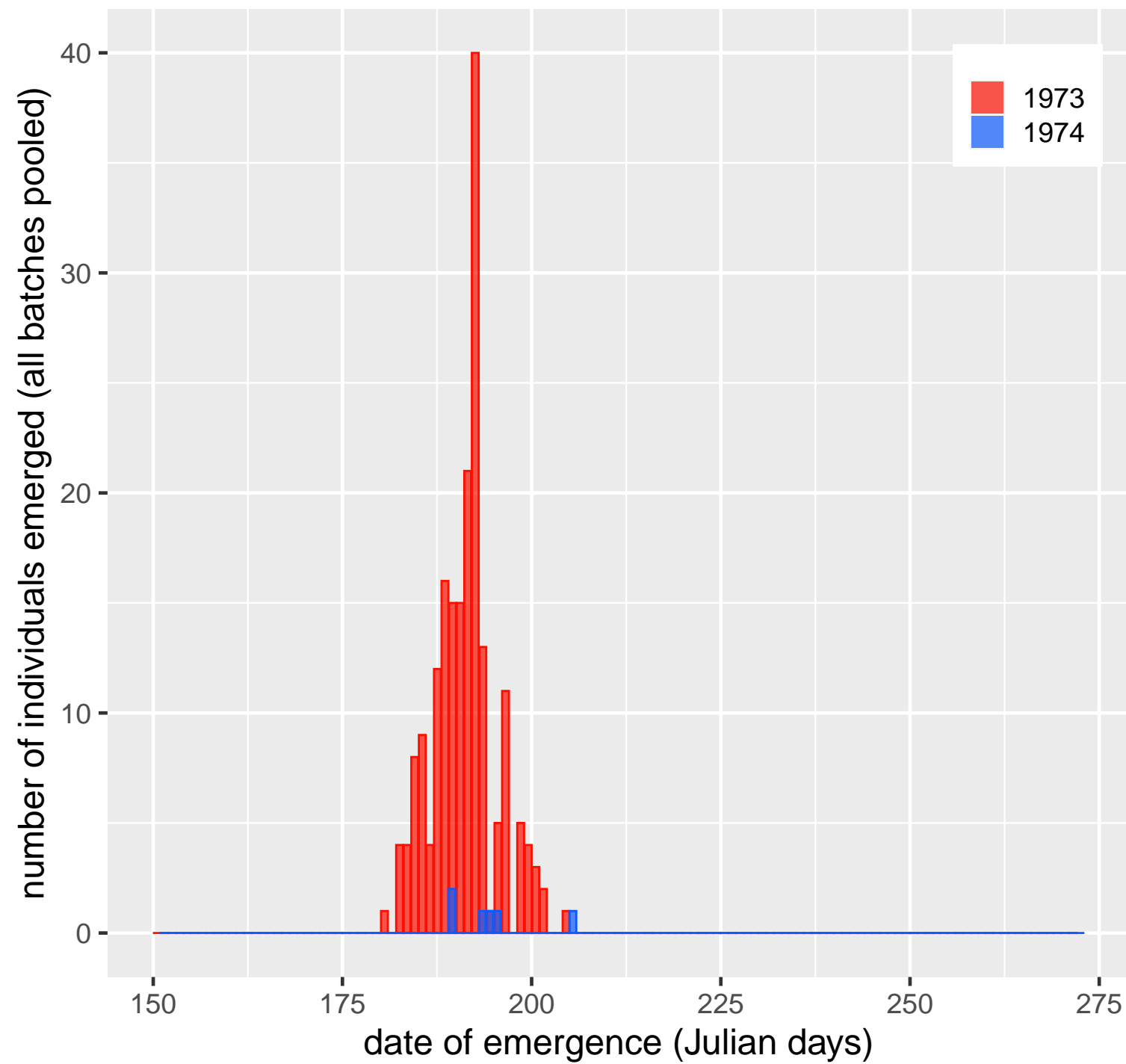



# B697 – 1975

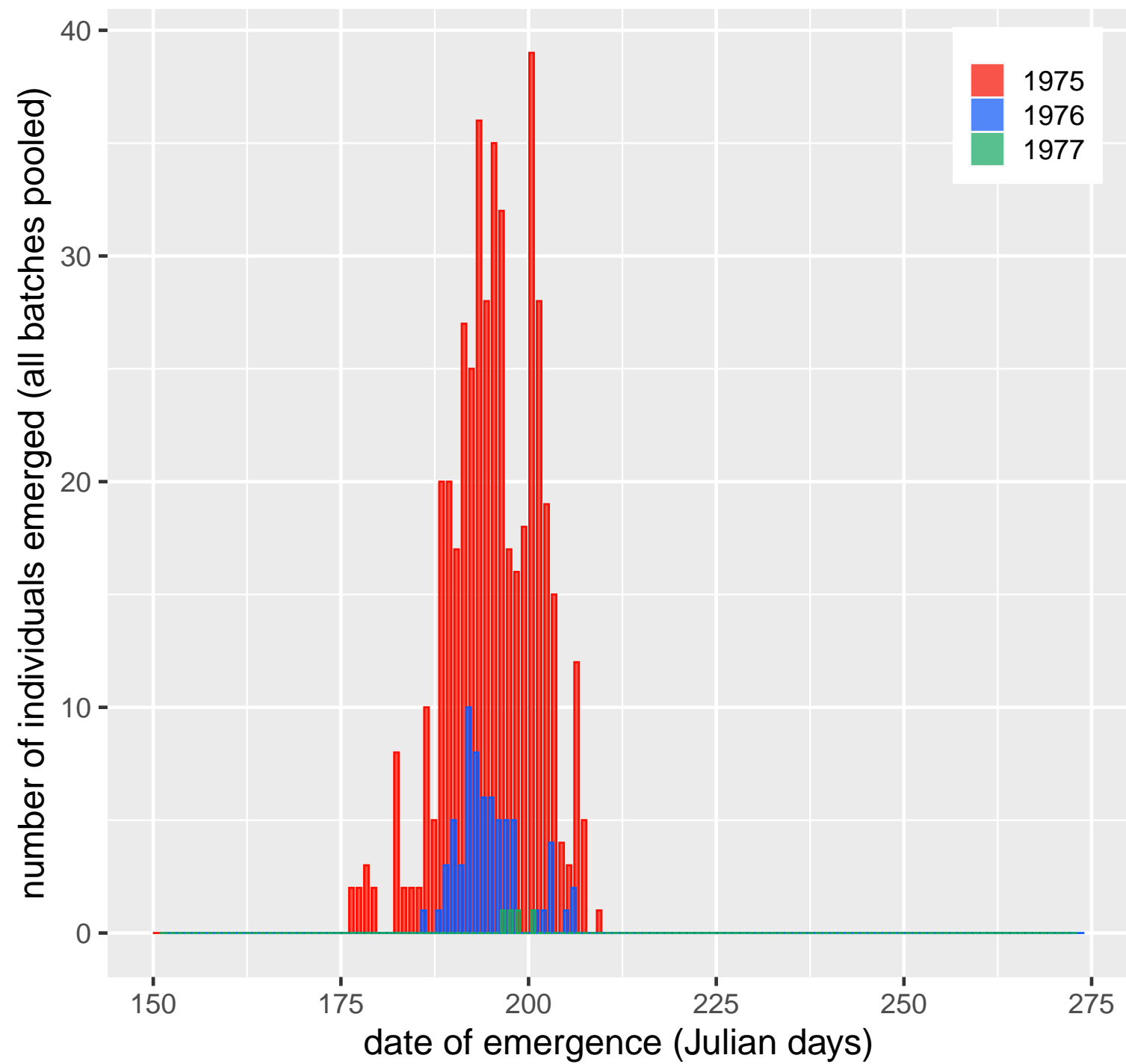



# B697 – 1977

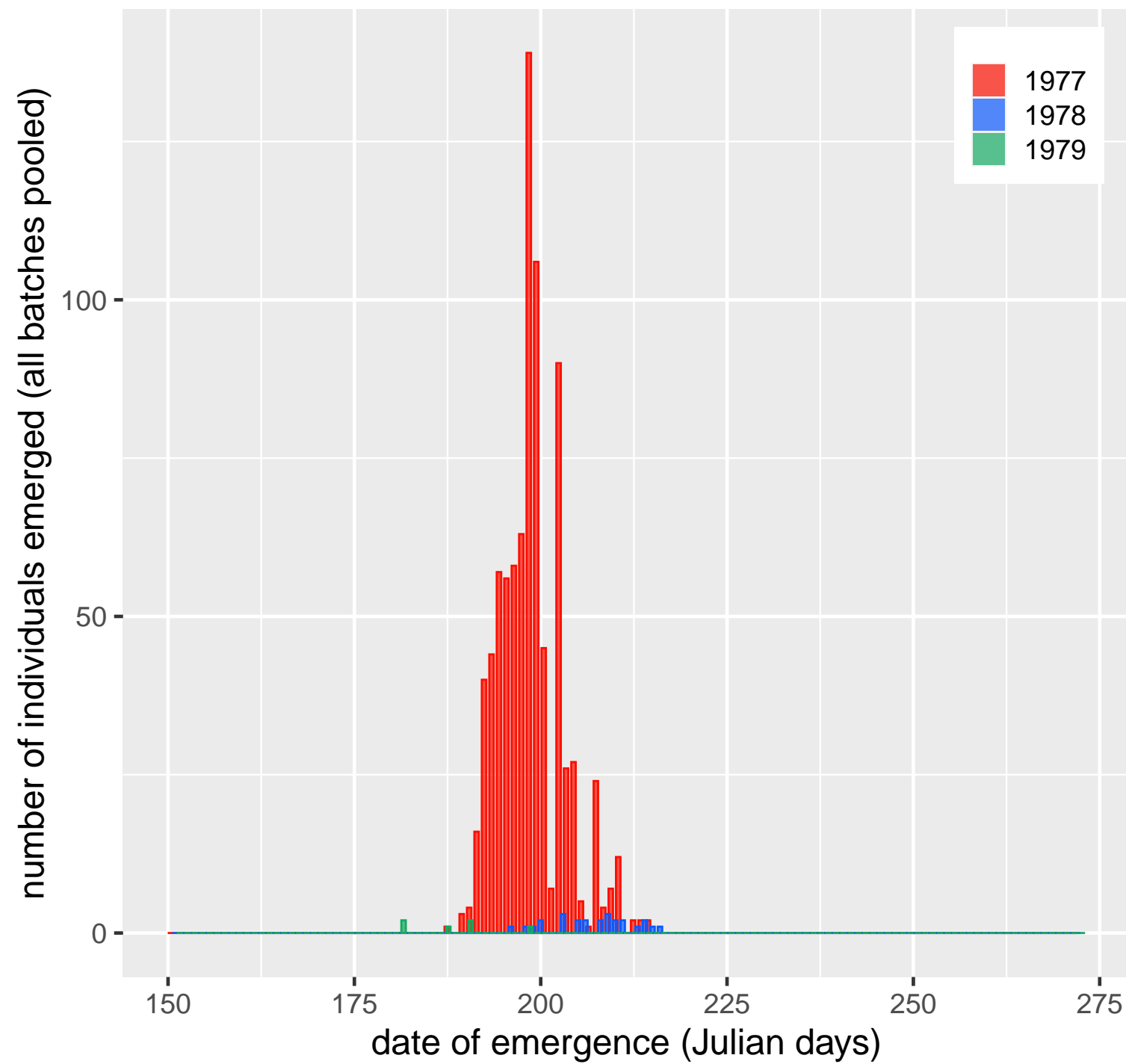

# B697 – 1978

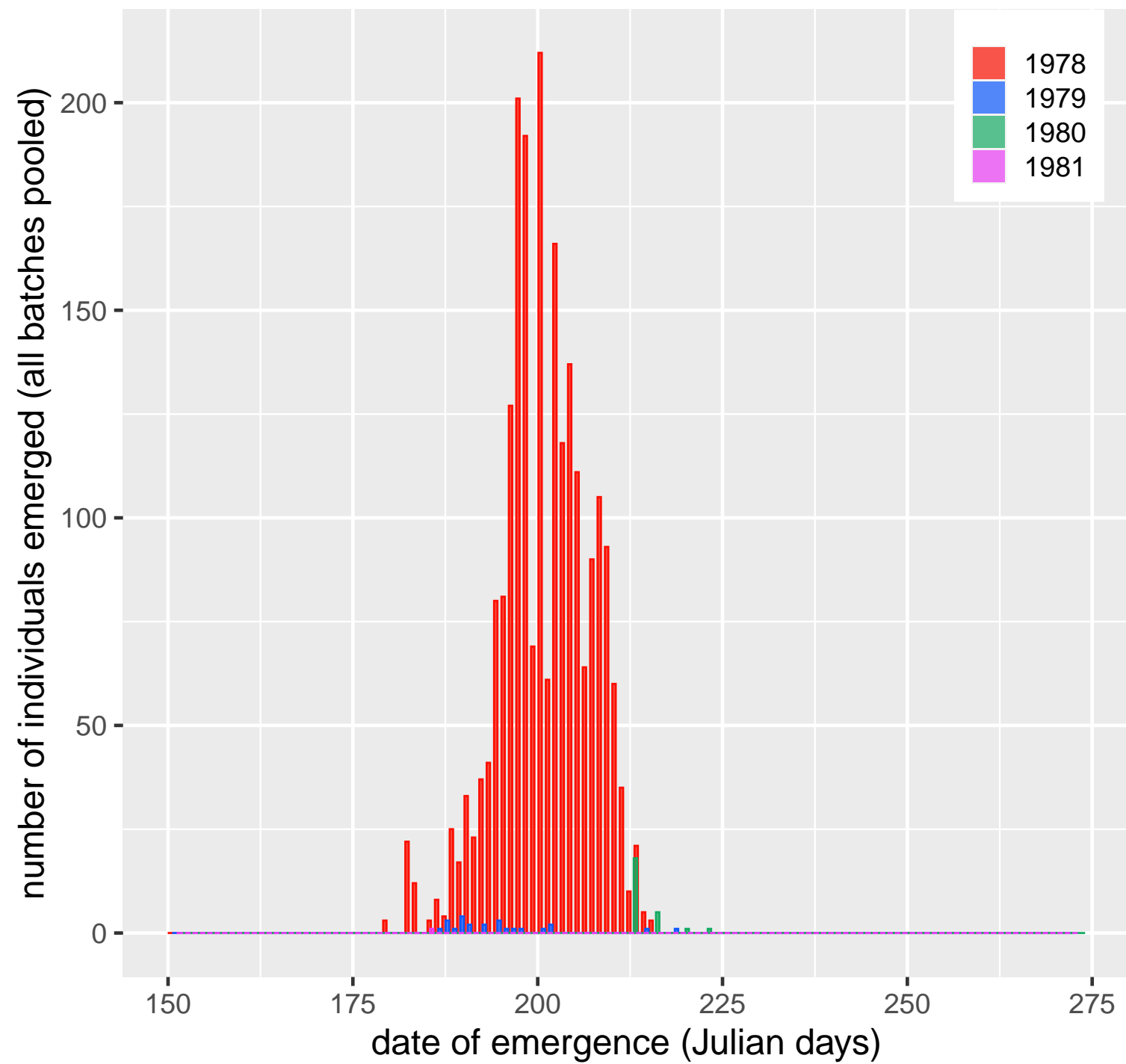

# B697 – 1980

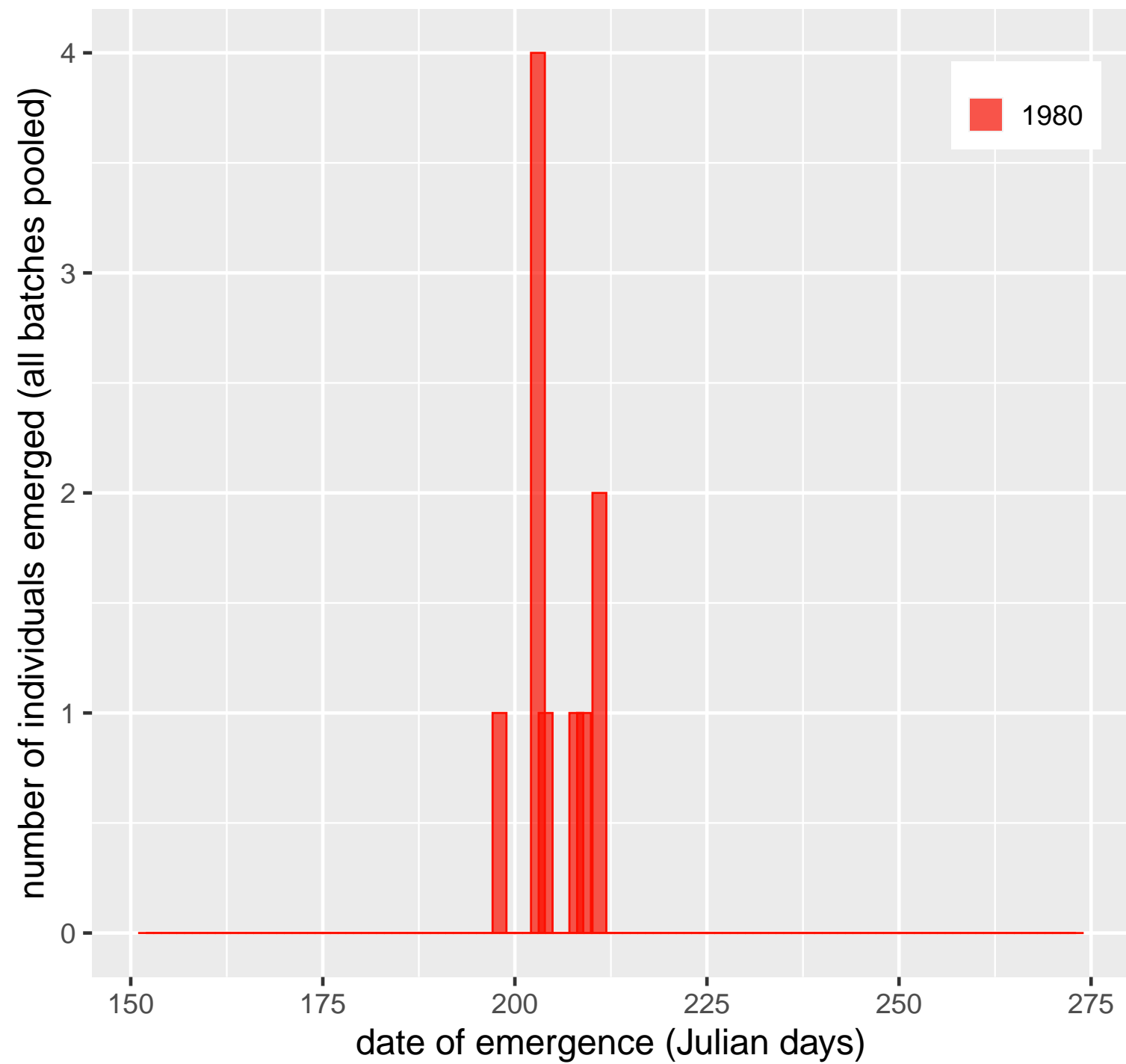

Supplement: Supplementary material 4 — Emergence curves for each cohort sampled in site B697 [file bdj-09-e61086-s004.pdf]

# F781 – 1970

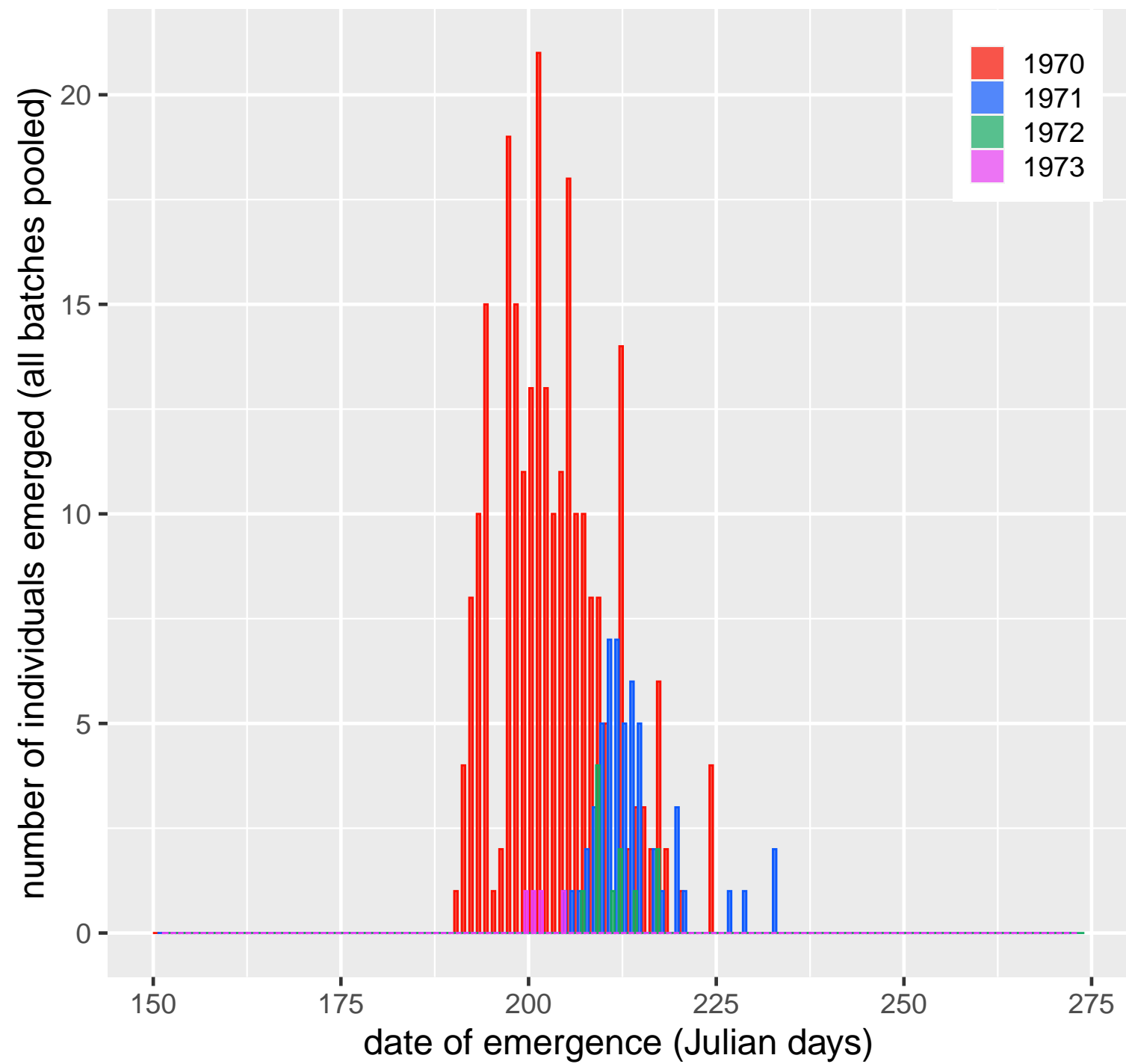

# F781 – 1971

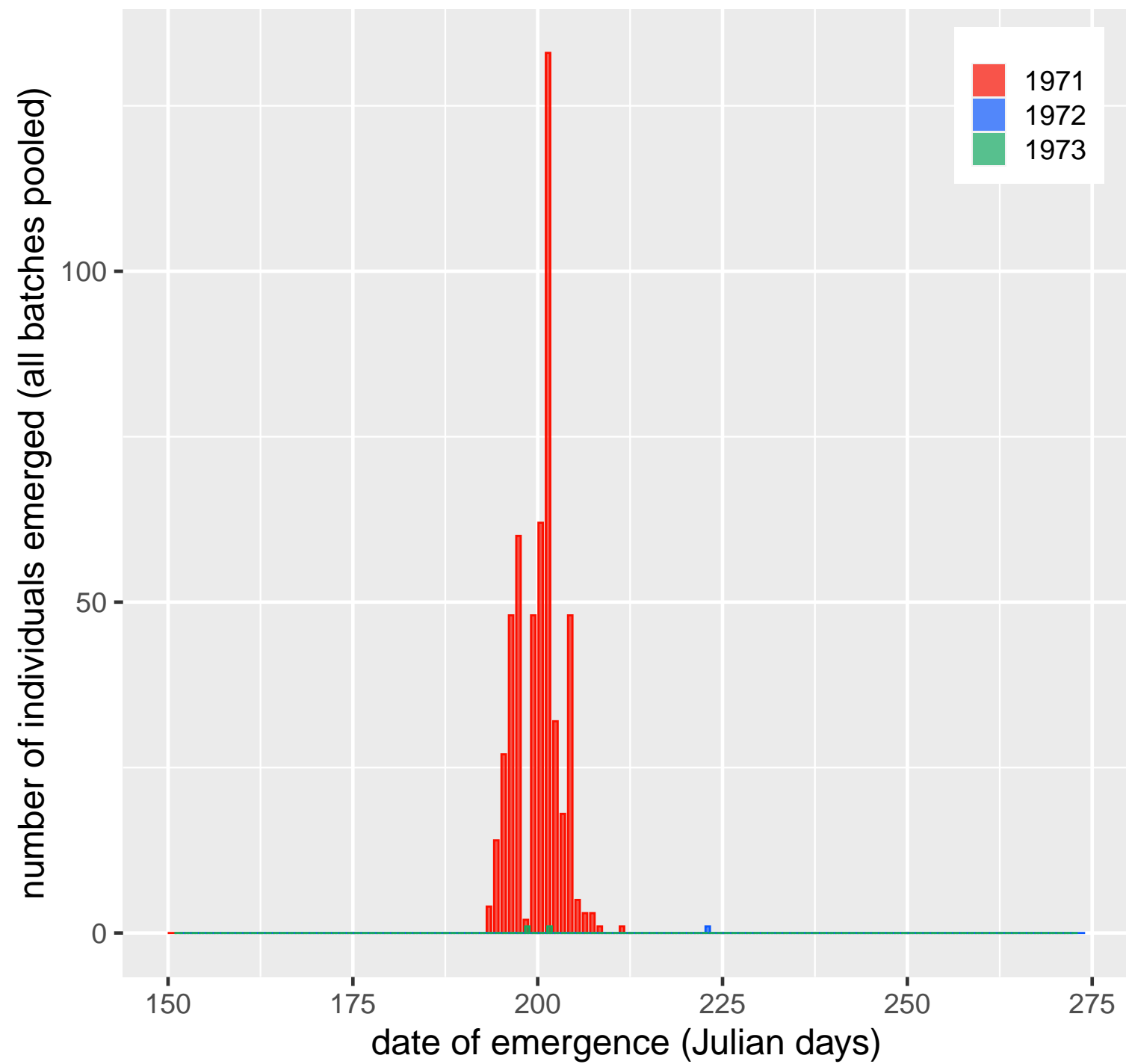

# F781 – 1972

number of individuals emerged (all batches pooled)

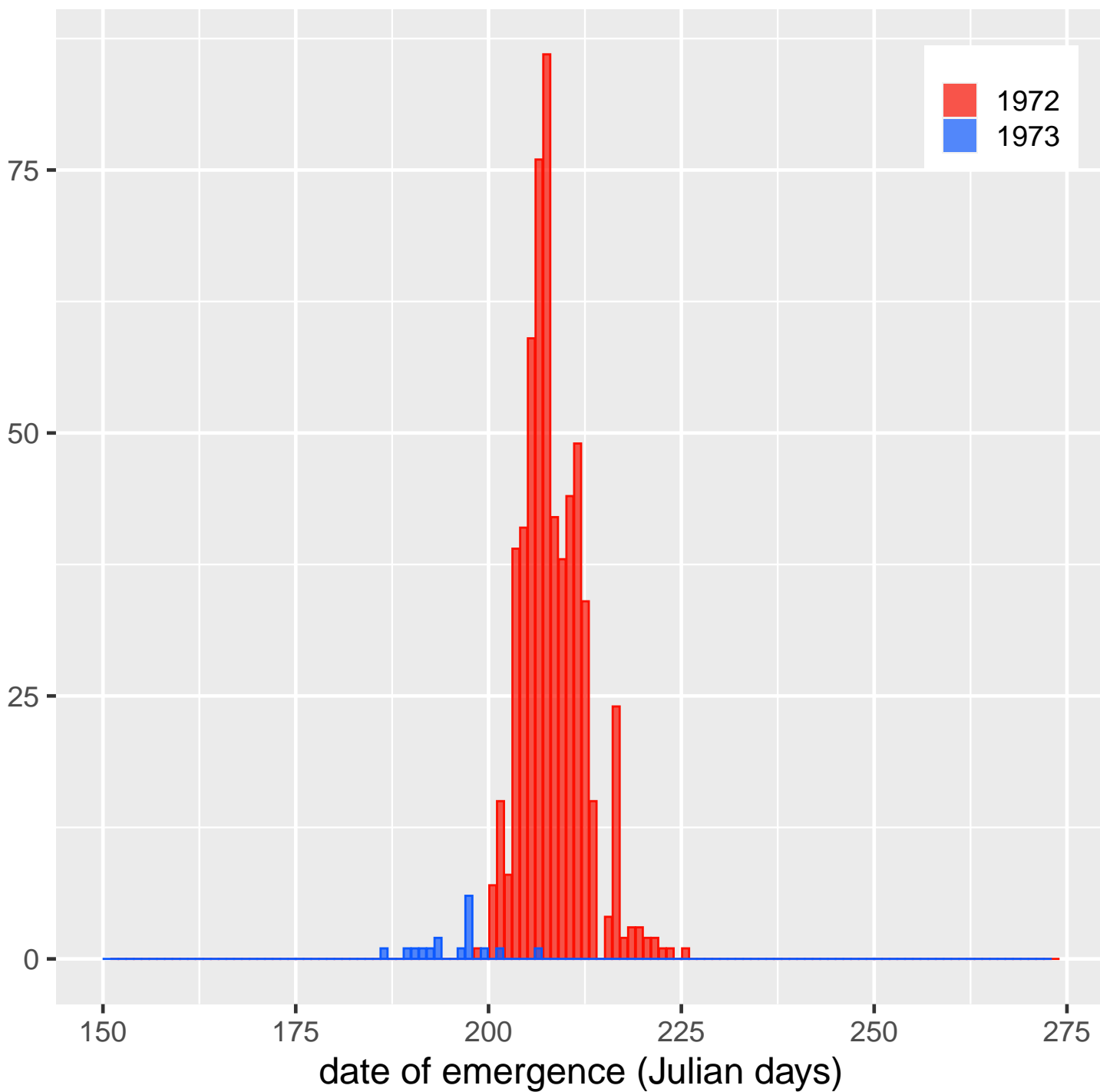

# F781 – 1973

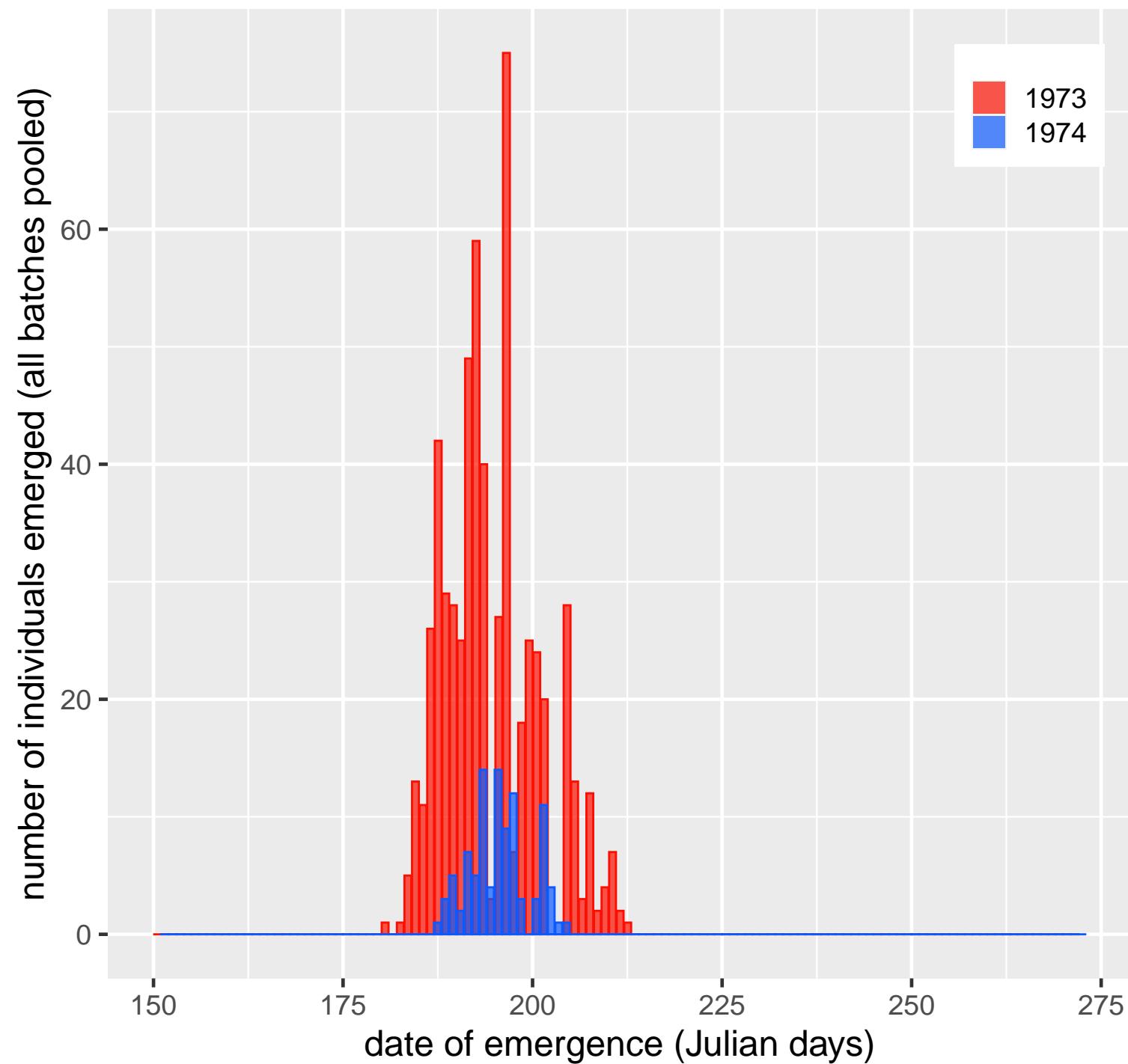

# F781 – 1974

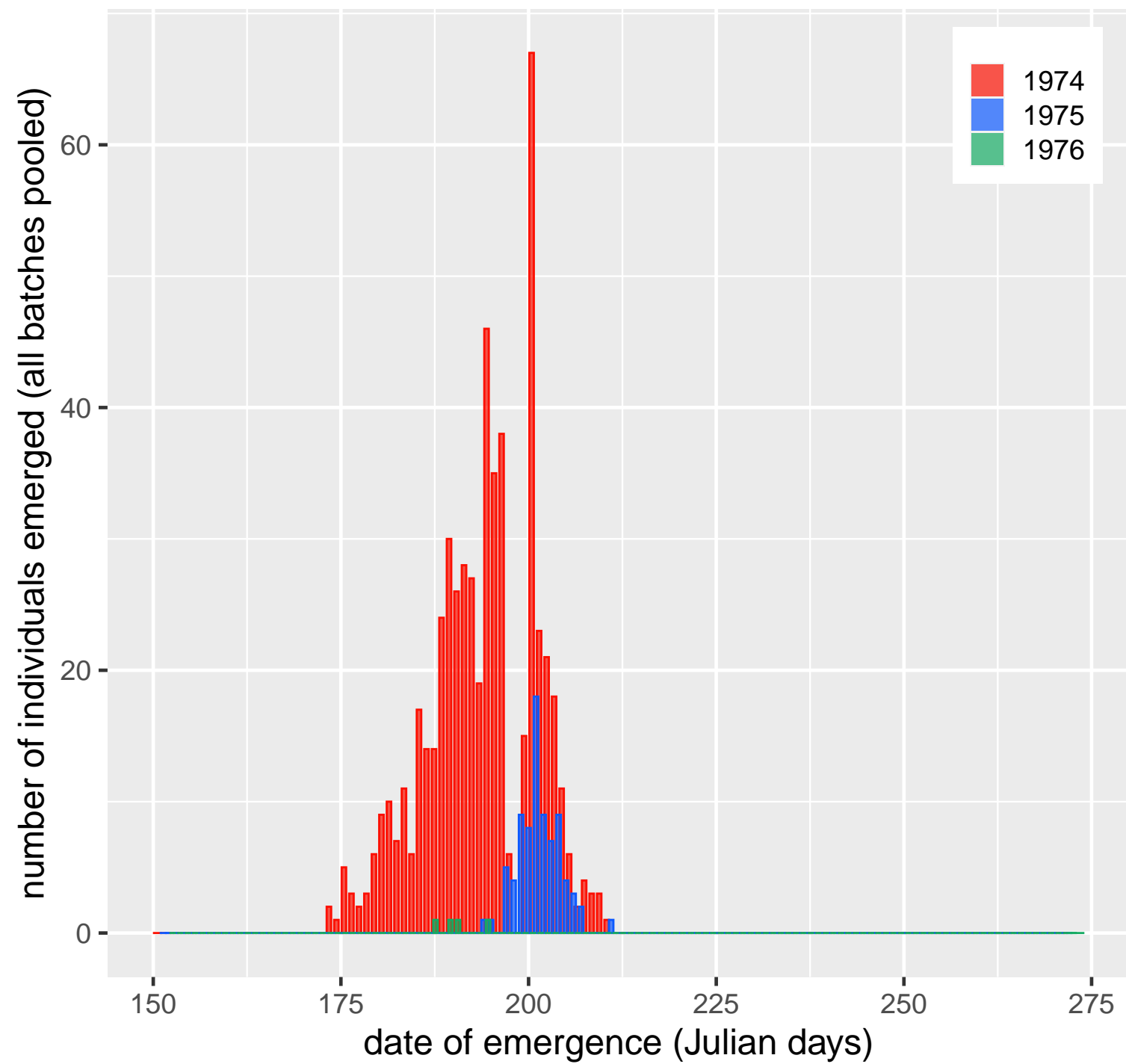

# F781 – 1976

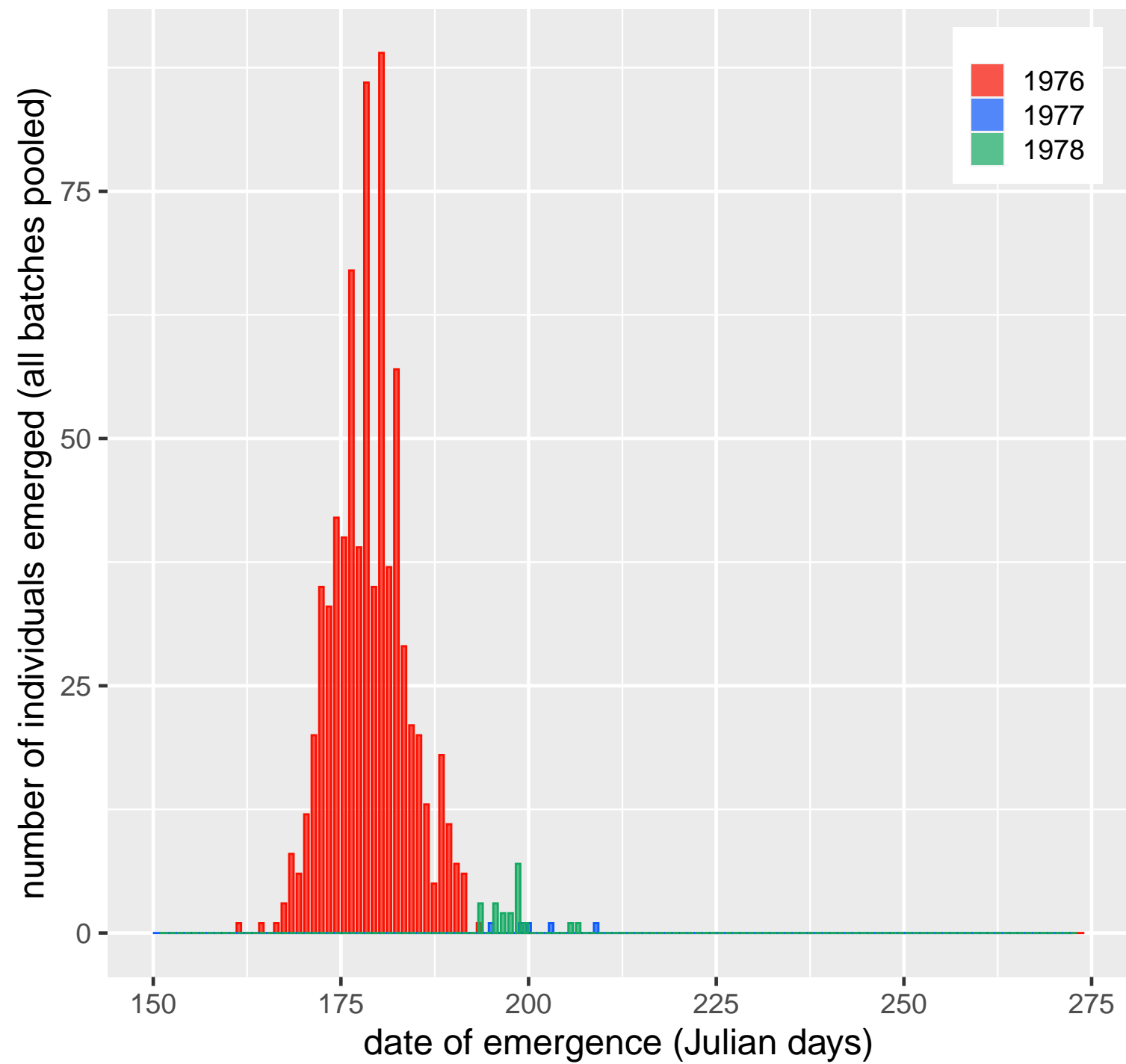

# F781 – 1977

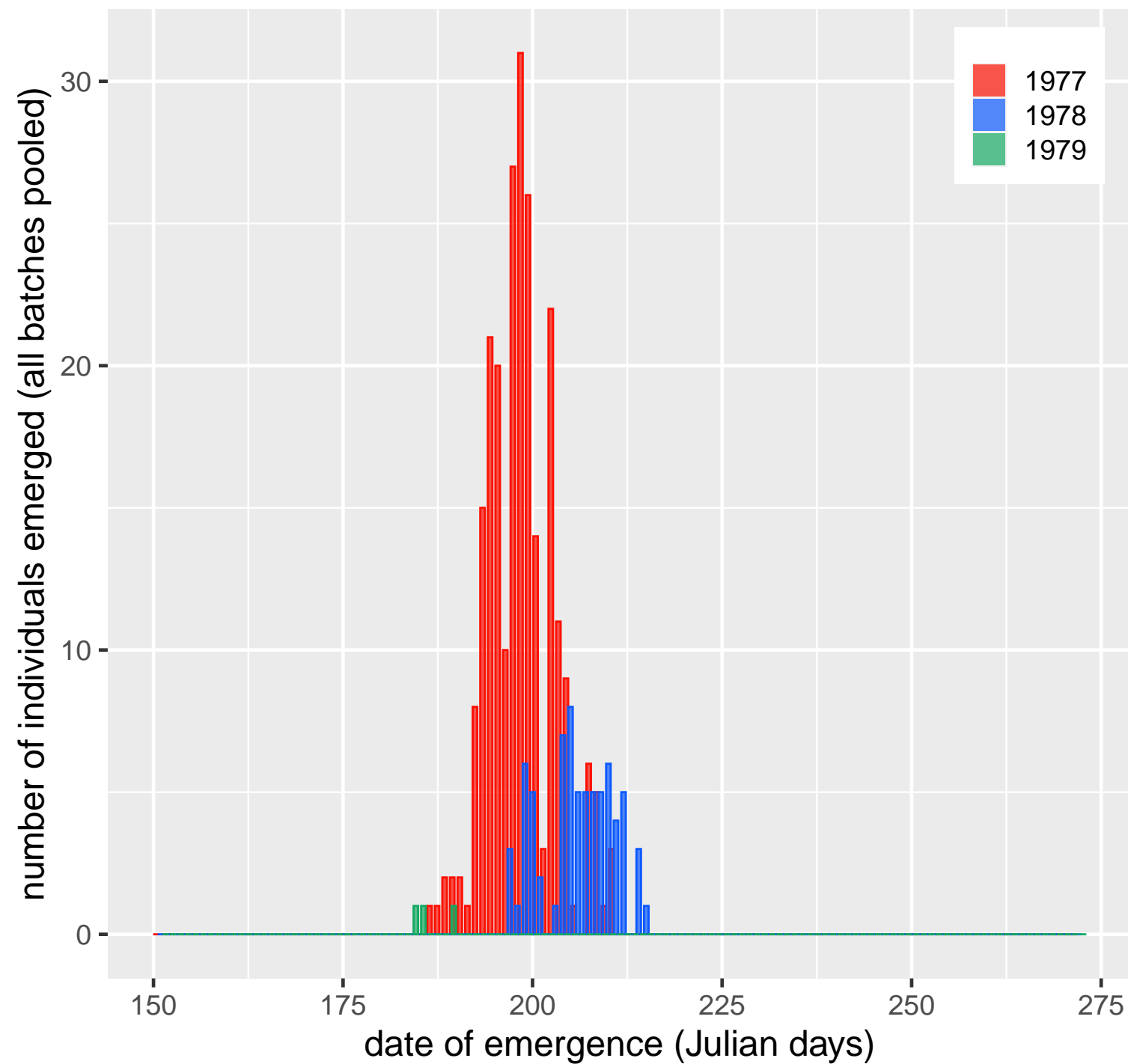

# F781 – 1978

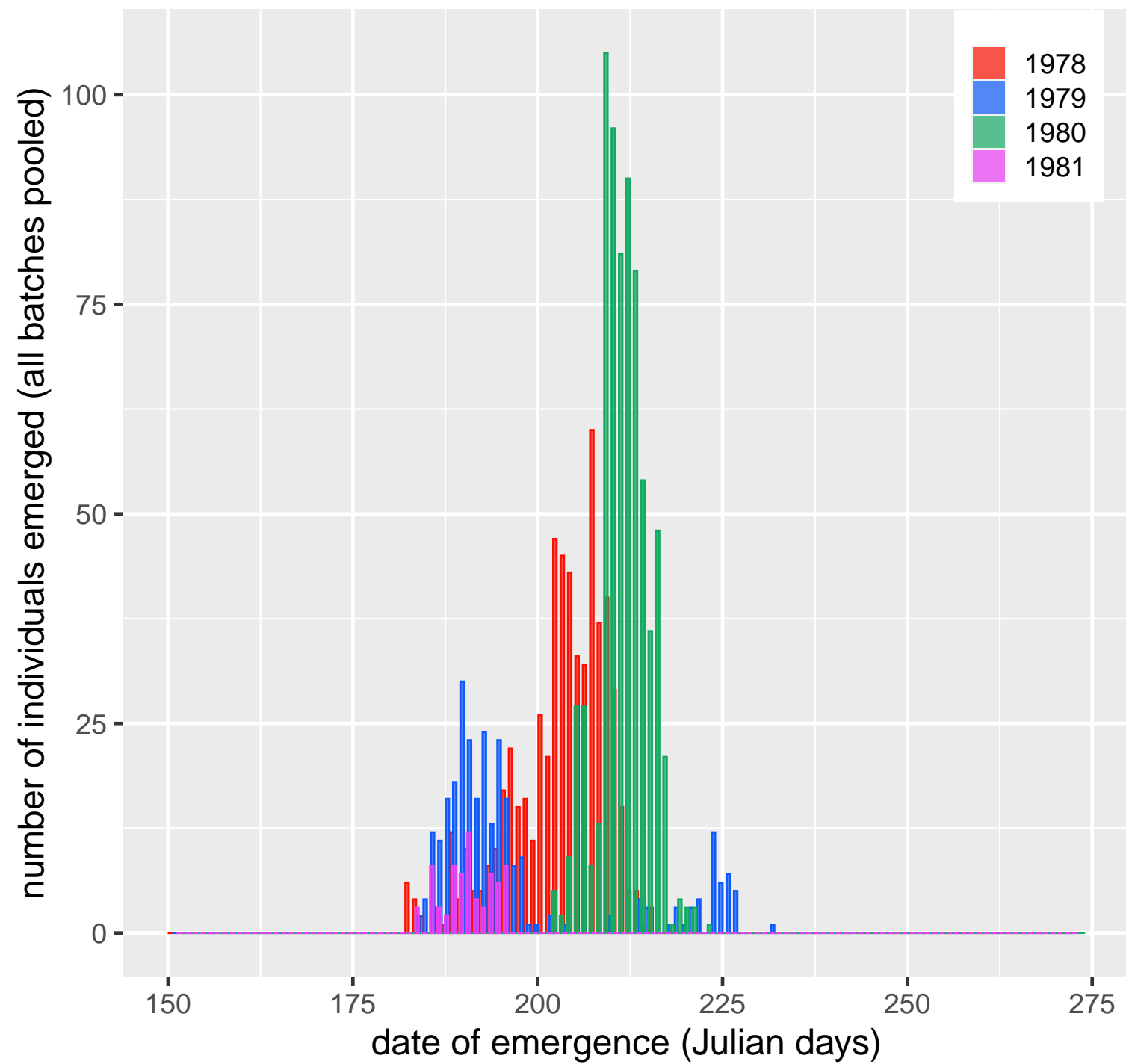

# F781 – 1979

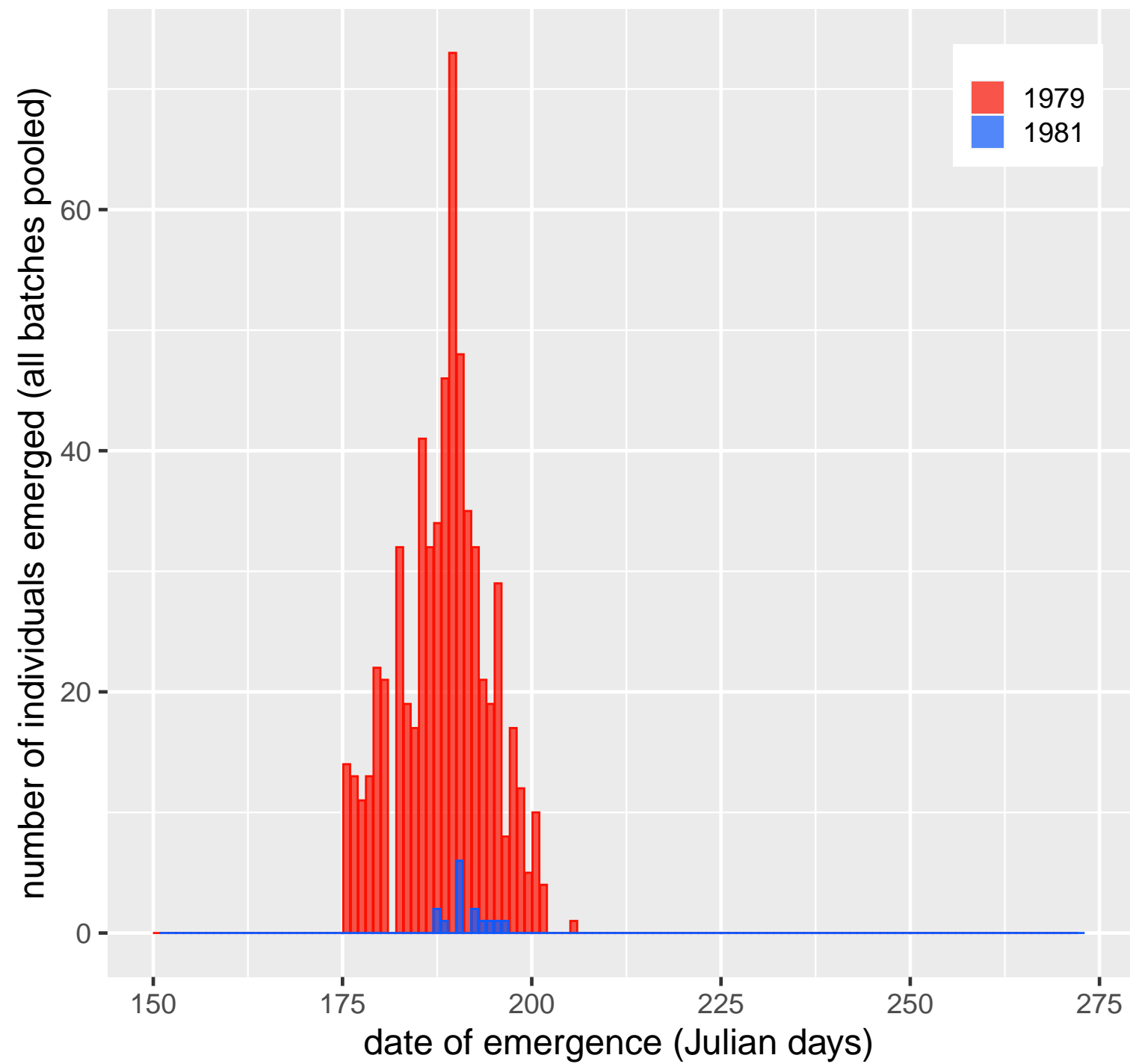

# F781 – 1980

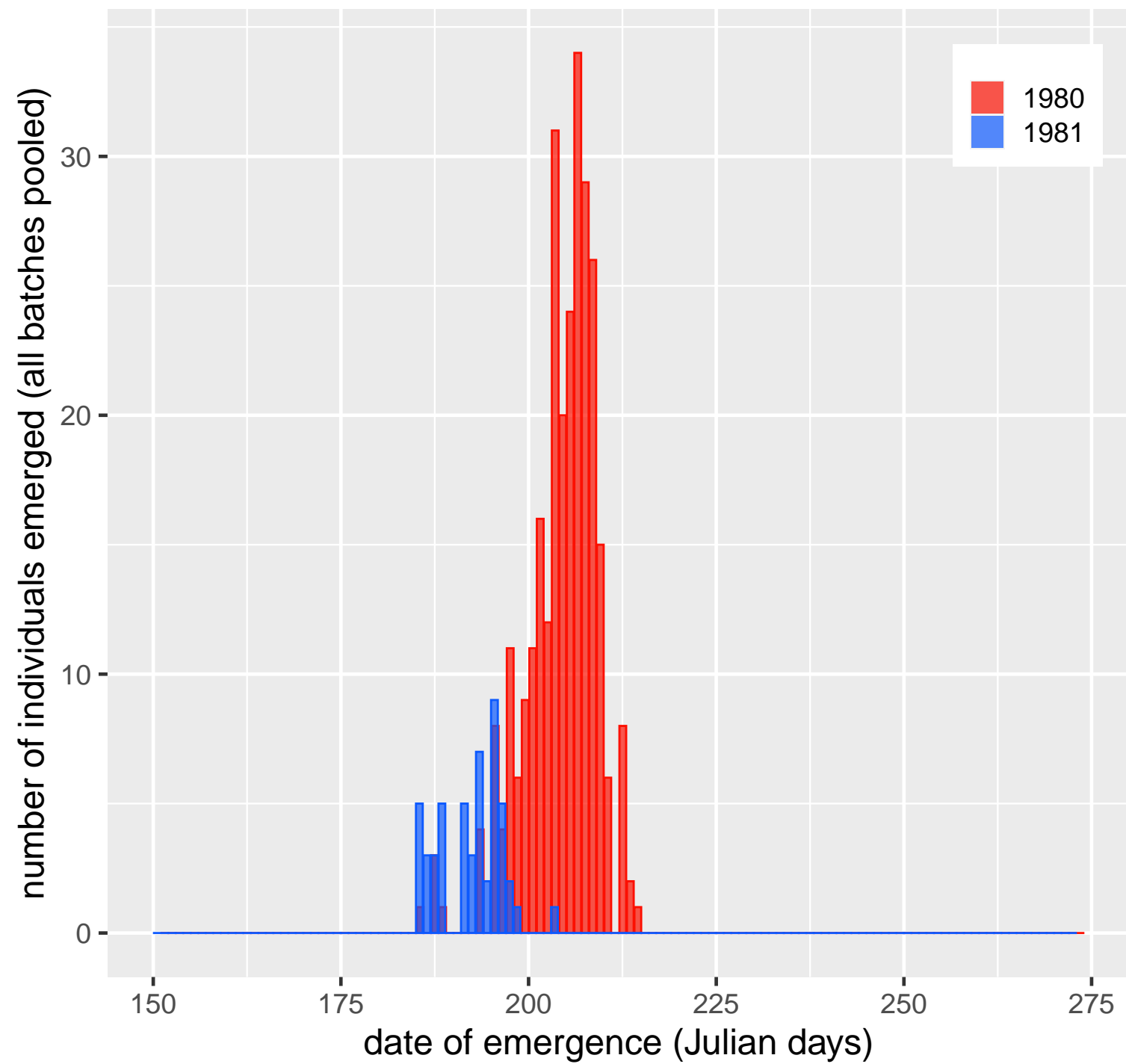

# F781 – 1982

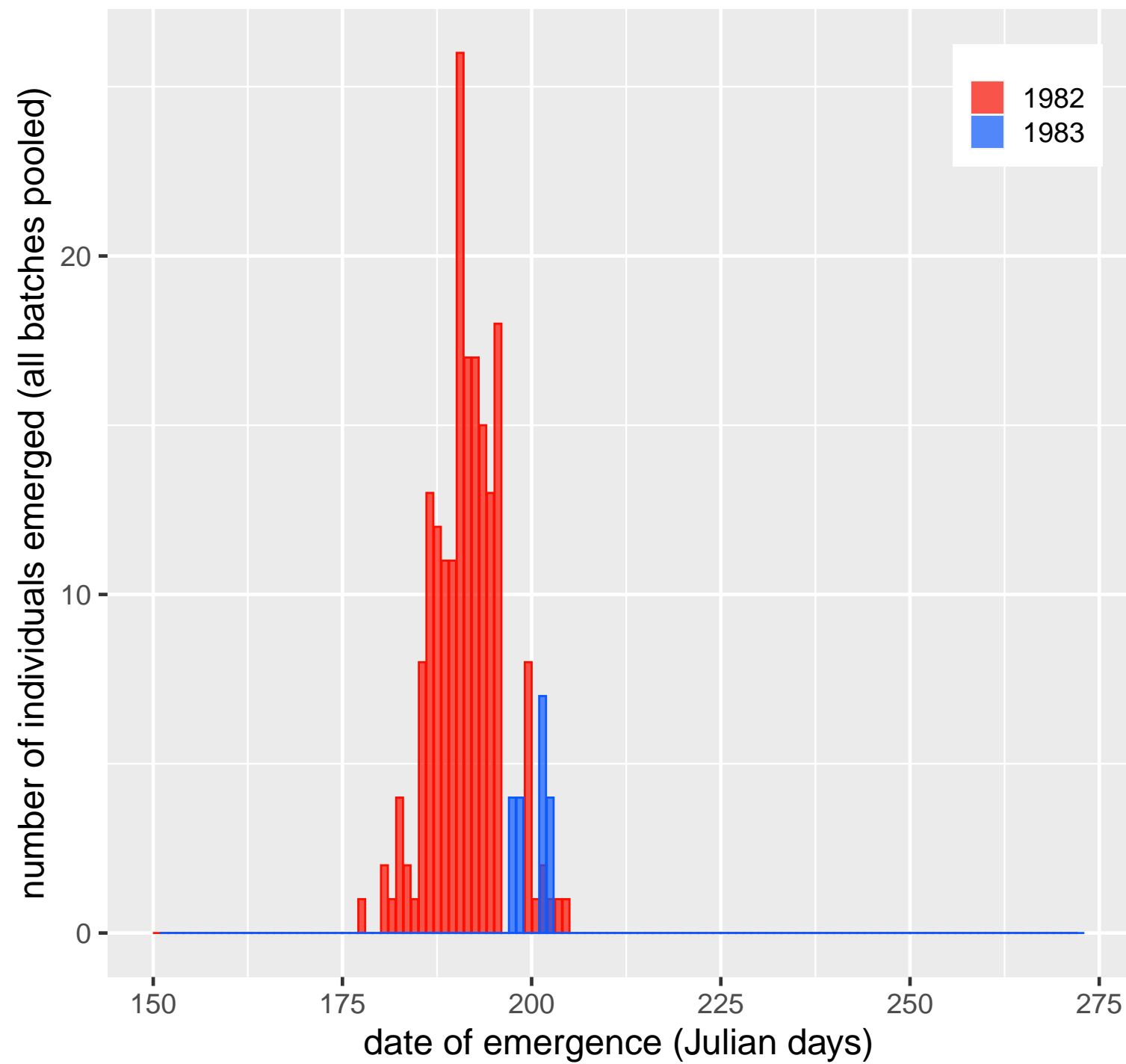

# F781 – 1983

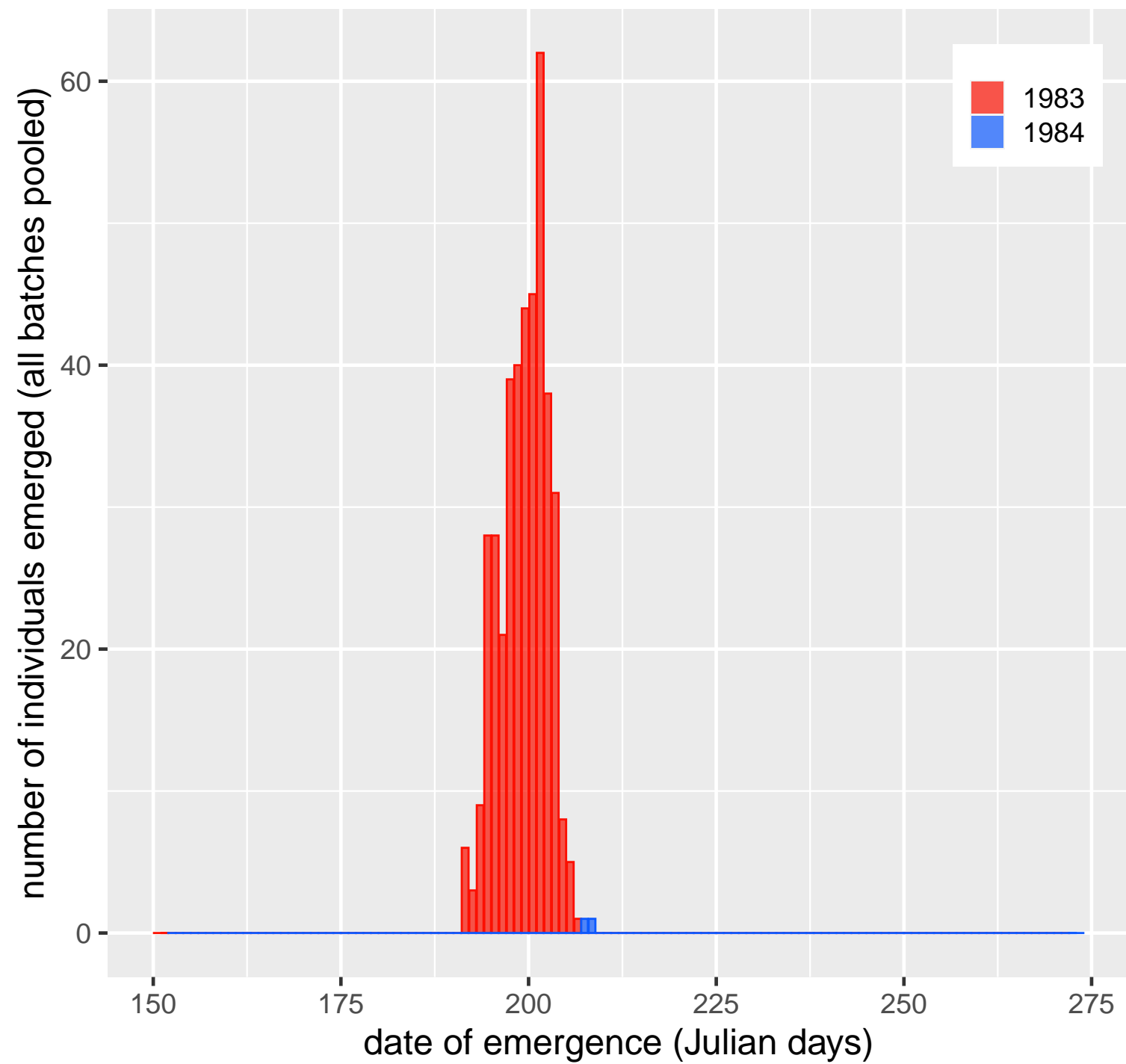

# F781 – 1984

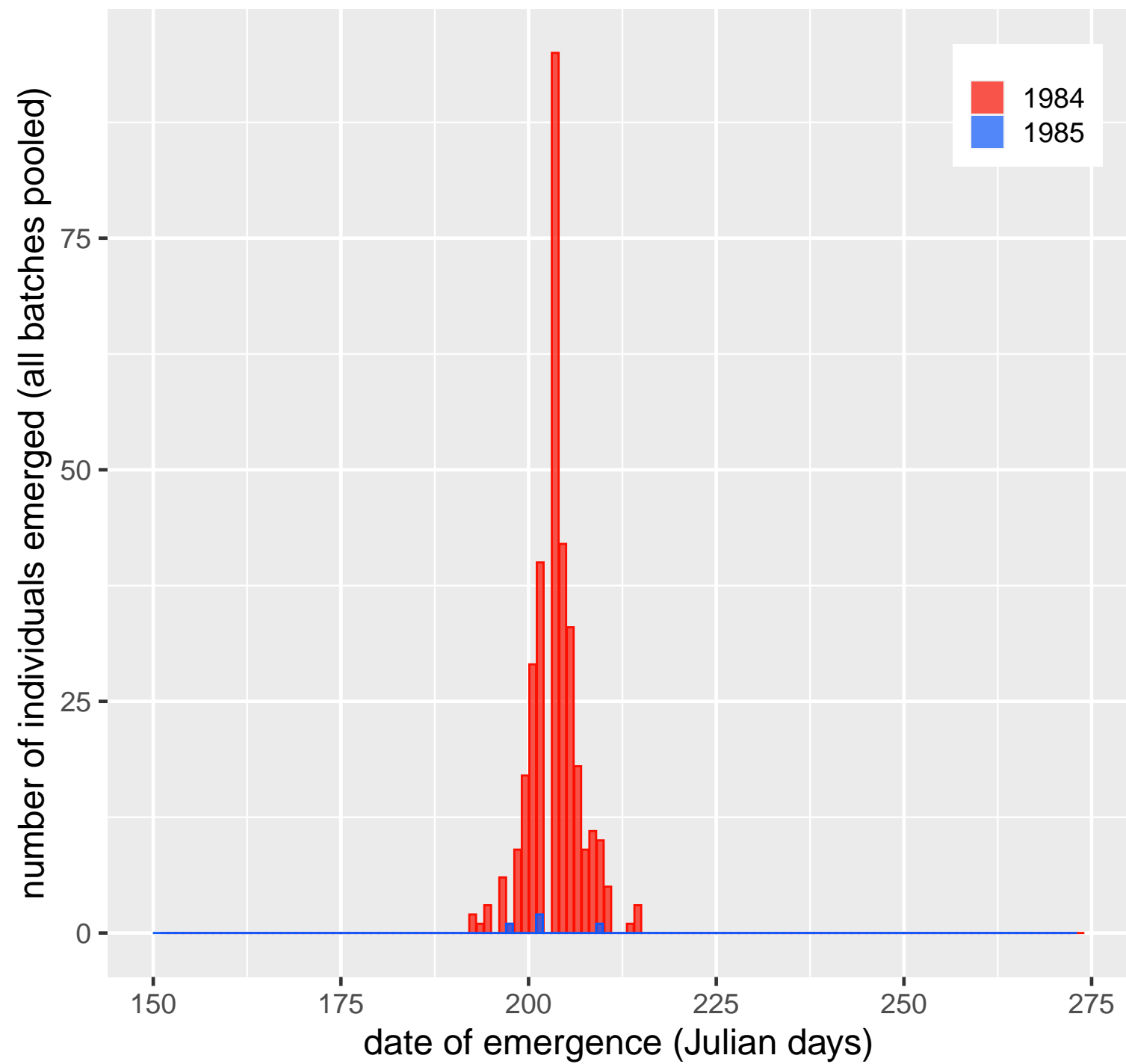

Supplement: Supplementary material 5 — Emergence curves for each cohort sampled in site F781 [file bdj-09-e61086-s005.pdf]

# R923 – 1970

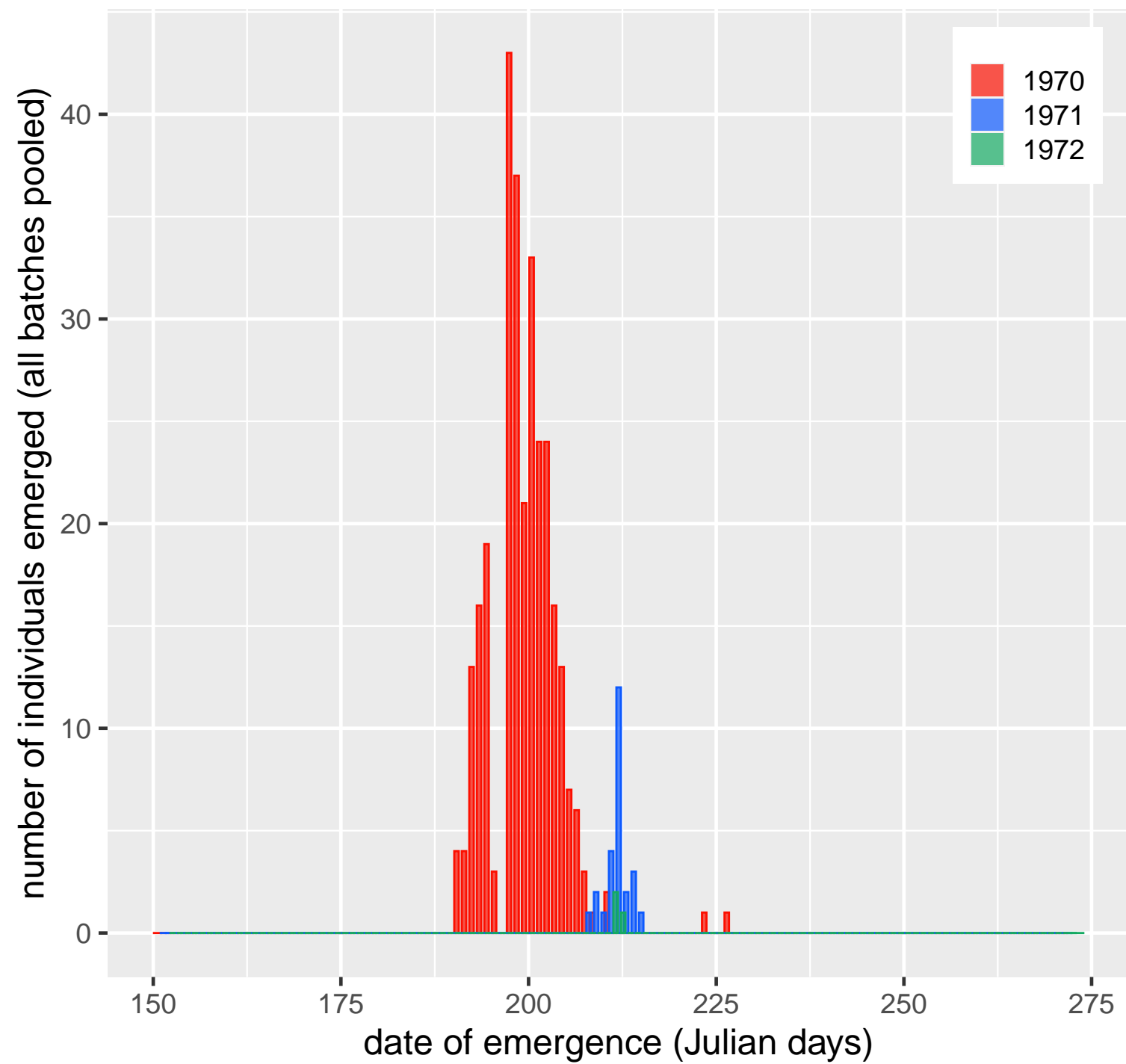

# R923 – 1971

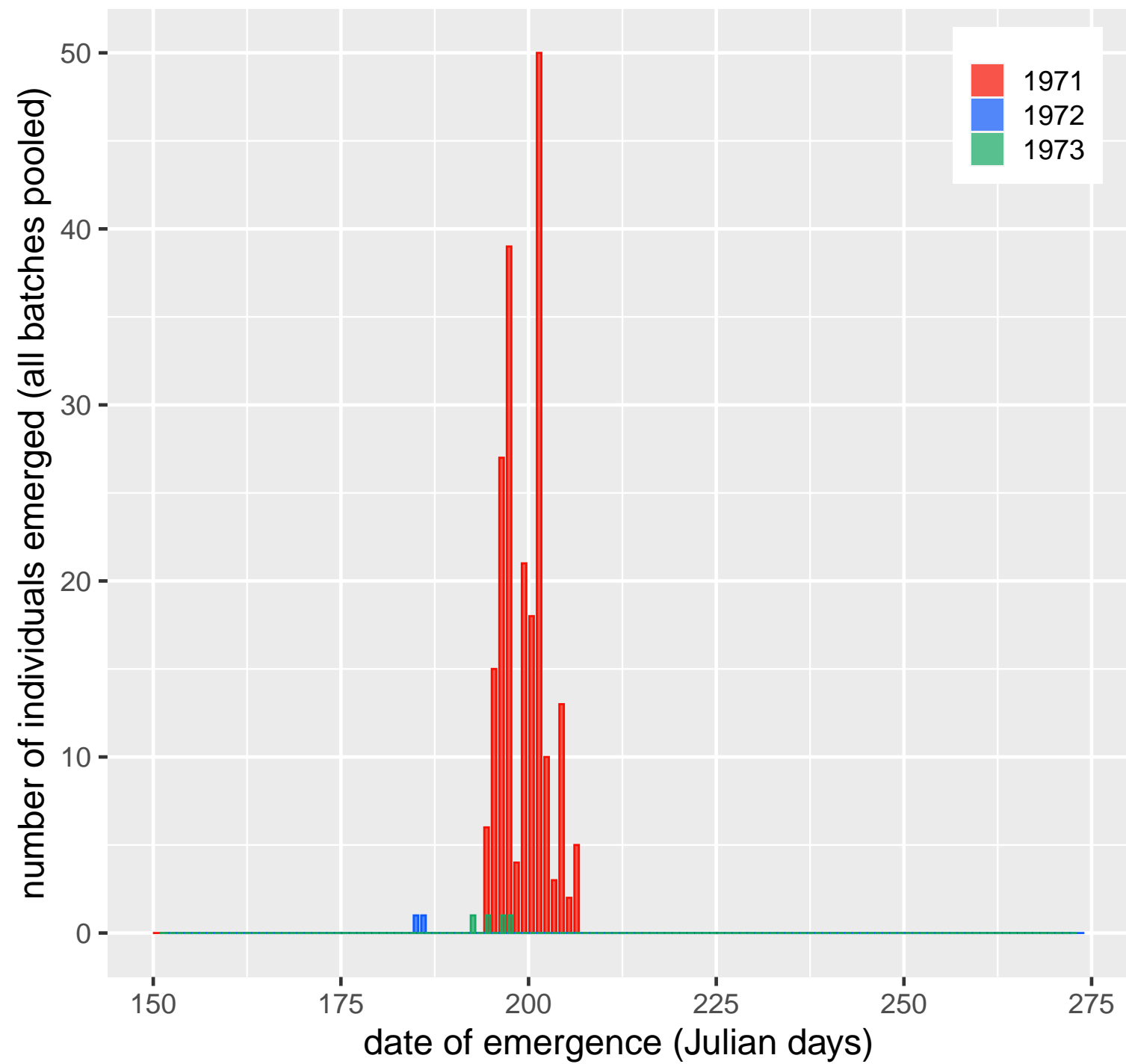

# R923 – 1972

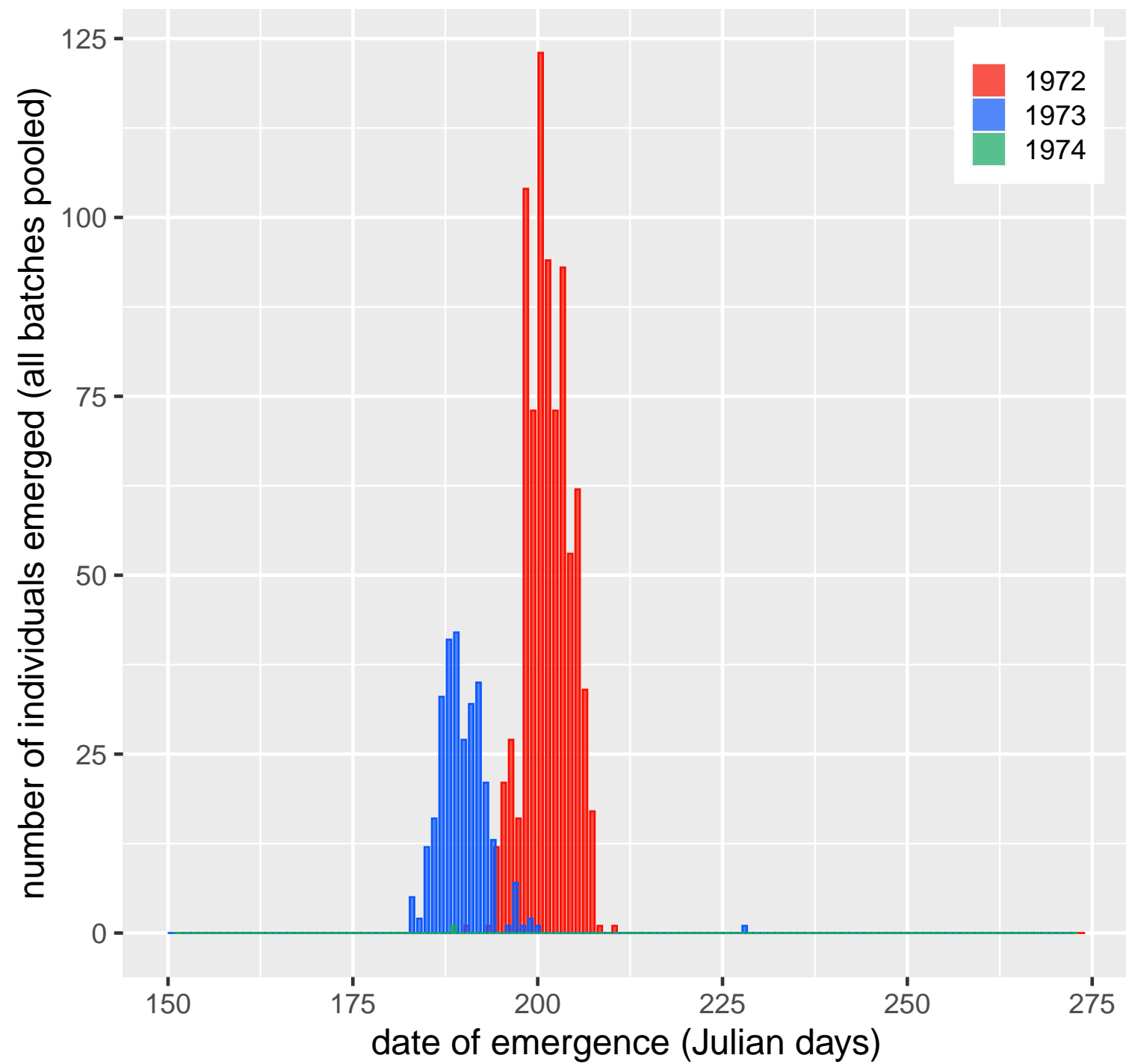

# R923 – 1973

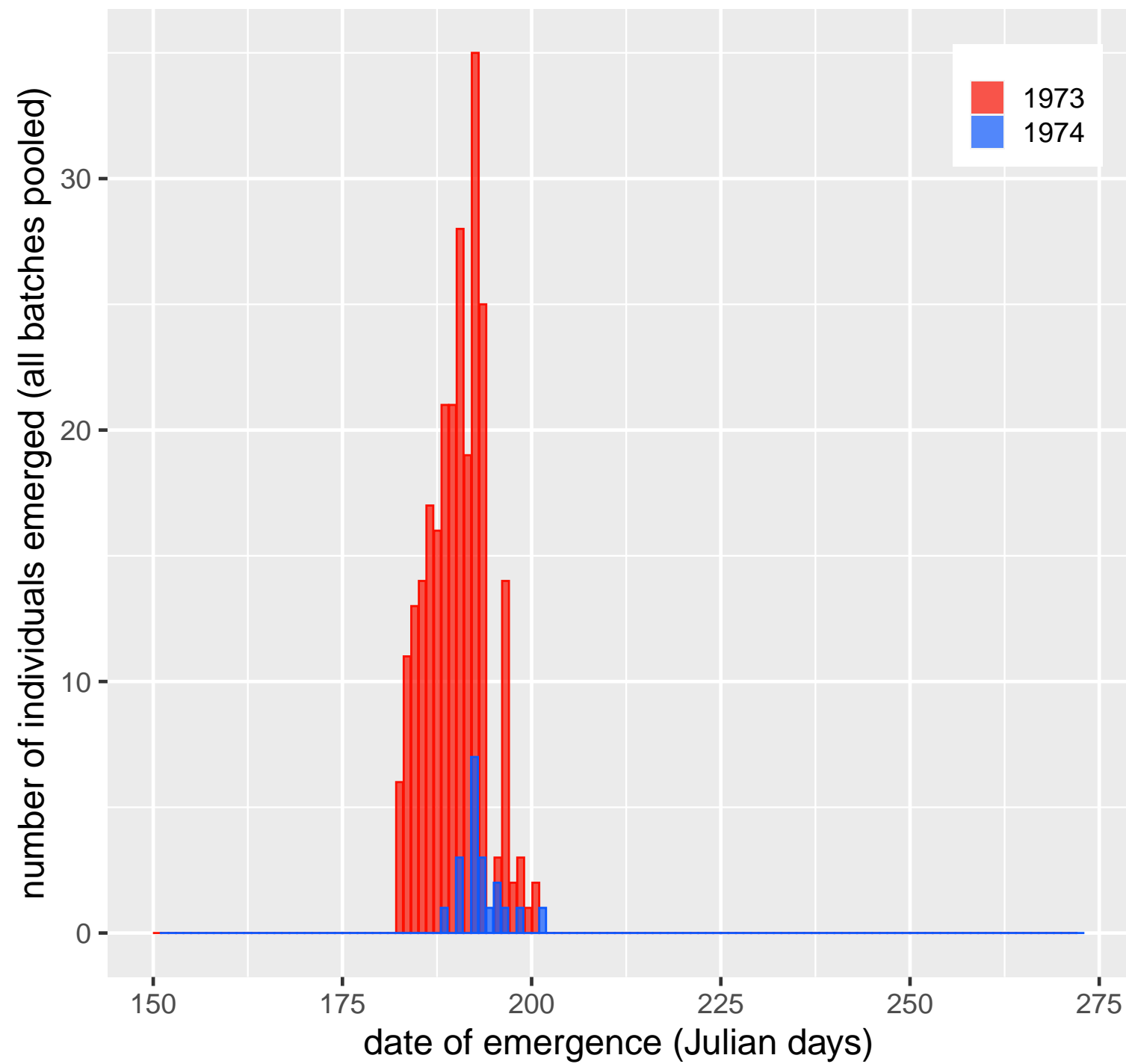

# R923 – 1974

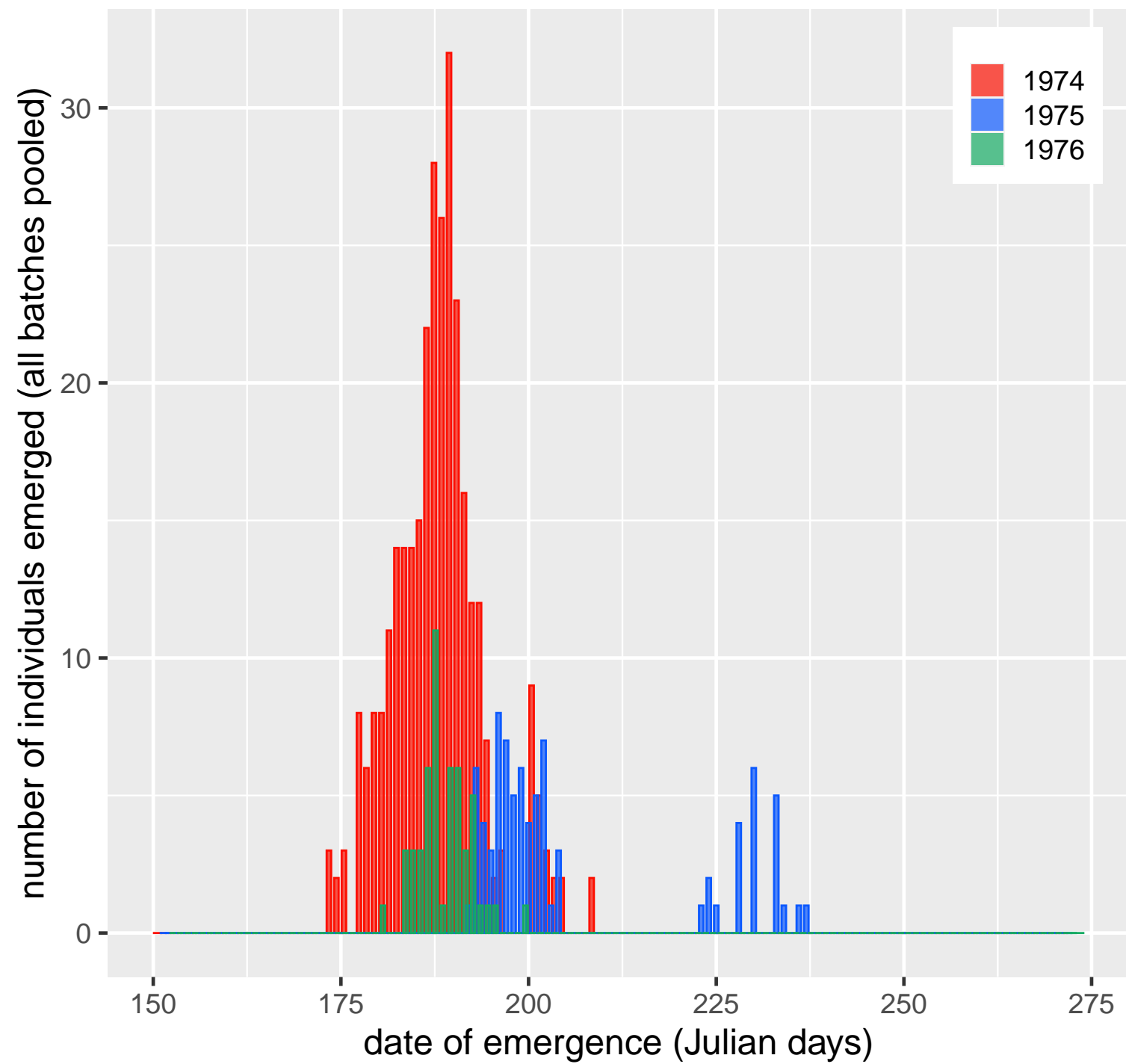

# R923 – 1976

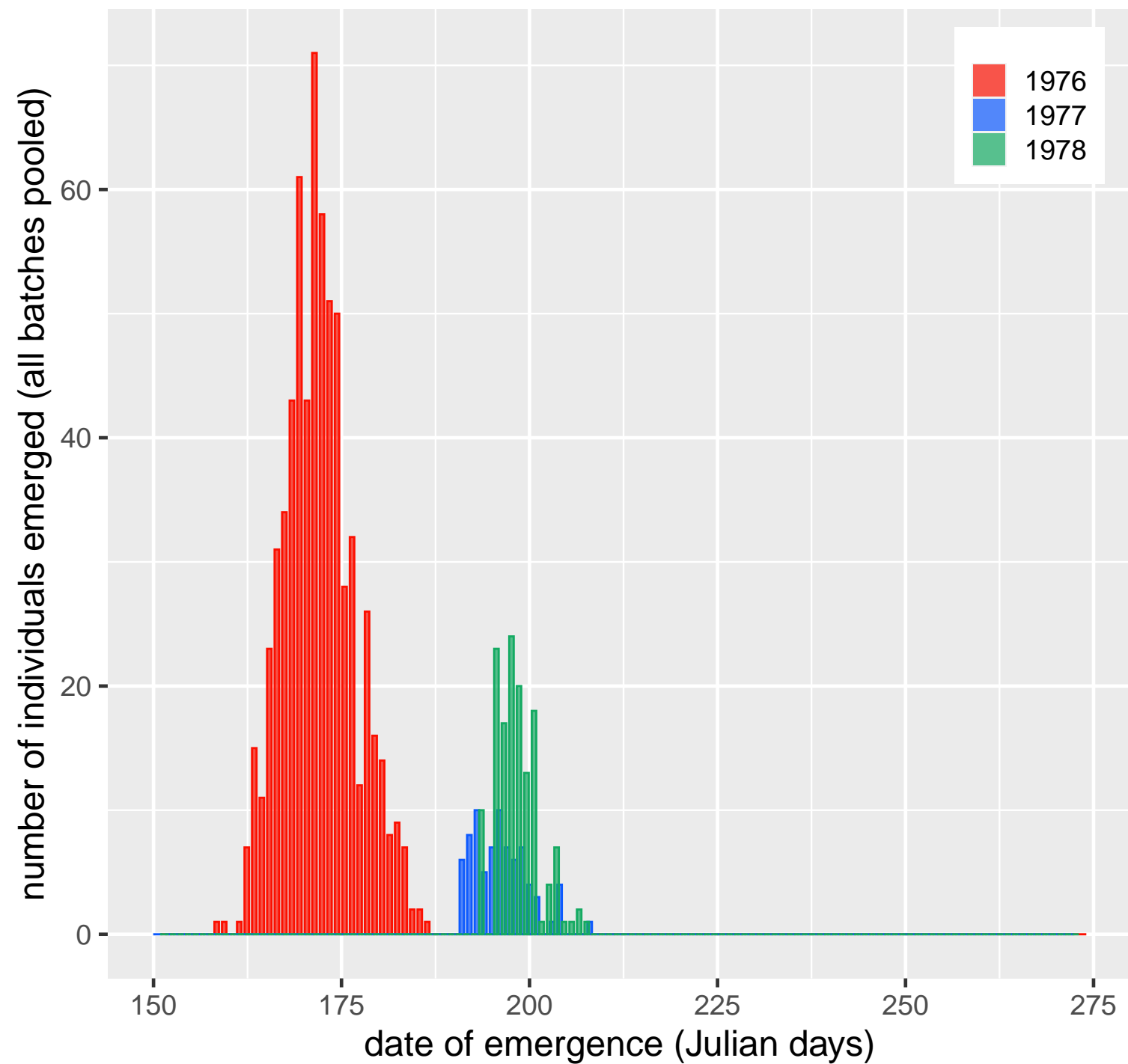

# R923 – 1977

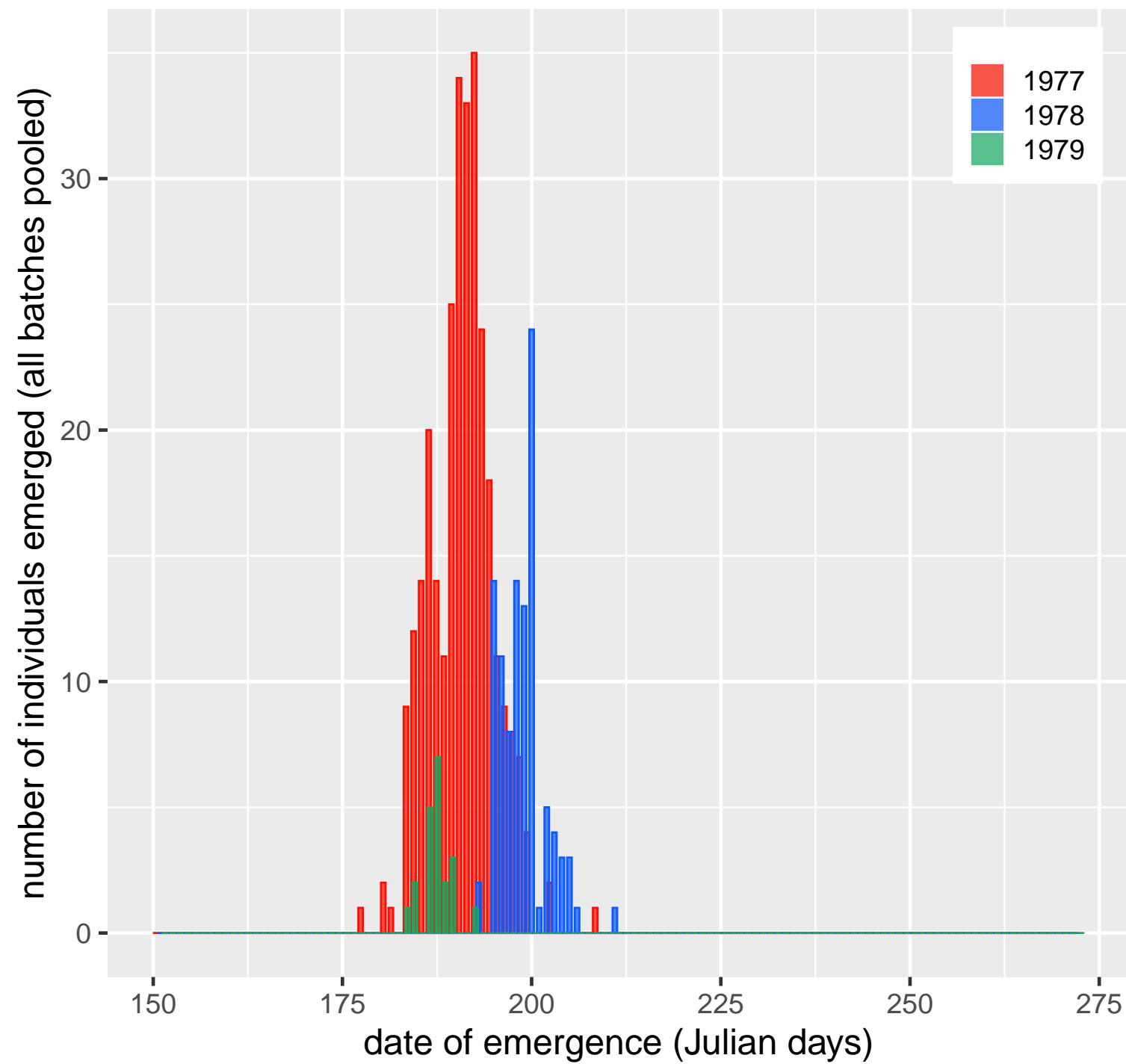

# R923 – 1978

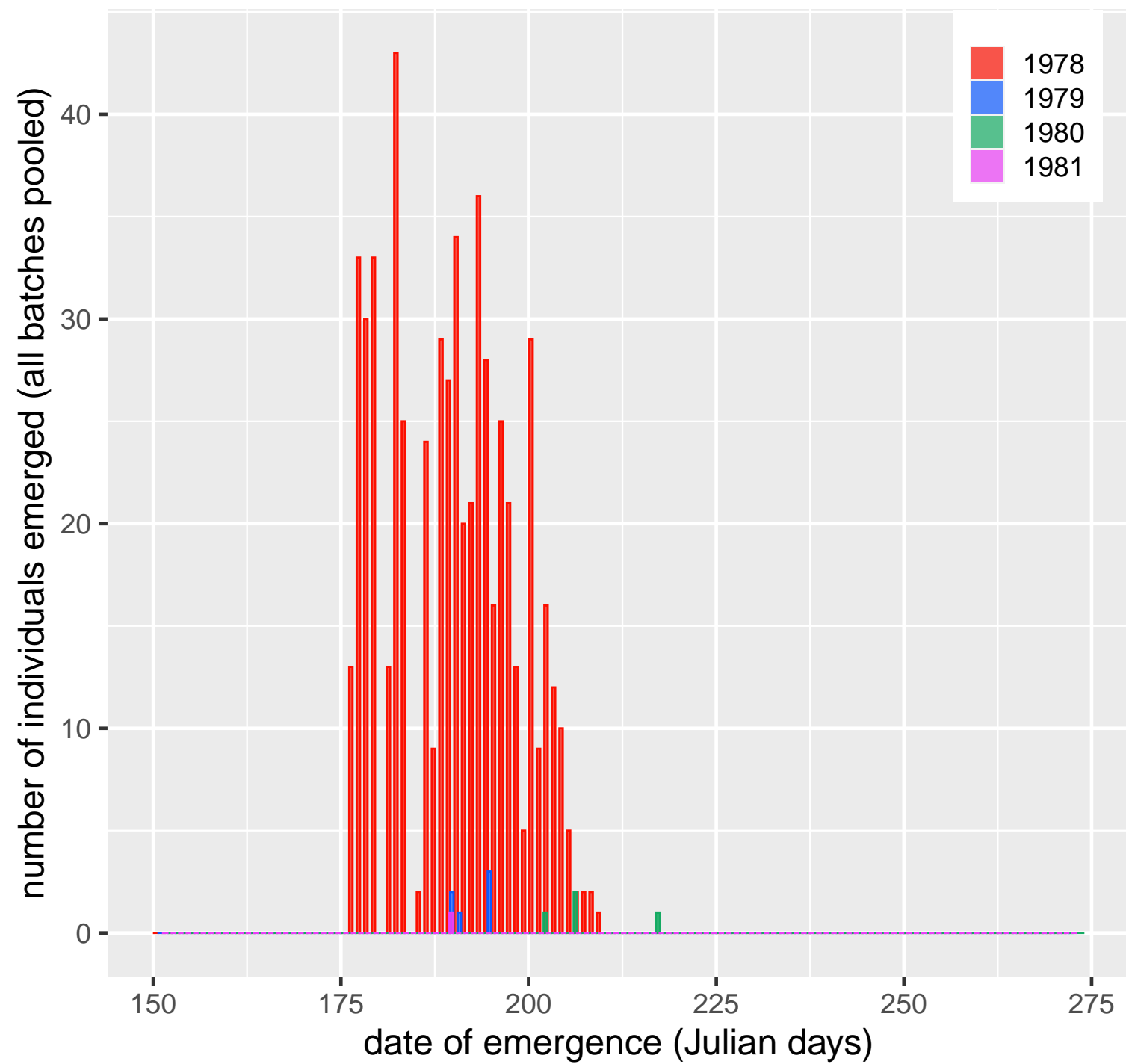

# R923 – 1979

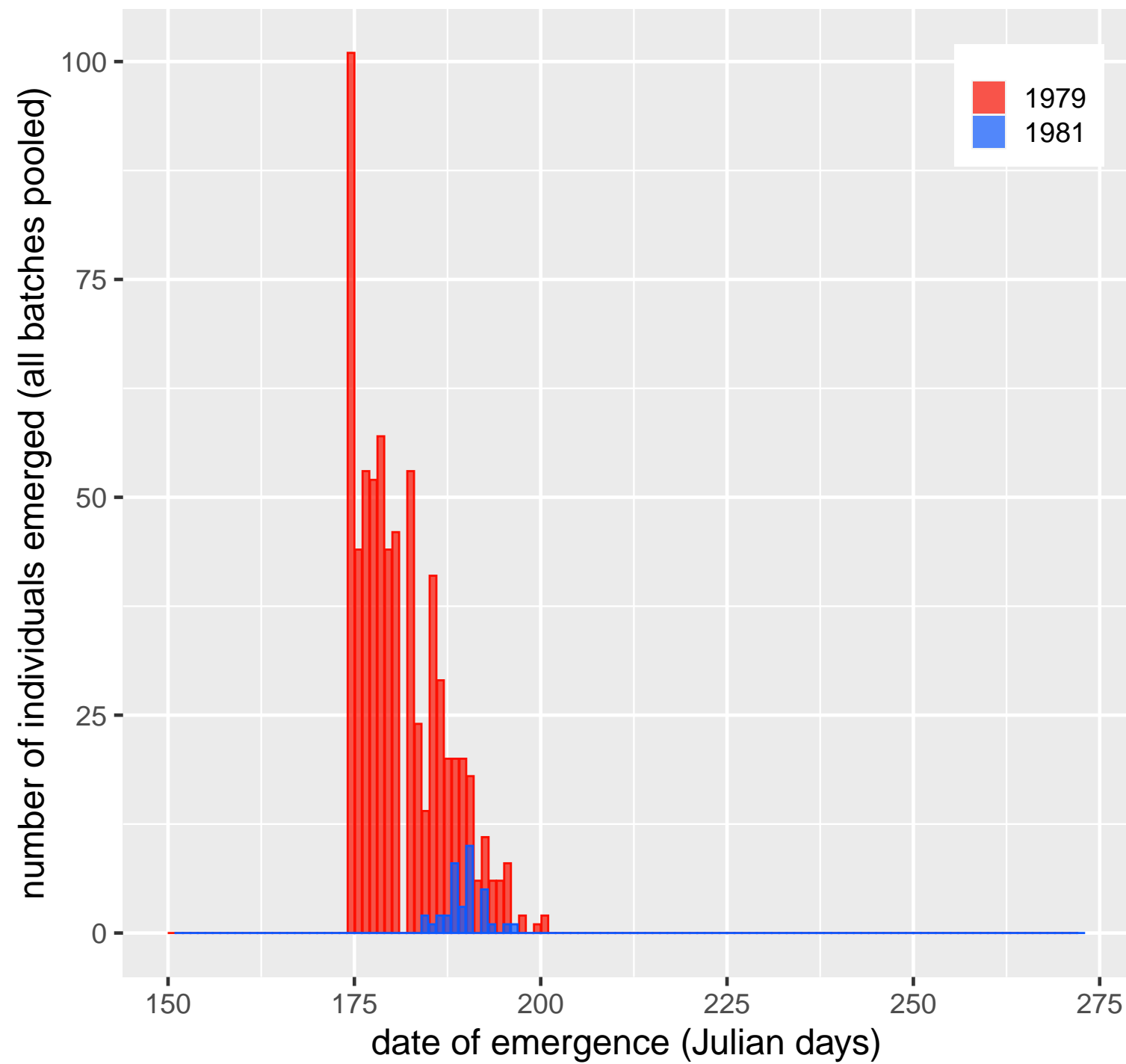

# R923 – 1980

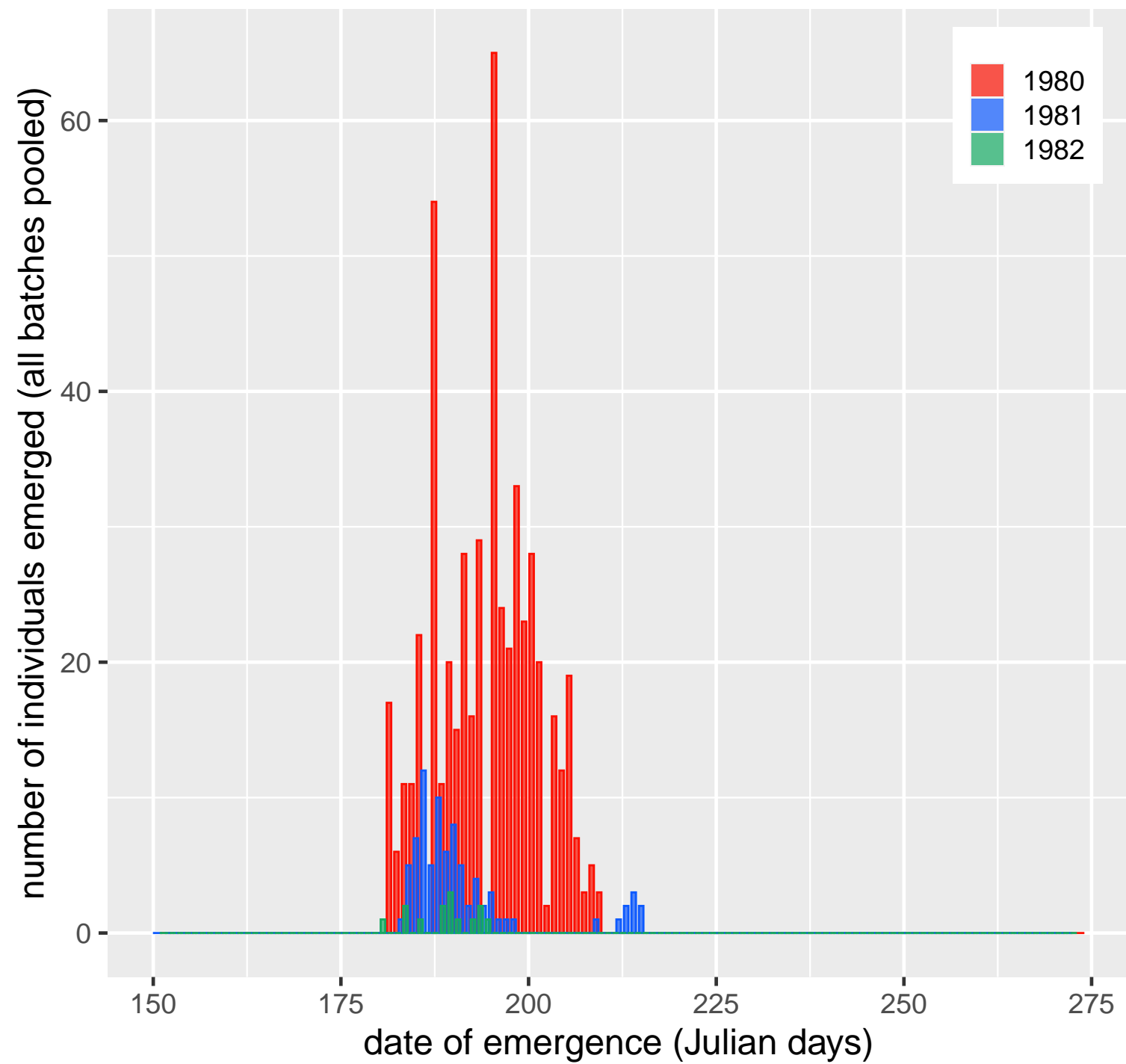



# R923 – 1983

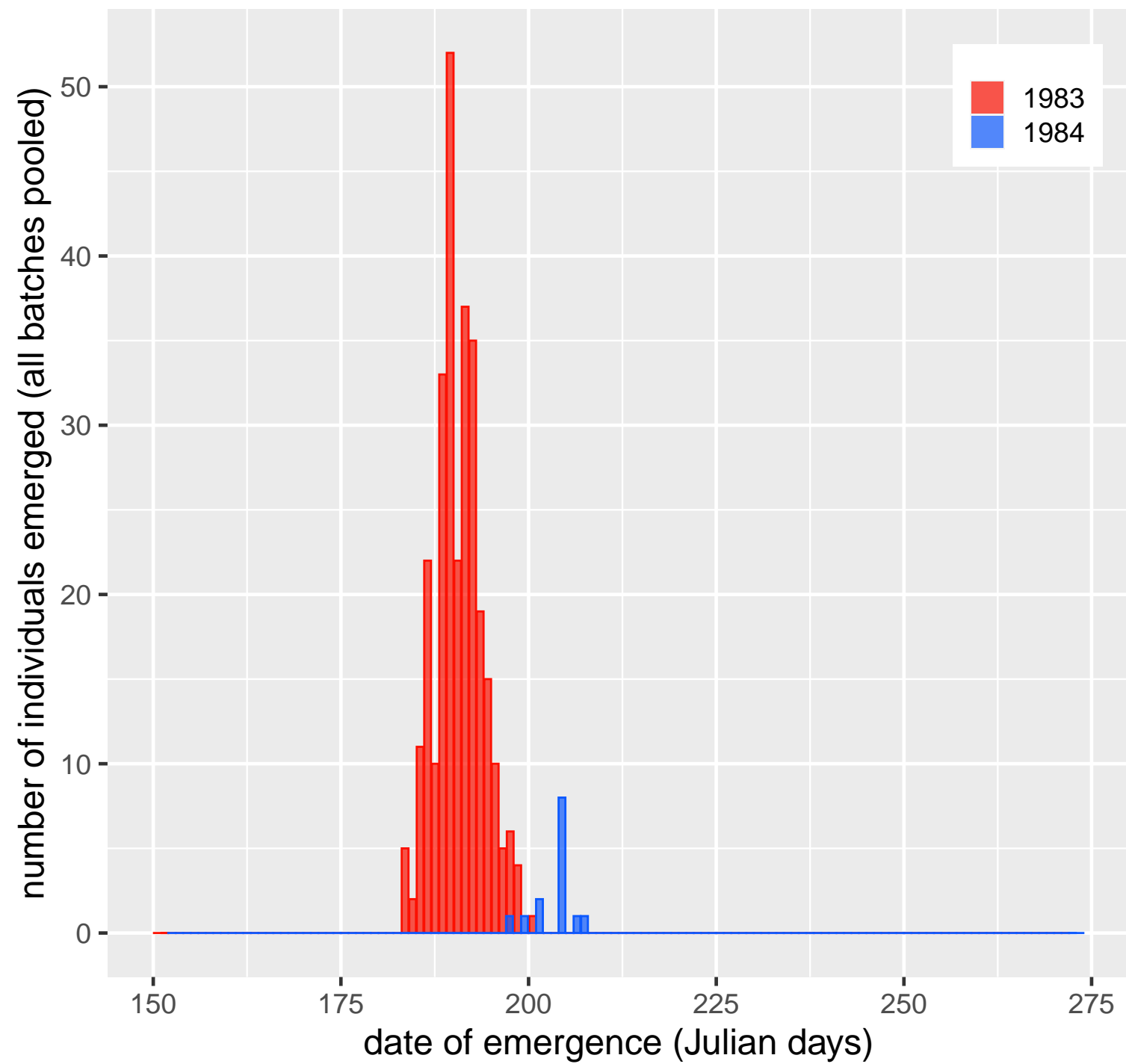

# R923 – 1984

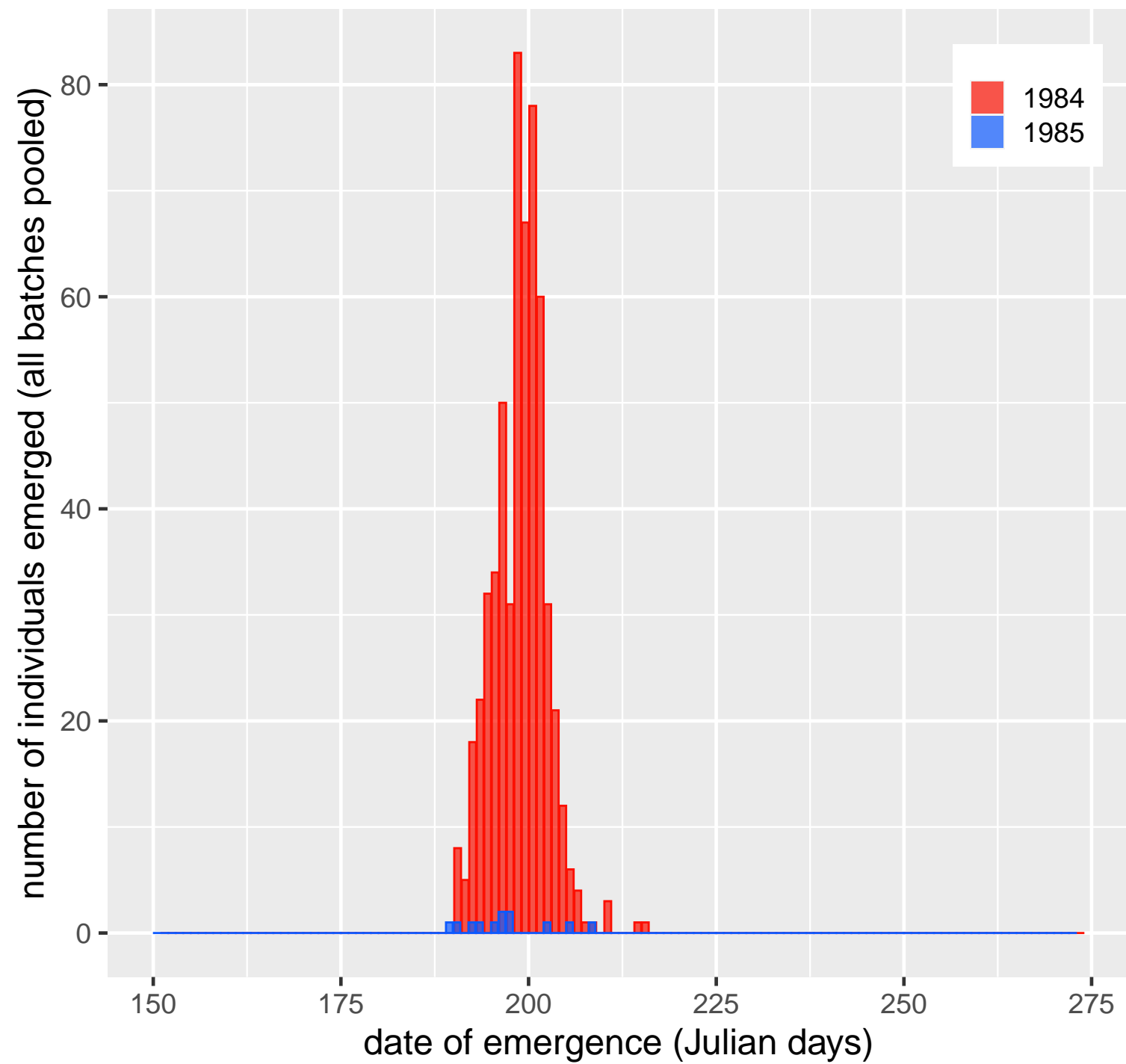

Supplement: Supplementary material 6 — Emergence curves for each cohort sampled in site R923 [file bdj-09-e61086-s006.pdf]

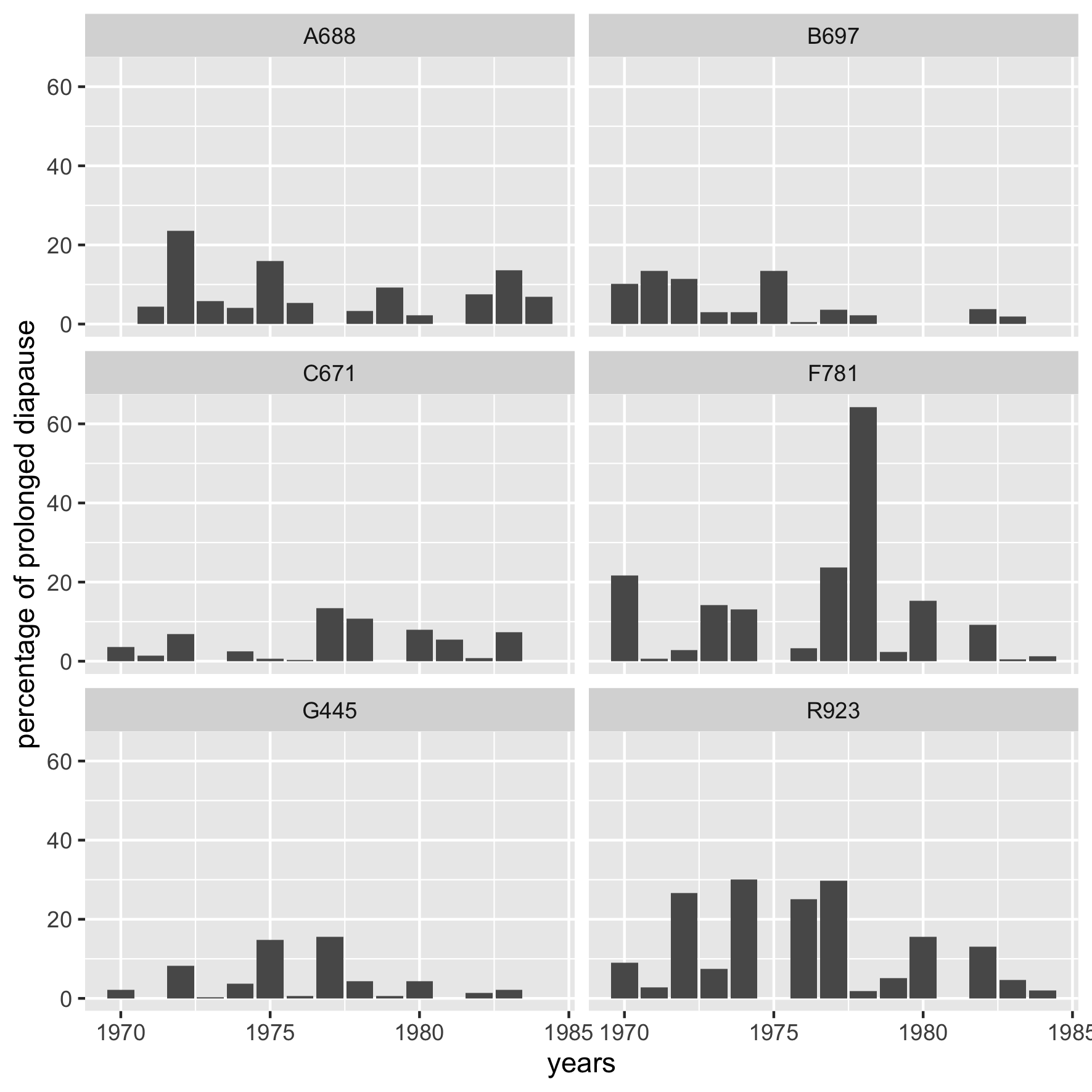

Supplement: Supplementary material 8 — Curves showing the percentage of individuals having experienced a prolonged diapause for each site and year [file bdj-09-e61086-s008.png]
